# Discovery of New 3-(Benzo[*b*]Thiophen-2-yl)Pyrrolidine-2,5-Dione Derivatives as Potent Antiseizure and Antinociceptive Agents—In Vitro and In Vivo Evaluation

**DOI:** 10.3390/ph17111532

**Published:** 2024-11-15

**Authors:** Anna Rapacz, Marcin Jakubiec, Michał Abram, Jakub Jasiński, Karolina Chrzan, Małgorzata Góra, Anna Dziubina, Katarzyna Wójcik-Pszczoła, Paulina Koczurkiewicz-Adamczyk, Katarzyna Ciepiela, Elżbieta Pękala, Jolanta Obniska, Krzysztof Kamiński

**Affiliations:** 1 Department of Pharmacodynamics, Faculty of Pharmacy, Jagiellonian University Medical College, Medyczna 9, 30-688 Krakow, Poland; a.rapacz@uj.edu.pl (A.R.); anna.dziubina@uj.edu.pl (A.D.); 2 Department of Medicinal Chemistry, Faculty of Pharmacy, Jagiellonian University Medical College, Medyczna 9, 30-688 Krakow, Poland; michal.abram@uj.edu.pl (M.A.); jakub45.jasinski@student.uj.edu.pl (J.J.); karolina.chrzan@student.uj.edu.pl (K.C.); gosiag1207@gmail.com (M.G.); aranea.ciepiela@uj.edu.pl (K.C.); k.kaminski@uj.edu.pl (K.K.); 3 Department of Pharmaceutical Biochemistry, Faculty of Pharmacy, Jagiellonian University Medical College, Medyczna 9, 30-688 Krakow, Poland; katarzynaanna.wojcik@uj.edu.pl (K.W.-P.); paulina.koczurkiewicz@uj.edu.pl (P.K.-A.); elzbieta.pekala@uj.edu.pl (E.P.)

**Keywords:** hybrid molecules, antiseizure activity, analgesic activity, peripheral neuropathy, allodynia, pyrrolidine-2,5-dione

## Abstract

**Background/Objectives**: To address the unmet clinical needs in the treatment of epilepsy and pain, the continued development of more effective and safer anticonvulsants and analgesics is still necessary. Therefore, herein we report synthesis and antiseizure/antinociceptive evaluation of a focused series of 3-(benzo[b]thiophen-2-yl)pyrrolidine-2,5-dione derivatives. **Methods**: The anticonvulsant properties were investigated in acute models of seizures, namely the maximal electroshock (MES), the 6 Hz (32 mA), and subcutaneous pentylenetetrazole (*sc*PTZ) seizure models, whereas analgesic activity was tested in the model of a tonic pain/formalin test and oxaliplatin-induced neuropathic pain (in CD-1-mice, i.p.). In addition, a number of in vitro assays were performed, aiming at the evaluation of the drug-like properties of the compounds disclosed herein. **Results**: We identified **33** as a lead compound with the most promising antiseizure properties, i.e., ED_50_ (MES) = 27.4 mg/kg and ED_50_ (6 Hz, 32 mA) = 30.8 mg/kg. Furthermore, **33** at a dose of 100 mg/kg significantly prolonged the latency time to the first seizure episode in the *sc*PTZ model and at high doses did not impaire coordination of mice in the rotarod test (TD_50_ > 200 mg/kg). Apart from broad antiseizure protection, **33** demonstrated a significant analgesic effect in the formalin test (45 mg/kg, i.p.), and effectively alleviated allodynia in the oxaliplatin-induced neuropathic pain model (30 and 45 mg/kg). The binding assays suggest that the most plausible mechanism of action relies on interaction with the neuronal voltage-sensitive sodium channel (site 2). Furthermore, the drug-like potential of **33** supports favorable in vitro results, i.e., no hepatocytotoxicity and neurocytotoxicity at a high concentration of 100 μM, as well as a lack of mutagenicity at a concentration as high as 500 μM. **Conclusions**: Compound **33** identified in the current studies is proposed to be an interesting candidate for further preclinical development as therapy for epilepsy and neuropathic pain.

## 1. Introduction

Epilepsy, which affects more than 70 million people globally, is a common and debilitating neurological disorder with a multi-factorial and complex pathophysiology. This heterogeneous disease is characterized by spontaneous and recurrent seizures which may appear in any moment of the lifespan [1]. For many years, epilepsy was recognized as a disease of the young; however, most recent data show an increased prevalence in the older population. Consequently, this latter fact, together with the aging of societies in industrialized countries, make epilepsy a serious pharmacotherapeutic and economical issue. Despite the dynamic progress in epilepsy research, the approval of more than 30 antiseizure medications (ASMs) over recent decades, and plenty of compounds in development in preclinical and clinical trials [2], approximately 30% of patients still suffer from uncontrolled seizures and are diagnosed with drug-resistant epilepsy (DRE) [3]. The mechanisms underlying the drug resistance phenomenon in epilepsy are very complex and still poorly explained; therefore, the selection of ASMs to provide optimal therapeutic effect remains challenging. Therefore, the development of new, more effective and/or safer ASMs creates invariably an urgent unmet clinical need.

Neuropathic pain is another serious neurological condition which affects 7–10% of the general population, among which 20–30% experience chronic pain. In this group, only half of the patients experience relief through pharmacological treatment, which typically reduces pain by 30–50% [4]. Currently, ASMs (such as pregabalin and gabapentin) and antidepressants (including amitriptyline, nortriptyline, and duloxetine), are the most commonly prescribed treatments for managing neuropathic pain [5,6]. It is postulated that epilepsy and neuropathic pain may have similar neurobiological underpinnings, as reflected by the use of the same drugs [7,8,9]. Therefore, it seems reasonable and fully justified to assess investigational anticonvulsant drugs for their potential effectiveness in neuropathic pain models as well.

**Figure 1:**
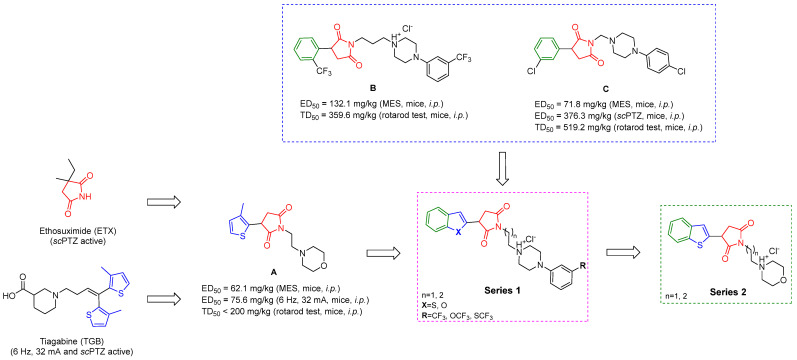
Rationale of design strategy leading to compounds from Series 1 and 2.

Bearing in mind the complexity and heterogeneity of epilepsy (and pain), one of the most recent discovery paradigms assumes the development of so-called multi-functional compounds as promising candidates for new ASMs [10]. These molecules, specifically with hybrid structures, are usually designed applying a framework combination approach which relies on the integration of at least two pharmacophores on one chemical scaffold [11]. Therefore, applying the aforementioned approach, we have obtained in recent years plenty of structurally diversified pyrrolidine-2,5-dione derivatives with a hybrid structure which integrate fragments of well-known ASMs such as ethosuximide (ETX), lacosamide (LCS), or levetiracetam (LEV). Consequently, in vivo evaluations showed that some of these compounds revealed potent and broad-spectrum anticonvulsant activity in the ‘classical’ animal models of seizures, i.e., the maximal electroshock (MES) test, the 6-Hz psychomotor seizure model (at current intensity of 32 mA), and/or the subcutaneous pentylenetetrazol (*sc*PTZ) test [12,13,14,15,16]. One of the most recent chemical groups explored by our team was focused on compounds containing the methyl-thiophene moiety which is a key structural element for the clinically relevant ASM tiagabine (Figure 1). In this series, the most promising antiseizure properties revealed compound **A** (published as **4** in ref. [12]), which effectively protected mice in the MES (ED_50_ of 62.1 mg/kg) and 6 Hz (ED_50_ of 75.6 mg/kg) seizure models. Therefore, with an aim of further investigating the influence of the thiophene (marked in blue in Figure 1) ring and its derivatives on antiseizure efficacy, we decided herein to obtain a new and target library of eight pyrrolidine-2,5-diones (marked in red in Figure 1) containing at position-3 the benzo[b]thiophen-2-yl moiety or its oxygen bioisostere. Additionally, the molecules synthesized herein share structural similarities to potent anticonvulsants identified in a group of *N*-[(4-arylpiperazin-1-yl)-alkyl]-3-phenyl-pyrrolidine-2,5-diones represented by compounds **B**, **C** (published as compounds **11i** and **16**, respectively, in ref. [17,18]). It should be noted that the substitution mode of the phenylpiperazine moiety was restricted only to groups that are favorable to antiseizure activity as identified in the previous studies, i.e., CF_3_, OCF_3_, and SCF_3_ [13,14,16]. This approach allowed for the reduction of the number of animals used in the in vivo experiments.

**Figure 2:**
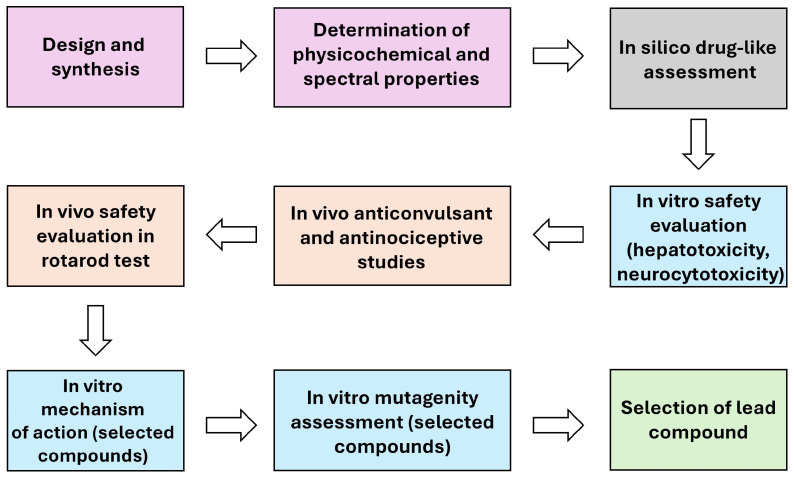
The workflow of the studies performed herein.

The anticonvulsant activity of the new derivatives was assessed in acute models of seizures, namely using the MES, 6 Hz (with 32 and 44 mA stimulus intensity), and *sc*PTZ tests. The antinociceptive properties were estimated in the formalin model of tonic pain as well as in the oxaliplatin-induced neuropathic pain model in mice. Considering drug safety evaluation, which is important during the preclinical characterization of new bioactive substances, the acute neurological toxicity was determined in the rotarod test. Moreover, hepatocytotoxicity and neurocytotoxicity using in vitro cellular models was examined for all compounds prior to in vivo testing. The safety in vitro panel was also extended to the assessment of the mutagenic properties of the most potent antiseizure medications. Finally, for the most potent compounds, in vitro mechanistic studies were performed to establish the plausible mechanism of action underlying their anticonvulsant and antinociceptive properties (for better clarity, the workflow of this project has been presented in Figure 2).

## 2. Results and Discussion

### 2.1. Chemistry

**Scheme 1:**
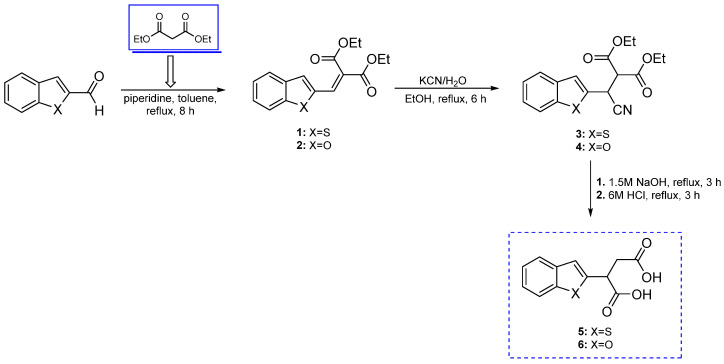
Synthesis of starting and non-commercial dicarboxylic acids **5** and **6**.

The final phenylpiperazine derivatives **25**–**31** (Series 1) and morpholine derivatives **32**, **33** (Series 2) were obtained applying the multi-step synthetic procedure that also involved the preparation of non-commercial acids (**5**, **6**) according to Scheme 1. First, commercial benzo[*b*]thiophene-2-carboxaldehyde and 2-benzofurancarboxaldehyde were simultaneously treated with diethyl malonate, yielding unsaturated diesters (**1**, **2**) in the Knoevenagel condensation. Then, the addition of KCN to **1** and **2** gave respective intermediates **3** and **4**, which were further hydrolyzed to the desired dicarboxylic acids **5** and **6**.

**Scheme 2:**
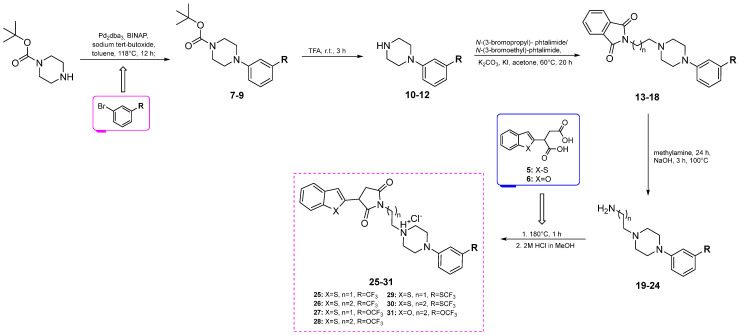
Synthesis of intermediates (**7**–**24**) and final compounds **25**–**31** (Series 1).

The final compounds of Series 1 (**25**–**31**) were obtained according to the synthetic procedure depicted in Scheme 2. Commercially available Boc-piperazine in the *N*-arylation reaction with aryl bromide yielded Boc-protected 4-phenylpiperazine derivatives (**7**–**9**) [19]. Removal of the Boc group in acid conditions (TFA) followed by neutralization with 25% ammonium hydroxide yielded the desired 4-arylpiperazine derivatives (**10**–**12**). Next, intermediates **10**–**12**, using the respective alkylating agents (*N*-(3-bromopropyl)-phthalimide or *N*-(3-bromoethyl)-phthalimide), were converted to phthalimide derivatives **13**–**18**. The amines **19**–**24** were obtained in the aminolysis reaction of **13**–**18** with methylamine. Finally, the desired compounds **25**–**31** were obtained by the cyclocondensation of the appropriate amines **19**–**24** with dicarboxylic acids **5** or **6** and then converted into hydrochloride salts using 2M hydrochloric acid solution in methanol (Sigma-Aldrich, St. Louis, MO, USA).

**Scheme 3:**
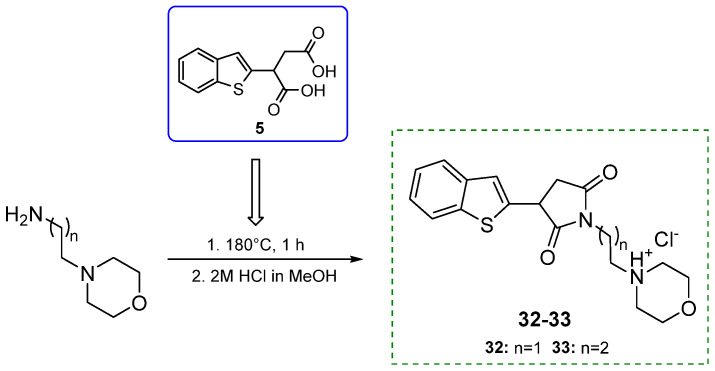
Synthesis of final compounds **32** and **33** (Series 2).

Due to the high antiseizure activity of the morpholine derivatives described by our team previously [12], as well as the aim for a more detailed structure–activity relationship analysis, we decided also to obtain Series 2 of the compounds (**32**, **33**), containing the morpholine ring instead of the phenylpiperazine moiety which appeared in Series 1. Therefore, **32** and **33** were obtained in a two-step procedure involving the condensation of commercially available aminoalkylmorpholine derivatives with dicarboxylic acid **5** and the conversion of crude products into water-soluble hydrochloride salts (**32**, **33**), by using the 2M hydrochloric acid solution in methanol. The synthetic procedure is shown in Scheme 3.

All final compounds were obtained in good yields (>60%). Their structures were confirmed by ^1^H NMR, ^13^C NMR, and ^19^F NMR (for fluorocontaining molecules) spectra analysis, as well as by applying high-resolution mass spectrometry (HRMS). Moreover, for all intermediates and final compounds, the LC-MS spectra were also obtained. Their purity, determined by the UPLC method, was >97%. The physicochemical and spectral data for the intermediates and the final compounds are summarized in Section 3.

### 2.2. In Silico Studies

The physicochemical properties of **25**–**33** were determined based on Lipinski and Veber’s rules using the online tool, the SwissAdme website (Table 1) [20,21].

Lipinski and Veber’s rules are utilized to assess the drug-like characteristics of a chemical compound, which help to determine whether it possesses physicochemical properties favorable for oral absorption in humans. Veber’s requirements are NBR ≤ 10 and TPSA < 140 Å^2^, while Lipinski’s criteria are MW ≤ 500 Da, log P ≤ 5, HBD ≤ 5, and HBA ≤ 10 [12,13]. Although the molecular weight values for compounds **26**–**31** marginally exceeded 500 Da (ranging from 501.50 Da for compound **31** to 533.63 Da for compound **30**, correspondingly), all of the compounds that were presented satisfied the requirements of Veber’s rules and Lipinski’s rules. No HBDs are present in any of the compounds and the number of HBAs ranges from four (for **32** and **33**) to eight (for **31**). Therefore, all of the molecules satisfy the aforementioned drug-likeness requirements, as indicated in Table 1. Furthermore, Instant JChem by ChemAxon software version 23.14 was used to compute the central nervous system multiparameter optimization (CNS MPO) score (Table 1) [22]. Six important physicochemical characteristics are assessed by the well-known CNS MPO score algorithm: ClogP, the estimated distribution coefficient at pH 7.4 (ClogD), TPSA, HBDs, MW, and the most basic center (pKa). Since each property has a value between 0 and 1, the total score can be as high as 6. In CNS drug development programs, a score of ≥4.0 is typically utilized as a cut-off point for choosing compounds for hit finding, and a higher score is desired. The compounds from Series 2 that contained morpholine (**32**, **33**) received the highest scores (6.0). Compound **31** received the highest score of 3.59, which is quite near the cut-off mark, among the phenylpiperazine derivatives (Series 1), whereas the CNS MPO parameters for the remaining drugs fell between 2.75 and 3.35.

### 2.3. In Vitro Hepatocytotoxicity and Neurocytotoxicity Assays

**Figure 3:**
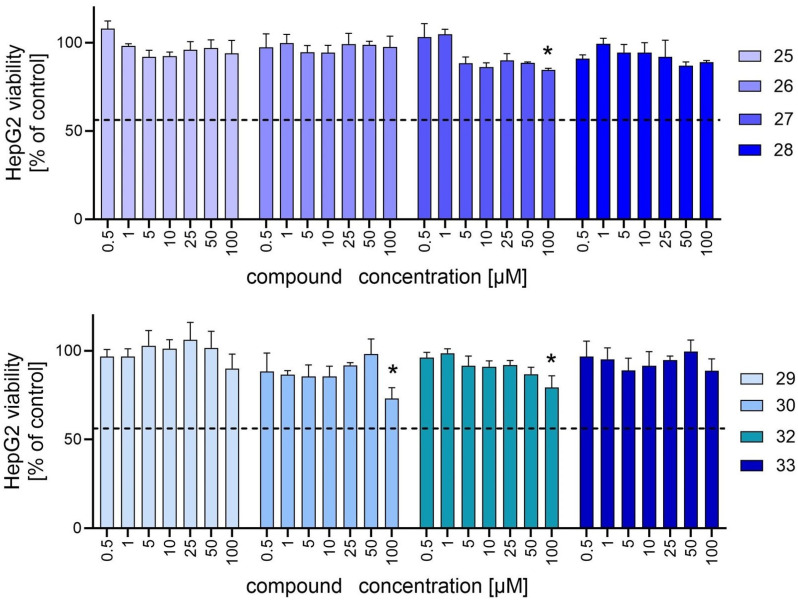
Compounds **25**–**30**, **32**, and **33** do not demonstrate hepatocytotoxicity in vitro. HepG2 cells were exposed to growing concentrations (0.5–100 µM) of tested compounds for 24 h. Cell viability was measured by MTT assay. Bars represents mean percent of cells viability normalized to non-treated cells (100%) ± SEM. Dashed line indicates toxicity of doxorubicin, applied at 10 µM. * *p* < 0.05.

**Figure 4:**
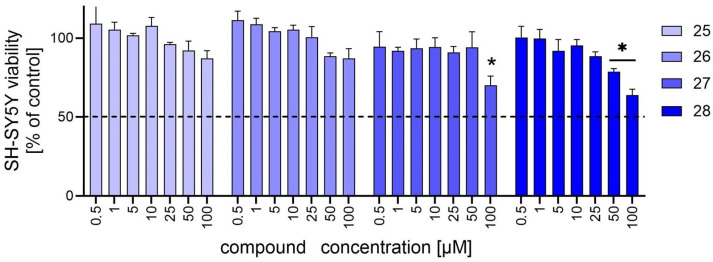

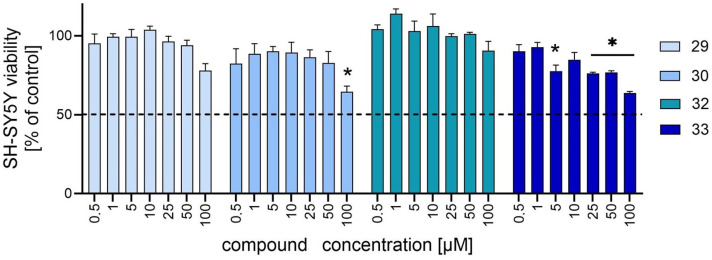
Compounds **25**–**30**, **32**, and **33** do not demonstrate neurocytotoxicity in vitro. SH-SY5Y cells were exposed to growing concentrations (0.5–100 µM) of tested compounds for 24 h. Cell viability was measured by MTT assay. Bars represents mean percent of cells viability normalized to non-treated cells (100%) ± SEM. Dashed line indicates toxicity of doxorubicin, applied at 10 µM. * *p* < 0.05.

Preliminary in vitro hepatocytotoxicity and neurocytotoxicity studies were performed using the commercially available cell lines HepG2 (Figure 3) and SH-SY5Y (Figure 4), respectively. The obtained results allow us to conclude that the tested derivatives do not show a toxic effect on both human hepatocellular carcinoma cells and neuroblastoma cells. The viability of cells incubated in the presence of **25**–**30**, **32**, and **33** at a concentration of 10 μM was definitely higher than the viability of cells exposed to the positive control used in the study, a known chemotherapeutic drug doxorubicin.

### 2.4. Anticonvulsant Activity

The initial neurotoxicity screening (NT) of all nine tested compounds at a fixed dose of 100 mg/kg was performed using the rotarod test 2 min before the MES test. Only two tested compounds, **25** and **31**, impaired motor coordination of 50% of the animals in the rotarod test. Then, antiseizure screening in the MES test, which is a model of generalized tonic/clonic seizures, showed that four compounds, **28**, **30**, **31**, and **33**, protected all mice against the occurrence of tonic seizures. Moreover, two compounds, **27** and **32**, protected 50% of the animals tested. In the next step, nine compounds, also at a screening dose of 100 mg/kg, were screened in the 6 Hz test with a 32 mA stimulus intensity, which is a model of focal (partial) seizures. In this test, one compound, **31**, protected all of the mice; three compounds, **28**, **32**, and **33**, protected 75% of the mice; and three compounds, **25**, **27** and **30**, protected 50% of the tested animals, whereas **26** and **29** were inactive in this test. Subsequently, four compounds, **28**, **30**, **32**, and **33**, were also tested at a fixed dose of 100 mg/kg in the 6 Hz test with a 44 mA stimulus intensity—a model of pharmacoresistant focal seizures. However, none of the tested compounds demonstrated significant activity in this test. The preliminary results of the NT, MES, and 6 Hz tests are presented in Table S1.

Based on the preliminary results from the MES and 6 Hz (32 mA) tests, the median effective doses (ED_50_) were determined for the most promising compounds (**28**, **30**, **31**, **32**, and **33**). In the MES test, the lowest ED_50_ value was obtained for compound **33**, followed by **31**, **28**, **30**, and **32**. Similarly, in the 6 Hz test, compound **33** turned out to be the most active, followed by compounds **31**, **32**, **28**, and **30**. Additionally, in order to determine the TD_50_ dose, the effect of four compounds, **28**, **30**, **32**, and **33**, on the motor coordination of mice was assessed in a rotarod test at a dose of 200 mg/kg and no symptoms of neurotoxicity were observed; hence, the TD_50_ value for these compounds was assumed to be >200 mg/kg. In the case of compound **31**, for which neurotoxicity symptoms were observed in two mice out of four at a screening dose of 100 mg/kg, further studies allowed the calculation of the TD_50_ dose, which was 120.92 mg/kg. Comparing the values of the protective indexes (PIs) in the MES test for the tested and reference compounds, the highest PI was shown by compound **33**, followed by **28**, **30**, **31**, **32**, and valproic acid (VPA), while in the 6 Hz test, the PI value was the highest for LEV, followed by **33**, **32**, **28**, **30**, **31**, VPA, and ETX. It is noteworthy that these new molecules showed higher activity (lower ED_50_ values) and higher PIs in comparison with the active ASMs VPA and ETX. Moreover, unlike ETX and LEV, which are inactive in the MES test, these tested hybrid compounds demonstrated anticonvulsant activity in two tests, the MES and 6 Hz (32 mA) models of generalized and focal seizures, respectively. Both the ED_50_ and TD_50_ values with 95% confidence limits were calculated by probit analysis and are presented together with the PI values in Table 2.

**Figure 5:**
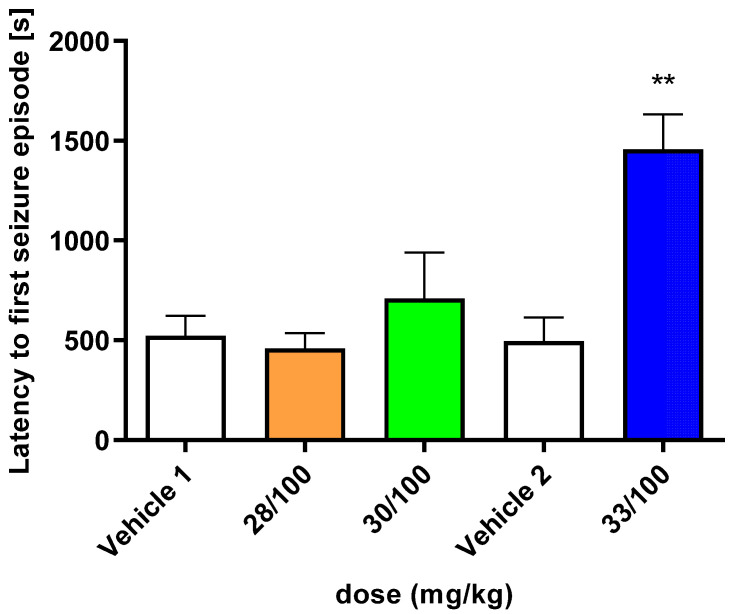
The effect of compounds **28**, **30**, and **33** at a dose of 100 mg/kg (i.p.) on the latency to first seizure episode in the *sc*PTZ test. The compounds or the vehicle (1% Tween 80) was administered 30 min i.p. before the test. The results are presented as bar plots showing the mean ± SEM. The statistical significance was evaluated by an unpaired *t* test (for comp. **33**), which resulted in ** *p* < 0.01, and a one-way ANOVA test (for compounds **28** and **30**) which indicated no significance at n = 8 mice per group.

In the next phase of the antiseizure activity studies, compounds **28**, **30**, and **33** were selected for further investigation due to their potent protection in the MES and 6 Hz models as well as their favorable protective index. Thus, they were examined via chemically induced seizures by subcutaneous (*s.c.*) administration of pentylenetetrazole (PTZ)in an *sc*PTZ test, which is a model of absence and myoclonic seizures. PTZ is a convulsant substance which antagonizes the inhibitory function of GABA by binding to the picrotoxin-binding site of the postsynaptic GABA_A_ receptor [24]. In this test, **33** significantly prolonged the latency time to the first seizure episode compared to the vehicle group by 196%, *p* < 0.01 (control group: 496.2 ± 117.3 s; **33**: 1470 ± 270.4 s) t = 4.539, df = 10. On the other hand, compounds **28** and **30** were inactive in this test (Figure 5).

Summing up, of the tested compounds, four of them (**28**, **30**, **31**, and **32**) demonstrated anticonvulsant activity in two models of epileptic seizures (a model of generalized tonic/clonic seizures and a model of focal seizures), while compound **33** also showed activity in a model of absence seizures, presenting a broad spectrum of anticonvulsant effects in mice. Therefore, compound **33** was selected for further studies to evaluate its analgesic activity.

### 2.5. Antinociceptive Activity

#### 2.5.1. Formalin Test

The formalin test, which is a model of tonic pain, is often used for screening new compounds with probable antinociceptive activity, and some of its mechanisms resemble those underlying clinical pain in humans [25,26]. Immediately upon injection, formalin triggers a biphasic nocifensive behavioral response (an early and a late phase) of strong, nociceptive behavior, characterized by the animal licking, biting, and shaking its injected paw. The acute nociceptive phase lasts for the first 5 min and is associated with immediate activation of nociceptors (neurogenic pain), whereas the second inflammatory phase occurs between 15 and 30 min after formalin injection, which is associated with an inflammatory response to tissue damage and involves the sensitization of spinal reflex circuits [27,28]. It has also been suggested that formalin injection results in pathological changes that resemble those observed in nerve injury and neuropathic pain [29]. Additionally, numerous preclinical studies have demonstrated the analgesic activity of ASMs in both phases of the formalin test or only in the second (inflammatory) phase [27,30]. Thus, in the next step of our study, we decided to check the activity of the most promising compound, **33**, characterized by both potent antiseizure activity and favorable protective indexes. Our previous studies have shown that pregabalin, which is recommended as a first-line treatment in neuropathic pain in humans [31], used as the reference drug at a dose of 30 mg/kg, significantly reduced the duration of the pain response in the second phase of the formalin test. Therefore, in the present study, to evaluate the analgesic activity of compound **33**, a dose of 30 mg/kg was also used initially, and then a higher dose of 45 mg/kg was applied [32,33]. Moreover, it is worth noting that in the anticonvulsant activity studies, the ED_50_ values for compound **33** were close to 30 mg/kg; namely, in the MES test, it was 24.1 mg/kg, and in the 6 Hz test, it was 30.8 mg/kg.

**Figure 6:**
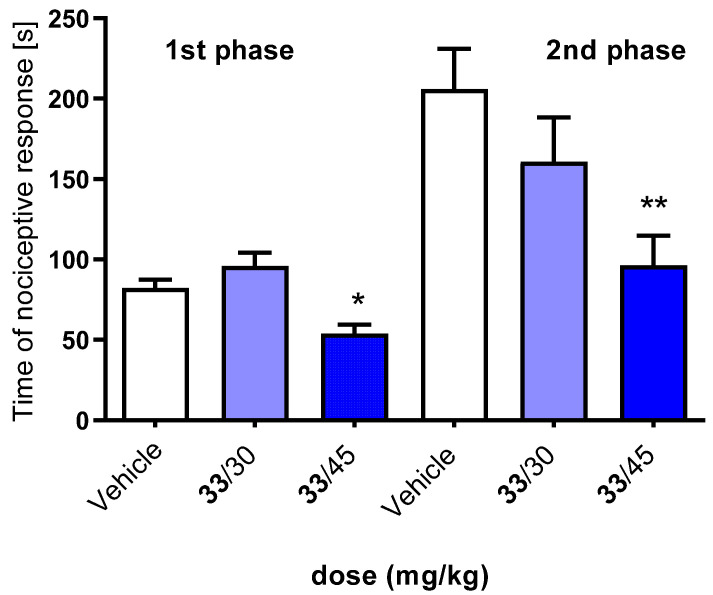
The effect of compound **33** (30 and 45 mg/kg, i.p.) on the duration of licking/biting behavior in the acute phase (0−5 min after formalin injection) and in the late phase (15−30 min after formalin injection). The test compound or vehicle (1% Tween 80) was administered 30 min i.p. before the test. The results are presented as bar plots showing the mean ± SEM. The statistical significance was evaluated by one-way ANOVA followed by Dunnett’s post hoc test: * *p* < 0.05, ** *p* < 0.01, n = 8 mice per group.

As shown in Figure 6, compound **33**, at the dose of 45 mg/kg, significantly reduced the time of the nociceptive response in both phases I (neurogenic—early phase) and II (inflammatory—second phase) of the formalin test by 34% (*p* < 0.05) F (2, 21) = 10.64 and by 53.3% (*p* < 0.01) F (2, 21) = 5.233, respectively. At the lower dose of 30 mg/kg, it did not significantly affect the time of the pain reaction, neither in the early nor in the second phase of the test.

Such results revealed a possible wide spectrum of both analgesic and anti-inflammatory properties of the tested compound. The activity in the second phase might be of great therapeutic value in terms of the compound’s possible use in the treatment of neuropathic pain.

#### 2.5.2. Oxaliplatin-Induced Neuropathic Pain

Therefore, in the next step, we assessed the antiallodynic effect of compound **33** in the oxaliplatin-induced neuropathic pain model of chemotherapy-induced peripheral neuropathy.

In this test, in non-treated mice, the average threshold of pain sensitivity for mechanical stimulation was 2.81 ± 0.18 g, whereas in oxaliplatin-treated mice, the sensitivity to pain increased significantly, which lowered the threshold of mechanical nociception in the acute phase to 1.73 ± 0.03 g and in the late phase to 1.80 ± 0.05 g.

**Figure 7:**
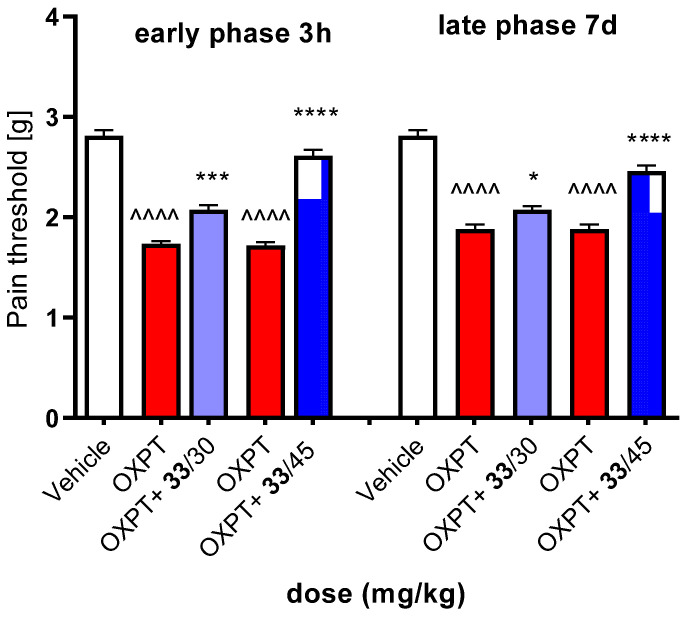
The antiallodynic effects of compound **33** (30 and 45 mg/kg, i.p.) in the tactile allodynia in oxaliplatin (OXPT)-induced peripheral neuropathy. The compound was administered 30 min before the evaluation in the von Frey test carried out 3 h and 7 days after OXPT injection. The results are presented as bar plots showing the mean ± SEM. The statistical significance was evaluated by repeated measures ANOVA, followed by Tukey’s multiple comparison: * *p* < 0.05, *** *p* < 0.01, and **** *p* < 0.0001 when the results are compared to the OXPT-treated group, and ^^^^ *p* < 0.0001 when the results are compared to naive mice, n = 10 mice per group.

As shown in Figure 7, in the early phase of the test, compound **33** at doses of 30 mg/kg and 45 mg/kg elevated the pain threshold by 19.6% (*p* < 0.001) F (2, 27) = 152.5 and by 51.9% (*p* < 0.0001) F (2, 27) = 126.3, respectively. Seven days after the administration of the oxaliplatin, in the late phase of the test, a reduction of allodynia by 10% (*p* < 0.05) F (2, 27) = 109.3 and by 30.7% (*p* < 0.0001) F (2, 27) = 76.64 was observed for **33** at doses of 30 mg/kg and 45 mg/kg, respectively.

It is worth emphasizing that compound **33** at a dose of 45 mg/kg almost completely abolished allodynia in both the acute and late phases. Moreover, at lower dose of 30 mg/kg, significant antiallodynic activity was also observed.

Summing up the results of analgesic tests, we found out that compound **33** at a dose of 45 mg/kg demonstrated broad spectra of activity attenuating neurogenic and inflammatory pain in the formalin test as well as neuropathic pain in the model of chemotherapy-induced peripheral neuropathy.

Bearing in mind that no neurotoxicity was observed for compound **33** even at the highest tested dose of 200 mg/kg, whereas analgesic activity was observed at a dose of 30 and/or 45 mg/kg, it can be concluded that the tested compound has a large margin of safety.

### 2.6. In Vitro Sodium and Calcium Channel Binding Studies

Given the fact that in establishing and regulating the excitability of CNS neurons, as well as in the suppression of seizures, sodium and calcium channels play a fundamental role, it is important to know the possible molecular targets by which the tested molecules might act; for molecules **32** and **33**, the binding assays for the sodium channel (site 2), and L-type calcium channels were carried out using [^3^H]batrachotoxin and [^3^H]nitrendipine as radioligands, respectively [34,35]. Compound **33** at the concentration of 100 µM revealed a high inhibition of the sodium channel (site 2), as is indicated by an inhibition greater than 50%, whereas **32** did not bind to these channels significantly. At this concentration, **33** influenced sodium channels in a similar manner to phenytoin and more than carbamazepine. Regarding the impact on calcium channels, **33** did not bind effectively to L-type calcium channels (Table 3). However, the results were higher compared to topiramate, an ASM, which has a diverse mechanism of action, including a modulation of neuronal voltage-gated calcium channels [36]. The findings from electrophysiological studies suggest that the mechanism of anticonvulsant action is connected with the inhibition of sodium and/or L-type calcium voltage-gated channels. The influence on sodium channels may also be the mechanism of antinociceptive activity of compound **33** [37,38,39]. However, a fuller understanding of the mechanism of action of **33** requires further research, including the involvement of a specific subtype of sodium and calcium channels and other ion channels (e.g., TRPV1, potassium channels), the effect on GABAergic transmission or influence on ionotropic glutamate receptors, or the direct modulation of synaptic neurotransmitter release, e.g., glutamate.

### 2.7. Analysis of Mutagenicity

In the present study, an Ames MPF 98/100-1 + S9 + KP microplate format mutagenicity assay (Xenometrix, Allschwil, Switzerland) was used to evaluate the potential mutagenicity of compound **33**, which showed the most potent anticonvulsant activity and the most beneficial PIs, as well as its less active close analog, compound **32** (for details see Table 4). According to the obtained results, the analyzed compounds used at concentrations of 100, 200, or 500 µM do not show two-fold induction over the baseline, and a dose-dependent response was not observed in either the absence or presence of metabolic activation. Therefore, both compounds were safe and non-mutagenic in the absence and presence of metabolic activation.

## 3. Materials and Methods

### 3.1. Synthesis

All chemicals and solvents were purchased from commercial suppliers and were used without further purification. Melting points (mp.) were determined in open capillaries on a Büchi 353 melting point apparatus (Büchi Labortechnik, Flawil, Switzerland). The purity and homogeneity of the compounds was assessed by thin-layer chromatography (TLC) and gradient ultra-performance liquid chromatography (UPLC). Thin-layer TLC was carried out on silica gel 60 F–254 pre-coated aluminum sheets (Macherey-Nagel, Düren, Germany), using the following developing systems: S_1_-DCM (Dichlotomethane):MeOH (Methanol) (9:0.3; *v*/*v*); S_2_-DCM:MeOH (9:0.4; *v*/*v*); S_3_-DCM:MeOH (9:0.5; *v*/*v*); S_4_-DCM:MeOH (9:0.7; *v*/*v*); S_5_-DCM:MeOH (9:1; *v*/*v*). (Spots detection: UV light (λ = 254 nm). The UPLC analyses and mass spectra (LC-MS) were obtained on Waters ACQUITY™ TQD system (Waters, Milford, CT, USA) with the MS-TQ detector (Waters, Milford, CT, USA) and UV-Vis-DAD eλ detector (Waters, Milford, CT, USA). The ACQUITY UPLC BEH C18, 1.7 μm (2.1 × 100 mm) column was used with the VanGuard Acquity UPLC BEH C18, 1.7 μm (2.1 × 5 mm) (Waters, Milford, CT, USA). Standard solutions of each compound were prepared in analytical-grade MeCN/water mixture (1:1; *v*/*v*). Conditions applied were as follows: eluent A (water/0.1% HCOOH), eluent B (MeCN/0.1% HCOOH), a flow rate of 0.3 mL/min, a gradient of 5–100% B over 10 min, and an injection volume of 10 µL. The UPLC analyses and high-resolution mass spectra (LC-HRMS) were obtained using a Waters ACQUITY I-Class PLUS SYNAPT XS High-Resolution Mass Spectrometer (Waters, Milford, CT, USA) with an MS-Q-TOF detector (Waters, Milford, CT, USA) and a UV–vis-DAD eλ detector (Waters, Milford, CT, USA). The UPLC retention times (*t*_R_) are given in minutes. The purity of target compounds determined by use of chromatographic UPLC method was ≥97%. Preparative column chromatography was performed using silica gel 60 (particle size 0.063–0.200; 70-230 Mesh ATM) purchased from Merck (Darmstadt, Germany). The ^1^H NMR and ^13^C NMR spectra were obtained using a JOEL-500 spectrometer (JEOL USA, Inc., Peabody, MA, USA), in CDCl_3_ and DMSO-d_6_ operating at 500 MHz (^1^H NMR) and 126 MHz (^13^C NMR). Chemical shifts are reported in δ values (ppm) relative to tetramethylsilane (TMS) δ = 0 as internal standard. The J values are expressed in Hertz (Hz). Signal multiplicities were represented by the following abbreviations: s (singlet), br s (broad singlet), d (doublet), dd (double doublet), ddd (double double doublet), dt (doublet of triplets), t (triplet), td (triplet of doublets), q (quartet), quin (quintet), quind (quintet of doublets), m (multiplet).

#### 3.1.1. Synthetic Procedure for Diethyl Malonate Derivatives **1** and **2**

A solution of either benzo[*b*]thiophene-2-carboxaldehyde (15 g, 92.47 mmol, 1 eq) or 2-benzofurancarboxaldehyde (13.51 g, 92.47 mmol, 1 eq) in AcOH (3 mL), piperidine (3 mL) and toluene (80 mL) was treated dropwise with diethyl malonate (18.96 g, 118.36 mmol, 1.28 eq). After stirring in reflux for about 8 h, the mixtures were cooled and concentrated under reduced pressure. The residues were dissolved in DCM (80 mL) and extracted with saturated NaHCO_3_ (3 × 50 mL), 1M HCl (3 × 50 mL), and water (3 × 50 mL). The organic layers were dried with anhydrous Na_2_SO_4_ and the solvents were evaporated in vacuum. Diethyl malonate derivatives **1** and **2** were obtained as yellow solids.

Diethyl 2-(benzo[*b*]thiophen-2-ylmethylene)malonate (**1**).

Yellow solid. Yield: 65% (16.95 g); UPLC (purity = 92.67%): t_R_ = 6.35 min. C_16_H_16_O_4_S (304.36). LC-MS (ESI): *m*/*z* calcd for C_16_H_16_O_4_S [M+H]^+^ 305.80, found 305.9.

Diethyl 2-(benzofuran-2-ylmethylene)malonate (**2**).

Yellow solid. Yield: 61% (14.85 g); UPLC (purity = 91.34%): t_R_ = 6.87 min. C_16_H_16_O_5_ (288.30). LC-MS (ESI): *m*/*z* calcd for C_16_H_16_O_5_ [M+H]^+^ 289.10, found 289.2.

#### 3.1.2. Synthetic Procedure for Acids **5** and **6**

KCN (3.16 g, 48.56 mmol, 1 eq) dissolved in water was added to a solution of either **1** (14.78 g, 48.56 mmol, 1 eq) or **2** (14 g, 48.56 mmol, 1 eq) in EtOH (100 mL), and the reactions were then stirred in reflux for about 6 h, which allowed us to obtain intermediate products **3** and **4**. Then, both mixtures without any preparations were treated with a solution of NaOH (2.50 g, 58.27 mmol, 1.2 eq) in water (30 mL) and were then refluxed for a further 3 h. After this time, water (100 mL) was added to the mixtures to maintain a constant volume and the ethanol was evaporated in vacuo. The reaction mixtures were treated with 6 M HCl (35 mL) to reach pH = 1 and stirred in reflux for 3 h. After cooling to room temperature, both were left in a refrigerator for 12 h. The aqueous solutions of **5** and **6** were extracted with ethyl acetate (3 × 50 mL). The organic phases were washed with saturated NaHCO_3_ (3 × 50 mL). Then, aqueous phases were combined and treated with 3M HCl to reach pH = 1 and extracted with ethyl acetate (3 × 50 mL). The organic layers were dried over anhydrous Na_2_SO_4_ and evaporated to dryness to give a yellow solid, which was then purified by column chromatography using the following developing system: S_5_.

2-(Benzo[b]thiophen-2-yl)succinic acid (**5**).

Yellow solid. Yield: 65% (7.68 g); TLC: R_f_ = 0.22 (S_5_); UPLC (purity = 97.23%): t_R_ = 5.49 min. C_12_H_10_O_4_S (250.27). LC-MS (ESI): *m*/*z* calcd for C_12_H_10_O_4_S [M-H]^+^ 250.03, found 250.3.

2-(Benzofuran-2-yl)succinic acid (**6**).

Yellow solid. Yield: 62% (6.84 g); TLC: R_f_ = 0.23 (S_5_); UPLC (purity = 97.02%): t_R_ = 6.37 min. C_12_H_10_O_5_ (234.21). LC-MS (ESI): *m*/*z* calcd for C_12_H_10_O_5_ [M-H]^+^ 234.05, found 234.2.

#### 3.1.3. Synthetic Procedure for Boc-Protected Derivatives (**7**–**9**)

The starting (non-commercial) Boc-derivatives of 4-aryl-piperazine were obtained in *N*-arylation reaction according to Scheme 1. The appropriate aryl bromide (12 mmol, 1 eq), Pd_2_dba_3_ (0.44 g, 0.48 mmol, 0.04 eq), BINAP (0.44 g, 0.7 mmol, 0.06 eq), sodium tert-butoxide (1.62 g, 16.8 mmol, 1.4 eq), and Boc-piperazine 4,48 g, 24 mmol, 2 eq) were suspended in an inert gas (argon) atmosphere in 15 mL of dry toluene. Next, the reaction mixture was refluxed for 12 h, subsequently cooled, and filtered through Celite 545 Merck (Darmstadt, Germany). Next, filtrate was concentrated under reduced pressure. The Boc-protected amines **7**–**9** were purified by column chromatography using the following developing system: S_1_.

*Tert*-butyl 4-(3-(trifluoromethyl)phenyl)piperazine-1-carboxylate (**7**).

Light oil. Yield: 68% (2.62 g); TLC: R_f_ = 0.82 (S_1_); UPLC (purity = 97.24%): t_R_ = 8.45 min. C_16_H_21_F_3_N_2_O_2_ (330.16). LC-MS (ESI): *m*/*z* calcd for C_16_H_21_F_3_N_2_O_2_ [M+H]^+^ 331.16, found 331.2.

*Tert*-butyl 4-(3-(trifluoromethoxy)phenyl)piperazine-1-carboxylate (**8**).

Light oil. Yield: 71% (2.91 g); TLC: R_f_ = 0.79 (S_1_); UPLC (purity = 98.54%): t_R_ = 8.73 min. C_16_H_21_F_3_N_2_O_3_ (346.15). LC-MS (ESI): *m*/*z* calcd for C_16_H_21_F_3_N_2_O_3_ [M+H]^+^ 347.15, found 347.2.

*Tert*-butyl 4-(3-((trifluoromethyl)thio)phenyl)piperazine-1-carboxylate (**9**).

Light oil. Yield: 68% (2.87 g); TLC: R_f_ = 0.80 (S_1_); UPLC (purity = 97.12%): t_R_ = 9.14 min. C_16_H_21_F_3_N_2_O_2_S (362.41). LC-MS (ESI): *m*/*z* calcd for C_16_H_21_F_3_N_2_O_2_S [M+H]^+^ 363.13, found 363.2.

#### 3.1.4. Synthetic Procedure for Amines (**10**–**12**)

To the solution of **7**–**9** (7 mmol, 1 eq) in DCM (15 mL), TFA (21 mmol, 3 eq) was added and solution was mixed at room temperature for 3 h. Next, DCM was evaporated under reduced pressure. Obtained oils were dissolved in small amount of water (20 mL), and then 25% ammonium hydroxide was added dropwise, until pH = 10. The aqueous layer was extracted with DCM (3 × 20 mL), then dried over anhydrous Na_2_SO_4_, and concentrated to give **10**–**12** compounds as oils. Obtained amines **10**–**12** were used as substrates for the next reactions.

1-(3-(Trifluoromethyl)phenyl)piperazine (**10**).

Light oil. Yield: 97% (1.52 g); UPLC (purity = 97.28%): t_R_ = 3.75 min. C_11_H_13_F_3_N_2_ (230.10). LC-MS (ESI): *m*/*z* calcd for C_11_H_13_F_3_N_2_ [M+H]^+^ 231.10, found 231.1.

1-(3-(Trifluoromethoxy)phenyl)piperazine (**11**).

Light oil. Yield: 96% (1.60 g); UPLC (purity = 96.54%): t_R_ = 3.99 min. C_11_H_13_F_3_N_2_O (246.10). LC-MS (ESI): *m*/*z* calcd for C_11_H_13_F_3_N_2_O [M+H]^+^ 247.10, found 247.1.

1-(3-((Trifluoromethyl)thio)phenyl)piperazine (**12**).

Light oil. Yield: 95% (1.69 g); UPLC (purity = 97.13%): t_R_ = 3.69 min. C_11_H_13_F_3_N_2_S (262.08). LC-MS (ESI): *m*/*z* calcd for C_11_H_13_F_3_N_2_S [M+H]^+^ 263.08, found 263.1.

#### 3.1.5. Synthetic Procedure for Phtalimide Derivatives (**13**–**18**)

The amines obtained in previous step (**10**–**12**, 5.2 mmol, 1 eq) were dissolved in acetone. Then, K_2_CO_3_ (2.87 g, 20.8 mmol, 4 eq) and KI (1.73 g, 10.4 mmol, 2 eq) were added (as solids). Then, the reaction mixture was heated to 60 °C, and *N*-(3-bromopropyl)- phtalimide (1.39 g, 5.2 mmol, 1 eq) or *N*-(3-bromoethyl)-phtalimide (1.23 g, 5.2 mmol, 1 eq), dissolved in acetone, was added dropwise. Next, the reaction mixture was refluxed for 20 h. Afterwards, the acetone was evaporated and obtained compounds **13**–**18** were purified by column chromatography using the developing systems S_2_ and S_3_ and used as a substrates for the next reaction.

2-(2-(4-(3-(Trifluoromethyl)phenyl)piperazin-1-yl)ethyl)isoindoline-1,3-dione (**13**).

Light oil. Yield: 65% (1.34 g); TLC: R_f_ = 0.67 (S_2_); UPLC (purity = 98.25%): t_R_ = 6.45 min. C_21_H_20_F_3_N_3_O_2_ (403.15). LC-MS (ESI): *m*/*z* calcd for C_21_H_20_F_3_N_3_O_2_ [M+H]^+^ 404.15, found 404.3.

2-(3-(4-(3-(Trifluoromethyl)phenyl)piperazin-1-yl)propyl)isoindoline-1,3-dione (**14**).

Light oil. Yield: 77% (1.67 g); TLC: R_f_ = 0.36 (S_3_); UPLC (purity = 99.78%): t_R_ = 6.38 min. C_22_H_22_F_3_N_3_O_2_ (417.17). LC-MS (ESI): *m*/*z* calcd for C_22_H_22_F_3_N_3_O_2_ [M+H]^+^ 418.17, found 418.2.

2-(2-(4-(3-(Trifluoromethoxy)phenyl)piperazin-1-yl)ethyl)isoindoline-1,3-dione (**15**).

Light oil. Yield: 62% (1.34 g); TLC: R_f_ = 0.40 (S_2_); UPLC (purity = 99.45%): t_R_ = 6.59 min. C_21_H_20_F_3_N_3_O_3_ (419.15). LC-MS (ESI): *m*/*z* calcd for C_21_H_20_F_3_N_3_O_3_ [M+H]^+^ 420.15, found 420.2.

2-(3-(4-(3-(Trifluoromethoxy)phenyl)piperazin-1-yl)propyl)isoindoline-1,3-dione (**16**).

Light oil. Yield: 75% (1.65 g); TLC: R_f_ = 0.40 (S_3_); UPLC (purity = 97.87%): t_R_ = 6.44 min. C_22_H_22_F_3_N_3_O_3_ (433.16). LC-MS (ESI): *m*/*z* calcd for C_22_H_22_F_3_N_3_O_3_ [M+H]^+^ 434.16, found 434.1.

2-(2-(4-(3-((Trifluoromethyl)thio)phenyl)piperazin-1-yl)ethyl)isoindoline-1,3-dione (**17**).

Light oil. Yield: 76% (1.65 g); TLC: R_f_ = 0.43 (S_2_); UPLC (purity = 96.23%): t_R_ = 6.51 min. C_21_H_20_F_3_N_3_O_2_S (435.12). LC-MS (ESI): *m*/*z* calcd for C_21_H_20_F_3_N_3_O_2_S [M+H]^+^ 436.13, found 436.2.

2-(3-(4-(3-((Trifluoromethyl)thio)phenyl)piperazin-1-yl)propyl)isoindoline-1,3-dione (**18**).

Light oil. Yield: 59% (1.35 g); TLC: R_f_ = 0.36 (S_3_); UPLC (purity = 97.65%): t_R_ = 6.34 min. C_22_H_22_F_3_N_3_O_2_S (449.49). LC-MS (ESI): *m*/*z* calcd for C_22_H_22_F_3_N_3_O_2_S [M+H]^+^ 450.14, found 450.2.

#### 3.1.6. Synthetic Procedure of Aminolysis—Synthesis of Amines (**19**–**24**)

Next, compounds **13**–**18** (2.5 mmol, 1 eq) were dissolved in anhydrous THF, and a 2M solution of methylamine in THF (2.5 mmol, 1 eq) was added. The mixture was refluxed for the next 24 h. Afterwards, aqueous solution of NaOH (2.5 mmol, 1 eq) was added by drops, and mixture was refluxed at 100 °C for 3 h. Then, organic solvent was evaporated. The aqueous layer was extracted with DCM (3 × 20 mL), then dried over anhydrous Na_2_SO_4_ and concentrated to give compounds **19**–**24** as oils.

2-(4-(3-(Trifluoromethyl)phenyl)piperazin-1-yl)ethan-1-amine (**19**).

Light oil. Yield: 70% (0.4 g); UPLC (purity = 84.34%): t_R_ = 4.16 min. C_13_H_18_F_3_N_3_ (273.30). LC-MS (ESI): *m*/*z* calcd for C_13_H_18_F_3_N_3_ [M+H]^+^ 274.15, found 274.2. ^1^H NMR (500 MHz, CDCl_3_) δ 2.06 (br s, 2 H), 2.50 (t, *J* = 6.1 Hz, 2 H), 2.59–2.64 (m, 4 H), 2.84 (t, *J* = 6.1 Hz, 2 H), 3.20–3.24 (m, 4 H), 7.02–7.07 (m, 2 H), 7.09 (s, 1 H), 7.32 (t, *J* = 7.8 Hz, 1 H).

3-(4-(3-(Trifluoromethyl)phenyl)piperazin-1-yl)propan-1-amine (**20**).

Light oil. Yield: 67% (0.46 g); UPLC (purity = 96.32%): t_R_ = 3.95 min. C_14_H_20_F_3_N_3_ (287.33). LC-MS (ESI): *m*/*z* calcd for C_14_H_20_F_3_N_3_ [M+H]^+^ 288.16, found 288.2. ^1^H NMR (500 MHz, CDCl_3_) δ 1.74 (quin, *J* = 6.7 Hz, 2 H), 2.51 (t, *J* = 6.9 Hz, 2 H), 2.58–2.64 (m, 6 H), 2.88 (t, *J* = 6.5 Hz, 2 H), 3.21–3.25 (m, 1 H), 3.21–3.24 (m, 3 H), 7.01–7.07 (m, 2 H), 7.08 (s, 1 H), 7.32 (t, *J* = 8.0 Hz, 1 H).

2-(4-(3-(Trifluoromethoxy)phenyl)piperazin-1-yl)ethan-1-amine (**21**).

Light oil. Yield: 67% (0.43 g); UPLC (purity = 89.46%): t_R_ = 4.46 min. C_13_H_18_F_3_N_3_O (289.30). LC-MS (ESI): *m*/*z* calcd for C_13_H_18_F_3_N_3_O [M+H]^+^ 290.14, found 290.2. ^1^H NMR (500 MHz, CDCl_3_) δ 1.72 (br s, 2 H), 2.47 (t, *J* = 6.2 Hz, 2 H), 2.58–2.61 (m, 4 H), 2.82 (t, *J* = 6.1 Hz, 2 H), 3.18–3.22 (m, 4 H), 6.67 (dd, *J* = 8.1, 0.9 Hz, 1 H), 6.69 (s, 1 H), 6.80 (dd, *J* = 8.4, 2.1 Hz, 1 H), 7.22 (t, *J* = 7.8 Hz, 1 H).

3-(4-(3-(Trifluoromethoxy)phenyl)piperazin-1-yl)propan-1-amine (**22**).

Light oil. Yield: 63% (0.42 g); UPLC (purity = 88.32): t_R_ = 4.19 min. C_14_H_20_F_3_N_3_O (303.33). LC-MS (ESI): *m*/*z* calcd for C_14_H_20_F_3_N_3_O [M+H]^+^ 304.16, found 304.2. ^1^H NMR (500 MHz, CDCl_3_) δ 1.67 (quin, *J* = 7.1 Hz, 2 H), 1.94 (s, 2 H), 2.43–2.47 (m, 2 H), 2.56–2.60 (m, 4 H), 2.77 (t, *J* = 6.8 Hz, 2 H), 3.17–3.22 (m, 4 H), 6.64–6.68 (m, 1 H), 6.69 (br s, 1 H), 6.80 (dd, *J* = 8.2, 2.7 Hz, 1 H), 7.22 (t, *J* = 7.8 Hz, 1 H).

2-(4-(3-((Trifluoromethyl)thio)phenyl)piperazin-1-yl)ethan-1-amine (**23**).

Light oil. Yield: 65% (0.45 g); UPLC (purity = 90.24): t_R_ = 4.76 min. C_13_H_18_F_3_N_3_S (305.36). LC-MS (ESI): *m*/*z* calcd for C_13_H_18_F_3_N_3_S [M+H]^+^ 306.12, found 306.2. ^1^H NMR (500 MHz, CDCl_3_) δ 1.78 (br s, 2 H), 2.43–2.48 (m, 2 H), 2.57–2.63 (m, 4 H), 2.79 (t, *J* = 6.7 Hz, 2 H), 3.19–3.24 (m, 4 H), 6.99 (d, *J* = 8.3 Hz, 1 H), 7.10 (d, *J* = 7.6 Hz, 1 H), 7.14 (t, *J* = 1.9 Hz, 1 H), 7.23–7.29 (m, 1 H).

3-(4-(3-((Trifluoromethyl)thio)phenyl)piperazin-1-yl)propan-1-amine (**24**).

Light oil. Yield: 72% (0.52 g); UPLC (purity = 89.82): t_R_ = 4.50 min. C_14_H_20_F_3_N_3_S (319.33). LC-MS (ESI): *m*/*z* calcd for C_14_H_20_F_3_N_3_S [M+H]^+^ 320.14, found 320.1. ^1^H NMR (500 MHz, CDCl_3_) δ 1.67 (quin, *J* = 7.1 Hz, 2 H), 1.75 (br s, 2 H), 2.44–2.47 (m, 2 H), 2.58–2.61 (m, 4 H), 2.78 (t, *J* = 6.7 Hz, 2 H), 3.20–3.23 (m, 4 H), 6.99 (d, *J* = 8.3 Hz, 1 H), 7.09 (d, *J* = 7.6 Hz, 1 H), 7.14 (t, *J* = 1.9 Hz, 1 H), 7.24–7.28 (m, 1 H)

#### 3.1.7. Synthetic Procedure for Fusion Reaction (**25**–**31**)

In the next step, obtained amines **19**–**24** (1.3 mmol, 1 eq) were fused in 180 °C with acids **5** or **6** (1.3 mmol, 1 eq), without solvent. After 1 h, the products were cooled and purified by column chromatography using the following developing system: S_5_. Then, compounds were treated by 2M methanolic hydrochloric acid solution. Hydrochloride salts **25**–**31** were obtained as solids after adding small amount of Et_2_O and fast evaporation under reduced pressure.

3-(Benzo[*b*]thiophen-2-yl)-1-(2-(4-(3-(trifluoromethyl)phenyl)piperazin-1-yl)ethyl)pyrrolidine-2,5-dione hydrochloride (**25**).

White solid. Yield: 90% (0.61 g); mp. 193.5–194.5 °C; UPLC (purity = 99.26%): t_R_ = 6.46 min. C_25_H_25_ClF_3_N_3_O_2_S (524.00). LC-MS (ESI): *m*/*z* calcd for C_25_H_24_F_3_N_3_O_2_S [M+H]^+^ 488.16, found 488.2. UPLC/HRMS (purity = 99.58%): *t*_R_ = 6.06 min. HRMS (ESI-QTOF): *m*/*z* calcd for C_25_H_24_F_3_N_3_O_2_S [M+H]^+^ 488.1585, found 488.1652. ^1^H NMR (500 MHz, CDCl_3_) δ 2.56–2.73 (m, 6 H), 3.01 (dd, *J* = 18.3, 4.6 Hz, 1 H), 3.10 (t, *J* = 5.0 Hz, 4 H), 3.28 (dd, *J* = 18.3, 9.5 Hz, 1 H), 3.75 (td, *J* = 6.2, 1.4 Hz, 2 H), 4.34 (dd, *J* = 9.3, 4.7 Hz, 1 H), 6.98 (dd, *J* = 8.4, 2.1 Hz, 1 H), 7.04 (d, *J* = 4.9 Hz, 2 H), 7.24–7.33 (m, 4 H), 7.65 (d, *J* = 7.3 Hz, 1 H), 7.72 (d, *J* = 7.5 Hz, 1 H). ^1^H NMR (500 MHz, DMSO-*d*_6_) δ 3.03 (dd, *J* = 17.8, 5.4 Hz, 1 H), 3.10–3.25 (m, 4 H), 3.29 (d, *J* = 9.2 Hz, 2 H), 3.57–4.04 (m, 7 H), 4.63 (dd, *J* = 8.4, 5.9 Hz, 1 H), 7.04 (s, 1 H), 7.08–7.16 (m, 2 H), 7.24 (s, 1 H), 7.27–7.35 (m, 2 H), 7.43 (s, 1 H), 7.75 (d, *J* = 7.4 Hz, 1 H), 7.90 (d, *J* = 7.7 Hz, 1 H), 10.98 (br s, 1 H). ^13^C NMR (126 MHz, CDCl_3_) δ 36.3, 37.0, 41.9, 48.7, 52.8, 54.5, 112.1, 112.1, 115.8, 115.8, 118.8, 124.4 (q, *J* = 272.6 Hz), 122.3, 122.6, 123.6, 124.7, 129.5, 131.4 (q, *J* = 31.0 Hz), 139.3, 139.4, 139.5, 151.5, 175.3, 176.1. ^19^F NMR (471 MHz, DMSO-*d*_6_) δ −60.93 (s, 3 F).

3-(Benzo[*b*]thiophen-2-yl)-1-(3-(4-(3-(trifluoromethyl)phenyl)piperazin-1-yl)propyl)pyrrolidine-2,5-dione hydrochloride (**26**).

White solid. Yield: 90% (0.61 g); mp. 175.0–176.5 °C; UPLC (purity = 97.68%): t_R_ = 6.80 min. C_26_H_27_ClF_3_N_3_O_2_S (538.03). LC-MS (ESI): *m*/*z* calcd for C_26_H_26_F_3_N_3_O_2_S [M+H]^+^ 502.17, found 502.6. UPLC/HRMS (purity = 97.95%): *t*_R_ = 6.00 min. HRMS (ESI-QTOF): *m*/*z* calcd for C_26_H_26_F_3_N_3_O_2_S [M+H]^+^ 502.1791, found 502.1846. ^1^H NMR (500 MHz, CDCl_3_) δ 1.86 (quin, *J* = 7.1 Hz, 2 H), 2.46 (t, *J* = 7.0 Hz, 2 H), 2.55–2.58 (m, 4 H), 3.04 (dd, *J* = 18.3, 5.2 Hz, 1 H), 3.18 (dd, *J* = 6.2, 3.6 Hz, 4 H), 3.30 (dd, *J* = 18.3, 9.5 Hz, 1 H), 3.70 (t, *J* = 7.0 Hz, 2 H), 4.35 (ddd, *J* = 9.4, 4.9, 0.9 Hz, 1 H), 7.00 (d, *J* = 8.2 Hz, 1 H), 7.06 (s, 1 H), 7.08 (s, 2 H), 7.27 (s, 1 H), 7.30–7.38 (m, 2 H), 7.72 (d, *J* = 7.6 Hz, 1 H), 7.79 (d, *J* = 7.4 Hz, 1 H). ^1^H NMR (500 MHz, DMSO-*d*_6_) δ 1.98 (quin, *J* = 7.4 Hz, 2 H), 3.00 (dd, *J* = 17.8, 5.4 Hz, 1 H), 3.04–3.21 (m, 6 H), 3.29 (dd, *J* = 17.9, 9.3 Hz, 1 H), 3.44–3.51 (m, 4 H), 3.91 (d, *J* = 12.9 Hz, 2 H), 4.57 (dd, *J* = 9.2, 5.4 Hz, 1 H), 7.12 (d, *J* = 7.4 Hz, 1 H), 7.21–7.27 (m, 2 H), 7.28–7.36 (m, 2 H), 7.41–7.45 (m, 2 H), 7.76 (d, *J* = 7.4 Hz, 1 H), 7.90 (d, *J* = 7.7 Hz, 1 H), 10.86 (br s, 1 H). ^13^C NMR (126 MHz, CDCl_3_) δ 24.6, 36.8, 37.9, 41.9, 48.7, 53.0, 53.5, 55.9, 112.2, 115.8, 118.7, 124.4 (q, *J* = 272.6 Hz), 122.3, 122.6, 123.6, 124.8, 129.6, 131.4 (q, *J* = 32.0 Hz), 139.2, 139.3, 139.4, 151.4, 175.3, 176.1. ^19^F NMR (471 MHz, DMSO-*d*_6_) δ −60.94 (s, 3 F).

3-(Benzo[*b*]thiophen-2-yl)-1-(2-(4-(3-(trifluoromethoxy)phenyl)piperazin-1-yl)ethyl)pyrrolidine-2,5-dione hydrochloride (**27**).

Light yellow solid. Yield: 89% (0.61 g); mp. 186.0–187.5 °C; UPLC (purity = 97.51%): t_R_ = 7.01 min. C_25_H_25_ClF_3_N_3_O_3_S (540.00). LC-MS (ESI): *m*/*z* calcd for C_25_H_24_F_3_N_3_O_3_S [M+H]^+^ 504.15, found 504.2. UPLC/HRMS (purity = 97.65%): *t*_R_ = 6.23 min. HRMS (ESI-QTOF): *m*/*z* calcd for C_25_H_24_F_3_N_3_O_3_S [M+H]^+^ 504.1524, found 504.1572. ^1^H NMR (500 MHz, CDCl_3_) δ 2.60–2.66 (m, 6 H), 3.01 (dd, *J* = 18.3, 4.9 Hz, 1 H), 3.06–3.11 (m, 4 H), 3.28 (dd, *J* = 18.3, 9.5 Hz, 1 H), 3.75 (t, *J* = 6.2 Hz, 2 H), 4.34 (dd, *J* = 9.2, 4.3 Hz, 1 H), 6.64 (s, 1 H), 6.66 (d, *J* = 8.3 Hz, 1 H), 6.74 (dd, *J* = 8.3, 2.3 Hz, 1 H), 7.20 (t, *J* = 8.3 Hz, 1 H), 7.25 (s, 1 H), 7.29 (ddd, *J* = 7.3, 5.4, 1.6 Hz, 2 H), 7.65 (d, *J* = 7.1 Hz, 1 H), 7.73 (d, *J* = 7.4 Hz, 1 H). ^1^H NMR (500 MHz, DMSO-*d*_6_) δ 3.03 (dd, *J* = 17.6, 5.6 Hz, 2 H), 3.07–3.27 (m, 6 H), 3.62 (br s, 2 H), 3.82–3.91 (m, 4 H), 4.65 (br s, 1 H), 6.75 (d, *J* = 7.7 Hz, 1 H), 6.93 (br s, 1 H), 6.98 (d, *J* = 8.0 Hz, 1 H), 7.31 (t, *J* = 8.3 Hz, 3 H), 7.43 (s, 1 H), 7.75 (d, *J* = 7.4 Hz, 1 H), 7.90 (d, *J* = 7.7 Hz, 1 H), 11.29 (br s, 1 H). ^13^C NMR (126 MHz, CDCl_3_) δ 36.3, 37.0, 41.9, 48.6, 52.8, 54.5, 108.3, 111.2, 113.8, 120.6 (q, *J* = 256.5 Hz), 122.3, 122.6, 123.6, 124.7, 130.0, 139.3, 139.4, 139.6, 150.3, 152.6, 175.3, 176.1. ^19^F NMR (471 MHz, DMSO-*d*_6_) δ −56.39 (s, 3 F).

3-(Benzo[*b*]thiophen-2-yl)-1-(3-(4-(3-(trifluoromethoxy)phenyl)piperazin-1-yl)propyl)pyrrolidine-2,5-dione hydrochloride (**28**).

White solid. Yield: 82% (0.58 g); mp. 162.0–163.0 °C; UPLC (purity > 99%): t_R_ = 7.04 min. C_26_H_27_ClF_3_N_3_O_3_S (554.03). LC-MS (ESI): *m*/*z* calcd for C_26_H_26_F_3_N_3_O_3_S [M+H]^+^ 518.17, found 518.3. UPLC/HRMS (purity = 98.89%): *t*_R_ = 6.10 min. HRMS (ESI-QTOF): *m*/*z* calcd for C_26_H_26_F_3_N_3_O_3_S [M+H]^+^ 518.1761, found 518.1802. ^1^H NMR (500 MHz, CDCl_3_) δ 1.83 (quin, *J* = 7.2 Hz, 2 H), 2.43 (t, *J* = 6.9 Hz, 2 H), 2.53 (t, *J* = 5.0 Hz, 4 H), 3.02 (dd, *J* = 18.2, 5.0 Hz, 1 H), 3.13 (dd, *J* = 6.3, 3.7 Hz, 4 H), 3.28 (dd, *J* = 18.3, 9.5 Hz, 1 H), 3.68 (t, *J* = 7.2 Hz, 2 H), 4.33 (ddd, *J* = 9.5, 5.0, 1.0 Hz, 1 H), 6.65 (s, 1 H), 6.66 (d, *J* = 7.6 Hz, 1 H), 6.73–6.76 (m, 1 H), 7.18–7.22 (m, 1 H), 7.24 (s, 1 H), 7.32 (quind, *J* = 7.4, 7.4, 7.4, 7.4, 1.4 Hz, 2 H), 7.70 (d, *J* = 7.4 Hz, 1 H), 7.77 (dd, *J* = 7.6, 1.0 Hz, 1 H). ^1^H NMR (500 MHz, DMSO-*d*_6_) δ 1.99 (quin, *J* = 7.3 Hz, 2 H), 2.96–3.02 (m, 1 H), 3.02–3.22 (m, 6 H), 3.29 (dd, *J* = 17.8, 9.2 Hz, 1 H), 3.47 (d, *J* = 6.0 Hz, 4 H), 3.85 (d, *J* = 12.9 Hz, 2 H), 4.57 (dd, *J* = 8.9, 5.4 Hz, 1 H), 6.75 (d, *J* = 8.0 Hz, 1 H), 6.91 (br s, 1 H), 6.96 (d, *J* = 7.8 Hz, 1 H), 7.28–7.33 (m, 3 H), 7.41 (s, 1 H), 7.76 (d, *J* = 7.4 Hz, 1 H), 7.89 (d, *J* = 7.7 Hz, 1 H), 11.11 (br s, 1 H). ^13^C NMR (126 MHz, CDCl_3_) δ 24.6, 36.8, 37.9, 41.9, 48.6, 53.0, 55.9, 108.3, 111.2, 113.7, 119.6, 122.3, 122.6, 123.6, 124.8, 130.0, 139.2, 139.3, 139.4, 150.3, 152.6, 175.3, 176.1. ^19^F NMR (471 MHz, DMSO-*d*_6_) δ −56.39 (s, 3 F).

3-(Benzo[*b*]thiophen-2-yl)-1-(2-(4-(3-((trifluoromethyl)thio)phenyl)piperazin-1-yl)ethyl)pyrrolidine-2,5-dione hydrochloride (**29**).

White solid. Yield: 84% (0.60 g); mp. 210.0–211.0 °C; UPLC (purity = 98.35%): t_R_ = 7.32 min. C_25_H_25_ClF_3_N_3_O_2_S_2_ (556.06). LC-MS (ESI): *m*/*z* calcd for C_25_H_24_F_3_N_3_O_2_S_2_ [M+H]^+^ 520.13, found 520.2. UPLC/HRMS (purity = 98.78%): *t*_R_ = 6.45 min. HRMS (ESI-QTOF): *m*/*z* calcd for C_25_H_24_F_3_N_3_O_2_S_2_ [M+H]^+^ 520.1296, found 520.1393. ^1^H NMR (500 MHz, CDCl_3_) δ 2.60–2.66 (m, 6 H), 3.01 (dd, *J* = 18.3, 4.6 Hz, 1 H), 3.08–3.11 (m, 4 H), 3.28 (dd, *J* = 18.3, 9.5 Hz, 1 H), 3.73–3.76 (m, 2 H), 4.34 (dd, *J* = 9.2, 4.6 Hz, 1 H), 6.93 (dd, *J* = 8.3, 1.7 Hz, 1 H), 7.07–7.09 (m, 2 H), 7.22–7.25 (m, 1 H), 7.25 (s, 1 H), 7.28 (ddd, *J* = 7.4, 5.4, 1.6 Hz, 2 H), 7.65 (d, *J* = 7.3 Hz, 1 H), 7.72 (d, *J* = 7.3 Hz, 1 H). ^1^H NMR (500 MHz, DMSO-*d*_6_) δ 3.03 (dd, *J* = 17.6, 5.6 Hz, 1 H), 3.09–3.19 (m, 2 H), 3.22–3.28 (m, 2 H), 3.28–3.35 (m, 1 H), 3.34–3.41 (m, 2 H), 3.63 (br s, 2 H), 3.83–3.92 (m, 4 H), 4.68 (dd, *J* = 8.9, 6.0 Hz, 1 H), 7.11 (d, *J* = 7.7 Hz, 1 H), 7.19 (dd, *J* = 8.3, 2.0 Hz, 1 H), 7.27–7.38 (m, 4 H), 7.43 (s, 1 H), 7.75 (d, *J* = 7.2 Hz, 1 H), 7.89 (d, *J* = 7.4 Hz, 1 H), 11.54 (br s, 1 H). ^13^C NMR (126 MHz, CDCl_3_) δ 36.3, 37.0, 41.9, 48.7, 52.8, 54.5, 118.0, 122.3, 122.6, 123.1, 123.6, 124.7, 124.8, 125.0, 129.9 (q, *J* = 307.8 Hz), 126.8, 130.0, 139.3, 139.4, 139.6, 152.0, 175.3, 176.1. ^19^F NMR (471 MHz, DMSO-*d*_6_) δ −41.73 (s, 3 F).

3-(Benzo[*b*]thiophen-2-yl)-1-(3-(4-(3-((trifluoromethyl)thio)phenyl)piperazin-1-yl)propyl)pyrrolidine-2,5-dione hydrochloride (**30**).

White solid. Yield: 84% (0.62 g); mp. 175.5–176.5 °C; UPLC (purity = 98.80%): t_R_ = 7.22 min. C_26_H_27_ClF_3_N_3_O_2_S_2_ (570.09). LC-MS (ESI): *m*/*z* calcd for C_26_H_26_F_3_N_3_O_2_S_2_ [M+H]^+^ 534.15, found 534.2. UPLC/HRMS (purity = 97.81%): *t*_R_ = 6.23 min. HRMS (ESI-QTOF): *m*/*z* calcd for C_26_H_26_F_3_N_3_O_2_S_2_ [M+H]^+^ 534.1492, found 534.1556. ^1^H NMR (500 MHz, CDCl_3_) δ 1.84 (quin, *J* = 7.1 Hz, 2 H), 2.44 (t, *J* = 6.9 Hz, 2 H), 2.54 (t, *J* = 5.0 Hz, 4 H), 3.02 (dd, *J* = 18.2, 5.0 Hz, 1 H), 3.14 (dd, *J* = 6.0, 3.4 Hz, 4 H), 3.28 (dd, *J* = 18.3, 9.5 Hz, 1 H), 3.68 (t, *J* = 7.2 Hz, 2 H), 4.33 (ddd, *J* = 9.5, 5.0, 1.0 Hz, 1 H), 6.93 (d, *J* = 8.1 Hz, 1 H), 7.08 (d, *J* = 7.7 Hz, 1 H), 7.10 (d, *J* = 1.7 Hz, 1 H), 7.22–7.27 (m, 2 H), 7.32 (quind, *J* = 7.3, 7.3, 7.3, 7.3, 1.6 Hz, 2 H), 7.70 (dd, *J* = 7.0, 1.6 Hz, 1 H), 7.77 (d, *J* = 7.4 Hz, 1 H). ^1^H NMR (500 MHz, DMSO-*d*_6_) δ 2.00 (quin, *J* = 7.4 Hz, 2 H), 2.99 (dd, *J* = 17.8, 5.4 Hz, 1 H), 3.04–3.15 (m, 4 H), 3.16–3.24 (m, 2 H), 3.26–3.31 (m, 1 H), 3.43–3.52 (m, 4 H), 3.85 (d, *J* = 12.9 Hz, 2 H), 4.58 (dd, *J* = 8.6, 5.4 Hz, 1 H), 7.11 (d, *J* = 7.4 Hz, 1 H), 7.18 (dd, *J* = 8.4, 2.1 Hz, 1 H), 7.25 (s, 1 H), 7.27–7.34 (m, 2 H), 7.35 (d, *J* = 8.0 Hz, 1 H), 7.41 (s, 1 H), 7.76 (d, *J* = 7.4 Hz, 1 H), 7.89 (d, *J* = 7.7 Hz, 1 H), 11.34 (br s, 1 H). ^13^C NMR (126 MHz, CDCl_3_) δ 24.6, 36.8, 37.9, 41.9, 48.7, 53.0, 55.9, 117.9, 122.4, 122.6, 123.2, 123.7, 124.8, 125.0, 129.8 (q, *J* = 307.8 Hz), 126.8, 130.0, 139.2, 139.3, 139.4, 151.9, 175.3, 176.1. ^19^F NMR (471 MHz, DMSO-*d*_6_) δ −41.73 (s, 3 F).

3-(Benzofuran-2-yl)-1-(3-(4-(3-(trifluoromethoxy)phenyl)piperazin-1-yl)propyl)pyrrolidine-2,5-dione hydrochloride (**31**).

White solid. Yield: 87% (0.60 g); mp. 100.0–102.0 °C; UPLC (purity > 99%): t_R_ = 7.34 min. C_26_H_27_ClF_3_N_3_O_4_ (537.96). LC-MS (ESI): *m*/*z* calcd for C_26_H_26_F_3_N_3_O_4_ [M+H]^+^ 502.19, found 502.2. UPLC/HRMS (purity = 98.60%): *t*_R_ = 5.98 min. HRMS (ESI-QTOF): *m*/*z* calcd for C_26_H_26_F_3_N_3_O_4_ [M+H]^+^ 502.1909, found 502.1933. ^1^H NMR (500 MHz, CDCl_3_) δ 1.85 (quin, *J* = 7.1 Hz, 2 H), 2.44 (t, *J* = 6.9 Hz, 2 H), 2.54–2.56 (m, 4 H), 3.09 (d, *J* = 5.3 Hz, 1 H), 3.13 (d, *J* = 9.3 Hz, 1 H), 3.15–3.18 (m, 4 H), 3.69 (t, *J* = 7.2 Hz, 2 H), 4.23 (dd, *J* = 9.1, 5.1 Hz, 1 H), 6.65–6.69 (m, 2 H), 6.72 (t, *J* = 0.8 Hz, 1 H), 6.77 (dt, *J* = 9.1, 1.3 Hz, 1 H), 7.21 (td, *J* = 7.4, 1.1 Hz, 2 H), 7.25–7.28 (m, 1 H), 7.39 (dd, *J* = 8.2, 0.9 Hz, 1 H), 7.50–7.54 (m, 1 H). ^1^H NMR (500 MHz, DMSO-*d*_6_) δ 2.02 (quin, *J* = 7.4 Hz, 2 H), 2.97 (dd, *J* = 17.8, 5.6 Hz, 1 H), 3.04–3.15 (m, 4 H), 3.16–3.24 (m, 2 H), 3.26–3.31 (m, 1 H), 3.43–3.52 (m, 4 H), 3.85 (d, *J* = 12.9 Hz, 2 H), 4.58 (dd, *J* = 8.6, 5.4 Hz, 1 H), 7.10–7.20 (m, 2 H), 7.25 (s, 1 H), 7.27–7.40 (m, 4 H), 7.75 (d, *J* = 7.4 Hz, 1 H), 7.87 (d, *J* = 7.7 Hz, 1 H), 11.36 (br s, 1 H). ^13^C NMR (126 MHz, CDCl_3_) δ 24.7, 34.1, 38.0, 40.5, 48.6, 53.0, 55.8, 105.5, 108.3, 111.2, 111.3, 113.7, 121.2, 121.6, 123.2, 124.7, 128.0, 130.1, 150.3, 151.4, 152.6, 155.0, 174.9, 175.5. ^19^F NMR (471 MHz, DMSO-*d*_6_) δ −56.39 (s, 3 F).

#### 3.1.8. Synthetic Procedure for Final Hydrochloride Derivatives (**32**–**33**)

Acid **5** (1 mmol, 1 eq) was fused with commercially available 2-morpholinoethan-1-amine and 2-morpholinopropan-1-amine (1 mmol, 1 eq). After 1 h, the products were cooled and purified by column chromatography using the following developing system: S_5._ Then, compounds were treated by 2M methanolic hydrochloric acid solution. Hydrochloride salts **32**–**33** were obtained as solids after adding small amount of Et_2_O and fast evaporation under reduced pressure.

3-(Benzo[*b*]thiophen-2-yl)-1-(2-morpholinoethyl)pyrrolidine-2,5-dione hydrochloride (**32**). Light yellow solid. Yield: 85% (0.32 g); mp. 100.0–101.0 °C; UPLC (purity > 99%): t_R_ = 5.02 min. C_18_H_21_ClN_2_O_3_S (380.89) LC-MS (ESI): *m*/*z* calcd for C_18_H_20_N_2_O_3_S [M+H]^+^ 345.12 found 345.2. UPLC/HRMS (purity > 99.99%): *t*_R_ = 4.45 min. HRMS (ESI-QTOF): *m*/*z* calcd for C_18_H_20_N_2_O_3_S [M+H]^+^ 345.1228, found 345.1232. ^1^H NMR (500 MHz, CDCl_3_) δ 2.34–2.41 (m, 6 H), 3.01 (dd, *J* = 18.2, 5.0 Hz, 1 H), 3.27 (dd, *J* = 18.2, 9.5 Hz, 1 H), 3.64–3.67 (m, 6 H), 4.33 (ddd, *J* = 9.5, 5.0, 0.9 Hz, 1 H), 7.24 (s, 1 H), 7.30–7.36 (m, 2 H), 7.71 (dd, *J* = 7.2, 1.2 Hz, 1 H), 7.76 (dd, *J* = 7.7, 1.8 Hz, 1 H). ^1^H NMR (500 MHz, DMSO-*d*_6_) δ 2.30–2.45 (m, 6 H), 3.00 (dd, *J* = 18.4, 5.02 Hz, 1 H), 3.27 (dd, *J* = 18.4, 9.6 Hz, 1 H), 3.62–3.69 (m, 6 H), 4.35 (ddd, *J* = 9.6, 5.2, 0.9 Hz, 1 H), 7.26 (s, 1 H), 7.29–7.36 (m, 2 H), 7.71 (dd, *J* = 7.2, 1.4 Hz, 1 H), 7.76–7.79 (m, 1 H). ^13^C NMR (126 MHz, CDCl_3_) δ 36.9, 37.8, 41.9, 53.7, 56.3, 67.1, 122.4, 122.6, 123.7, 124.8, 124.8, 139.3, 139.4, 139.4, 175.2, 176.0.

3-(Benzo[*b*]thiophen-2-yl)-1-(3-morpholinopropyl)pyrrolidine-2,5-dione hydrochloride (**33**).

Light yellow solid. Yield: 84% (0.33 g); mp. 104.1–105.5 °C; UPLC (purity = 98.36%): t_R_ = 4.90 min. C_19_H_23_ClN_2_O_3_S (394.91). LC-MS (ESI): *m*/*z* calcd for C_19_H_22_N_2_O_3_S [M+H]^+^ 359.14, found 359.2. UPLC/HRMS (purity = 98.60%): *t*_R_ = 4.36 min. HRMS (ESI-QTOF): *m*/*z* calcd for C_19_H_22_N_2_O_3_S [M+H]^+^ 359.1385, found 359.1392. ^1^H NMR (500 MHz, CDCl_3_) δ 1.79 (quin, *J* = 7.1 Hz, 2 H), 2.34–2.38 (m, 4 H), 3.01 (dd, *J* = 18.2, 5.0 Hz, 1 H), 3.27 (dd, *J* = 18.2, 9.5 Hz, 1 H), 3.62–3.68 (m, 8 H), 4.33 (ddd, *J* = 9.5, 5.0, 0.9 Hz, 1 H), 7.24 (s, 1 H), 7.29–7.36 (m, 2 H), 7.71 (dd, *J* = 7.2, 1.4 Hz, 1 H), 7.77 (d, *J* = 7.7 Hz, 1 H). ^1^H NMR (500 MHz, DMSO-*d*_6_) δ 1.80 (quin, *J* = 7.2 Hz, 2 H), 2.32–2.37 (m, 4 H), 3.02 (dd, *J* = 18.3, 5.2 Hz, 1 H), 3.25 (dd, *J* = 18.4, 9.6 Hz, 1 H), 3.60–3.70 (m, 8 H), 4.35 (ddd, *J* = 9.4, 5.2, 0.9 Hz, 1 H), 7.23 (s, 1 H), 7.30–7.39 (m, 2 H), 7.71 (dd, *J* = 7.4, 1.6 Hz, 1 H), 7.78 (dd, *J* = 7.7, 1.8 Hz, 1 H). ^13^C NMR (126 MHz, CDCl_3_) δ 24.4, 36.9, 37.8, 41.9, 53.7, 56.3, 67.1, 122.4, 122.6, 123.7, 124.8, 124.8, 139.3, 139.4, 139.4, 175.2, 176.0.

### 3.2. In Vivo Studies

#### 3.2.1. Animals

Male CD-1 mice weighing 20–26 g provided by an accredited animal facility (Jagiellonian University Medical College, Krakow, Poland) were used in the in vivo experiment. The animals were housed in an environmentally controlled room (temperature of 22 ± 2 °C, humidity 55 ± 10%) under 12 h light/dark cycle (light on at 7:00 a.m. and off at 7:00 p.m.) and had free access to food (standard laboratory pellets) and water. The experimental groups consisted of 4–6 mice (anticonvulsant and neurotoxic studies) or 8–10 animals (antinociceptive studies) [27,40]. The experiments were performed between 8 a.m. and 3 p.m. For the experiments, the animals were selected in a random way and trained observers performed all measurements. Care was taken to minimize animal suffering and reduce the number of animals used (3R policy). The experimental protocol was approved by the First Local Ethics Committee on Animal Testing at the Jagiellonian University in Kraków (No 159/2018, 287A/287B/2019, 288/2019, 361A/2019).

#### 3.2.2. Drug Administration

For the in vivo studies, the investigated compounds were suspended in a 1% aqueous solution of Tween 80 and administered intraperitoneally (i.p.) at a volume of 0.1 mL per 10 g body weight 30 min prior to the test. Control animals were administered an equivalent volume of vehicle (1% Tween 80) via the same route as the test compound. Pentylenetetrazole (Sigma-Aldrich, St. Louis, MO, USA) was dissolved in physiological saline. To prepare a 2.5% formalin solution, formaldehyde (POCh, Gliwice, Poland) was dissolved in distilled water. Oxaliplatin (Tocris Bioscience, Bristol, UK) was prepared in a 5% aqueous solution of glucose (Polfa, Kutno, Poland). In the initial neurotoxicity and anticonvulsant evaluations, the animals were administered a constant dose of 100 mg/kg of each compound and experiments were carried out 0.5 h after i.p. injection, as previously described [6,7].

#### 3.2.3. Maximal Electroshock Seizure (MES) Test

In the MES test, an electrical stimulus of sufficient intensity (25 mA, 500 V, 50 Hz, 0.2 s) was delivered via auricular electrodes by the electroshock generator (Rodent Shocker, Type 221, Hugo Sachs, March, Germany) to induce maximal seizures. The endpoint was the tonic extension of the hind limbs. Mice not displaying hind-limb tonic extension were considered to be protected from seizures [41].

#### 3.2.4. The Six Herz (6 Hz) Electrical Stimulation Seizure Test

In the 6 Hz test, psychomotor seizures were induced via corneal stimulation (6 Hz, 32 mA and 44 mA, 0.2 ms rectangular pulse width, 3 s duration) using a constant-current device (ECT Unit 57800, Ugo Basile, Gemonio, Italy). A drop of 1% solution of lidocaine hydrochloride was applied to the mouse corneas before stimulation to provide local anesthesia and ensure optimal current conductivity. After the electrical stimulation, mice were gently released and observed for the presence or absence of seizure activity, being characterized by an initial momentary stun followed immediately by jaw clonus, forelimb clonus, twitching of the vibrissae, and Straub tail [42,43]. Mice resuming normal behavior within 10 s from the stimulation were considered protected [44,45].

#### 3.2.5. Subcutaneous Pentylenetetrazole Seizure (scPTZ) Test

In this test, clonic convulsions were induced by subcutaneous (*s.c.*) administration of pentylenetetrazole (PTZ) at a dose of 100 mg/kg. Subsequently, each mouse was individually placed into plastic cage and observed for the following 30 min. The latency time (in seconds) to the first seizure episode (clonic convulsions lasting for at least 3 s, with accompanying loss of the righting reflex) was measured and compared to the vehicle-treated (1% Tween 80) group [46,47].

#### 3.2.6. Neurotoxicity Screening (NT)—Rotarod Test

In this test, mice were trained to balance on an accelerating rod that rotated at 10 rotations per minute (Rotarod apparatus, May Commat RR0711; rod diameter: 3 cm). During the training session, the animals were placed on a rotating rod for 3 min with an unlimited number of trials. Proper experiment was conducted at least 24 h after the training trial. On the test day, trained mice were intraperitoneally pretreated with the test compound and were evaluated using the rotarod test (at a screening dose of 100 mg/kg just before the MES test). Neurotoxicity was indicated by the inability of the animal to maintain equilibration on the rod for 1 min [48].

#### 3.2.7. Median Effective Dose (ED_50_), Median Toxic Dose (TD_50_), and Protective Index (PI)

The ED_50_ is defined as the dose of a drug protecting 50% of animals against the MES and 6 Hz seizure episodes. The neurotoxic effect was expressed as a TD_50_ value, representing the doses at which the compound resulted in minimal motor impairment in 50% of the animals in the rotarod test. To evaluate the ED_50_ or TD_50_, 3–4 groups of animals were injected with various doses of tested compounds. Each group consisted of six animals. Both ED_50_ and TD_50_ values with 95% confidence limits were calculated by probit analysis [23]. The PI value was calculated as the ratio of TD_50_ to the respective ED_50_ value, as determined in the MES or 6 Hz tests. The PI is considered an index of the margin of safety and tolerability between anticonvulsant doses and doses of the compounds exerting acute adverse effects [49].

#### 3.2.8. Formalin Test

In this test, mice received an intraperitoneal (i.p.) injection of either the test compound or a control solution. After 30 min, a 20 µL injection of a 2.5% formalin solution was administered into the right hind paw. Right after the formalin injection, the animals were individually placed into glass containers and observed for the following 30 min. Time (in seconds) spent licking or biting the injected hind paw in selected intervals, 0–5 min (Phase I) and 15–30 min (Phase II), was measured in each experimental group and was an indicator of nociceptive behavior [27].

#### 3.2.9. Oxaliplatin-Induced Neuropathic Pain Model

Peripheral neuropathy was induced by administering a single dose (10 mg/kg) of oxaliplatin (OXPT) dissolved in a 5% glucose solution. To assess the response to mechanical stimuli, the von Frey test was carried out with application of the electronic von Frey unit (Panlab, Cornellà de Llobregat, Spain). The apparatus contained a single flexible filament, which was used to apply increasing force ranging from 0 to 10 g to the plantar surface of the mouse’s hind paw. The crossing of pain threshold led to paw withdrawal. The mechanical pressure that evoked the nocifensive response was subsequently recorded. In the test, each mouse was placed individually in test compartment with a wire mesh bottom and was allowed to habituate for 30 min. After the habituation period, in order to obtain baseline values of pain sensitivity, each mouse was tested 3 times with at least a 30 s gap between each measurement. The baseline paw withdrawal threshold was measured 3 h (assessment of acute allodynia) and 7 days after oxaliplatin injection. Then, the mice with tactile allodynia were pretreated with the test compound or vehicle. A total of 30 min later, the animals were tested again and mean values of paw withdrawal threshold were obtained for each mouse [40].

#### 3.2.10. Data Analysis

The obtained data are expressed as the mean ± SEM. The data were statistically evaluated using a one-way analysis of variance (ANOVA) or repeated measures ANOVA. For the comparison of only one treatment group with the control group, unpaired *t*-test was used. Differences between groups were considered significant if *p* < 0.05. GraphPad Prism Software v.8, (Boston, MA, USA) was used to analyze the data.

### 3.3. In Vitro Studies

#### 3.3.1. Hepatocytotoxicity and Neurocytotoxicity Assesment

To evaluate selected compounds’ hepatocytotoxicity and neurocytotoxicity, a human hepatocellular carcinoma cell line, HepG2 (ATCC^®^ HB-8065™, Sigma-Aldrich, St. Louis, MO, USA), and a human neuroblastoma cell line, SH-SY5Y (ATCC^®^, CRL-2266™, Sigma-Aldrich, St. Louis, MO, USA), were used. Both cell lines were cultured in standard culture conditions (5% CO_2_, 37 °C, 95% humidity) using Dulbecco′s Modified Eagle′s Medium (DMEM; Gibco, Thermo Scientific, Waltham, MA, USA) supplemented with 10% (vol/vol) fetal bovine serum (FBS; Gibco, Thermo Scientific, Waltham, MA, USA) and antibiotics mixture (Penicillin, Streptomycin, Amphotericin B; Gibco, Thermo Scientific, Waltham, MA, USA). Cells were cultured with **25**–**30**, **32**, or **33** (0.5–100 µM) for 24 h and then an MTT (3-(4,5-dimethylthiazol-2-yl)-2,5diphenyltetrazolium bromide, Sigma-Aldrich, St. Louis, MO, USA) viability assay was performed. The formazan crystals were dissolved in DMSO (Sigma-Aldrich, St. Louis, MO, USA), and the absorbance at 570 nm was measured using a microplate reader (SpectraMax^®^ iD3, Molecular Devices, San Jose, CA, USA). The experiment was performed three times in duplicate. Each bar represents mean (±SEM) percentage of viable cells in comparison to control (defined as 100% and represented untreated cells).

#### 3.3.2. Analysis of Mutagenicity by Ames Microplate Test

The test procedure was provided by Xenometrix and described previously in literature [50,51]. Bacterial strains were grown in exposure medium (24-well plates) for 90 min at 37 °C in the absence (−S9) or presence (+S9) of 4.5% phenobarbital/β-naphthoflavone-induced rat liver S9 and were preincubated in the presence of tested compounds **32** or **33** at concentrations of 100, 200, and 500 µM.

After preincubation, the cultures were transferred to indicator medium by distribution of contents of 24-well culture into 48 wells on a 384-well plate. After 48 h incubation at 37 °C, the number of wells containing bacteria was scored for yellow wells. Positive and negative controls were included in the assay, and all conditions were carred out in triplicate.

To interpret the test results, the following criteria were used: the fold increase in the number of positive wells over the solvent control baseline (FIB) and the dose dependency. The fold increase of revertants relative to the solvent control was determined by dividing the mean number of positive wells at each dose by the solvent control at baseline. The solvent control at baseline was defined as the mean number of positive wells in the solvent control plus one standard deviation (SD). When an increase of more than 2-fold relative to the baseline at more than one dose with a dose response was observed, the sample was classified as positive, whereas when there was no response >2 times the baseline and no dose response, the sample was classified as negative.

#### 3.3.3. Data Analysis

Statistically significant values in hepatocytotoxicity and neurocytotoxicity assessment were compared using the Kruskal–Wallis Test Calculator Followed by post hoc Dunn’s test, *p* < 0.05.

#### 3.3.4. In Vitro Sodium and Calcium Channel Binding Studies

The radioligand binding studies were performed commercially by Cerep (Celle I’Evescault, Poiters, France) using testing procedures described elsewhere, with sodium channels (site 2) and L-type calcium channels (dihydropyridine site) [52,53]. Compound binding was expressed as a percentage of inhibition of the binding of a radioactively labelled ligand. All experiments were performed in duplicate.

### 3.4. In Silico Studies

Lipinski’s rule of five (RO5) parameters, i.e., molecular weight (MW), lipophilicity (log P), number of hydrogen bond donors (NHD), and number of hydrogen bond acceptors (NHA), as well as Veber’s rule, i.e., number of rotatable bonds (NBR) and polar surface area (TPSA), were calculated using SwissAdme website [21]. Central Nervous System Multi-Parameter Optimization (CNS MPO) parameters were determined using Instant JChem 21.4.0 software (ChemAxon, Budapest, Hungary).

## 4. Conclusions and Perspectives

In conclusion, the present findings demonstrate, via several in vivo experiments on CD-1 mice, prominent anticonvulsant activity of new 3-(benzo[b]thiophen-2-yl)pyrrolidine-2,5-dione derivatives in animal models in mice. Five compounds (**28**, **30**, **31**, **32** and **33**) were active in the MES (a model of generalized seizure) as well as in the 6 Hz (a model of partial seizure) tests. Moreover, compound **33** was active in the *sc*PTZ test (a model of absent and myoclonic seizures). The obtained PIs for the new agents were better than those for the ASMs VPA and ETX. Furthermore, compound **33** demonstrated a significant antinociceptive effect in the formalin test, as well as antiallodynic activity in oxaliplatin-induced neuropathy. At active doses, no neurotoxic effect was observed and the obtained TD_50_ value in the rotarod test was >200 mg/kg. Furthermore, the drug-like potential of **33** supports favorable in vitro results, i.e., no hepatocytotoxicity and neurocytotoxicity at a high concentration of 100 μM, as well as lack of mutagenicity at a concentration as high as 500 μM. The in vitro binding studies indicated that the plausible mechanism of action of compound **33** was the influence on the neuronal voltage-sensitive sodium channel (site 2). The present preclinical results indicated that the new tested compounds, especially **33**, demonstrated very promising analgesic, antiallodynic, and broad-spectrum anticonvulsant activity. Compound **33** is proposed to be an interesting candidate for further preclinical development as a therapy for epilepsy and neuropathic pain.

## Figures and Tables

**Table 1:** Drug-like parameters estimated according to Lipinski and Veber’s rules. All parameters were calculated on free amines.

Compd	Lipinski Rule				Veber Rule		CNS MPO
	MW ^a^ ≤ 500	Log P ^b^ ≤ 5	HBD ^c^ ≤ 5	HBA ^d^ ≤ 10	NBR ^e^ ≤ 10	TPSA ^f^ ≤ 140 Å^2^	
**25**	487.54	4.42	0	6	6	72.10	3.35
**26**	501.56	4.74	0	6	7	72.10	3.21
**27**	503.54	4.25	0	7	7	81.33	2.76
**28**	517.56	4.54	0	7	8	81.33	2.83
**29**	519.60	4.82	0	6	7	97.40	2.75
**30**	533.63	5.12	0	6	8	97.40	2.83
**31**	501.50	3.96	0	8	8	66.23	3.59
**32**	344.43	2.25	0	4	4	78.09	6.00
**33**	358.46	2.55	0	4	5	78.09	6.00

^a^ MW: molecular weight; ^b^ Log P lipholicity values; ^c^ HBD: hydrogen bond donors; ^d^ HBA: hydrogen bond acceptors; ^e^ NBR: number of rotatable bonds; ^f^ TPSA: topological polar surface area.

**Table 2:** Quantitative pharmacological parameters; ED_50_, TD_50_, and PI values in mice (i.p.).

Cmpd	TPE (h) ^a^	ED_50_ MES (mg/kg) ^b^	ED_50_ 6 Hz (mg/kg) ^c^	TD_50_ (mg/kg) ^d^	PI (TD_50_/ED_50_) ^e^
**28**	0.5	33.5 (23.3–48.3)	41.1 (27.2–62.0)	>300	>8.9 (MES) >7.3 (6 Hz)
**30**	0.5	41.7 (25.1–69.4)	57.7 (34.0–97.9)	>200	>4.8 (MES) >3.5 (6 Hz)
**31**	0.5	27.3 (22.8–32.7)	36.3 (24.9–53.1)	<200	
**32**	0.5	62.9 (45.7–86.7)	37.7 (17.1–81.5)	>200	>3.2 (MES) >5.3 (6 Hz)
**33**	0.5	24.1 (19.7–29.4)	30.8 (18.2–52.1)	>200	>8.3 (MES) >6.5 (6 Hz)
**ETX**	0.25	-	>200	722.1 (647.0−805.8)	
**LEV** ^f^	1	-	14.8 (11.2−18.4)	>500	>33.8 (6 Hz)
**VPA** ^f^	0.5	216.9 (207.5−226.3)	130.1 (116.3−143.9)	372.9 (356.0−389.8)	1.7 (MES) 2.9 (6 Hz)

Values in parentheses are 95% confidence intervals determined by probit analysis [23]. A dash indicates not tested. ^a^ TPE—time to peak effect, ^b^ ED_50_ (MES—maximal electroshock seizure test), ^c^ ED_50_ (6 Hz, psychomotor seizure test), ^d^ TD_50_ (NT—acute neurological toxicity determined in the rotarod test), ^e^ PI (TD_50_/ED_50_), ^f^ Reference ASMs tested in the same conditions. Data from previous studies are included in ref. [13].

**Table 3:** In vitro binding assays.

Cmpd	Concentration [µM]	Na^+^ Channel (Site 2)	L-Type Ca^2+^ Channel
		% Inhibition of Control Specific Binding	
**32**	100	15.8	28.2
**33**	100	62.2	12.2
Phenytoin ^a^	10	−1.6	6.6
	100	53.9	57.8
Topiramate ^a^	100	-	7.9

The compounds were each evaluated in preparations from the rat cerebral cortex. As inhibitors of specific binding, [^3^H]batrachotoxin was used for the voltage-sensitive sodium channel (site 2) or [^3^H]nitrendipine was used for the L-type Ca^2+^ channel (dihydropyridine site). Results showing an inhibition higher than 50% are considered to represent significant effects of the test compounds; results showing an inhibition between 25% and 50% are indicative of a moderate effect; results showing an inhibition lower than 25% are not considered significant. ^a^ Indicates data from previous studies [13,15].

**Table 4:** Mutagenic properties of **32** and **33** tested using the Ames microplate format (MPF) assay.

Cmpd	Concentration (µM)	FIB			
		*S. typhimurium*—TA98		*S. typhimurium*—TA100	
		−S9	+S9	−S9	+S9
**32**	10	0.33	0.66	0.44	0.83
	50	0.33	0.16	1.77	0.77
	100	0.66	1	1.33	0.55
**33**	10	1	0.66	0.55	1.11
	50	0	0.5	0.77	0.88
	100	0.66	0.66	0.88	1
Positive control *	-	23.8	23.16	10.6	3.6

Fold induction over baseline (FIB) obtained in mutagenic evaluation of **32** and **33** in Ames microplate format (MPF) assay. FIB—fold induction over baseline (baseline = mean zero-dose control + 1 SD); SD—standard deviation. S9—Aroclor 1254-induced S9 fraction. * Positive controls: 2-nitrofluorene (2-NF) at 2 μg/mL (TA98-S9), 4-nitroquinoline-1-oxide (4-NQO) at 0.1 μg/mL (TA100-S9), and 2-aminoanthracene (2-AA) at 1 μg/mL and 2.5 μg/mL (TA98+S9 and TA100+S9, respectively).

## Data Availability

The original contributions presented in the study are included in the article/Supplementary Materials, further inquiries can be directed to the corresponding author.

## Supplementary Materials

The following are available online at https://www.mdpi.com/article/10.3390/ph17111532/s1, Anticonvulsant screening data (Table S1), UPLC/HRMS traces, and ^1^H NMR, ^13^C NMR spectra for target compounds.
