# Supplementary material for: Discovery of New 3-(Benzo[b]Thiophen-2-yl)Pyrrolidine-2,5-Dione Derivatives as Potent Antiseizure and Antinociceptive Agents—In Vitro and In Vivo Evaluation
Source: Pharmaceuticals (Basel). 2024 Nov 15;17(11):1532. doi: 10.3390/ph17111532 (PMC11597642; doi:10.3390/ph17111532)

supplementary materials

Table of contents

Table S1. Anticonvulsant activity screening and neurotoxicity data 3

UPLC/HRMS traces for target final compounds 4

^{1}H NMR, ^{13}C NMR and ^{19}F NMR spectra for all final compounds.................................. 13

Table S1. Anticonvulsant activity screening and neurotoxicity data in the MES and 6 Hz (32 and 44 mA) seizure models in mice *i.p.* pretreatment time–0.5 h.

**Compd**	**NT[a] (100 mg/kg)**	**MES[b] (100 mg/kg)**	**6 Hz (32 mA)[b] (100 mg/kg)**	**6 Hz (44 mA)[b] (200 mg/kg)**
**25**	2/4	1/4	**2/4**	-
**26**	0/4	1/4	0/4	-
**27**	0/4	**2/4**	**2/4**	-
**28**	0/4	**4/4**	**3/4**	0/4
**29**	0/4	0/4	0/4	-
**30**	0/4	**4/4**	**2/4**	0/4
**31**	2/4	**4/4**	**4/4**	-
**32**	0/4	**2/4**	**3/4**	1/4
**33**	0/4	**4/4**	**3/4**	1/4

Ratios where at least 50% of animals were protected have been highlighted in bold for easier data interpretation. [a]The data indicate: number of mice in which motor impairment was observed /number of mice tested in the rotarod model. [b]The data indicate: number of mice protected against seizures/number of mice tested.

UPLC/HRMS traces for target final compounds

3-(Benzo[b]thiophen-2-yl)-1-(2-(4-(3-(trifluoromethyl)phenyl)piperazin-1-yl)ethyl)pyrrolidine-2,5-dione hydrochloride (25)

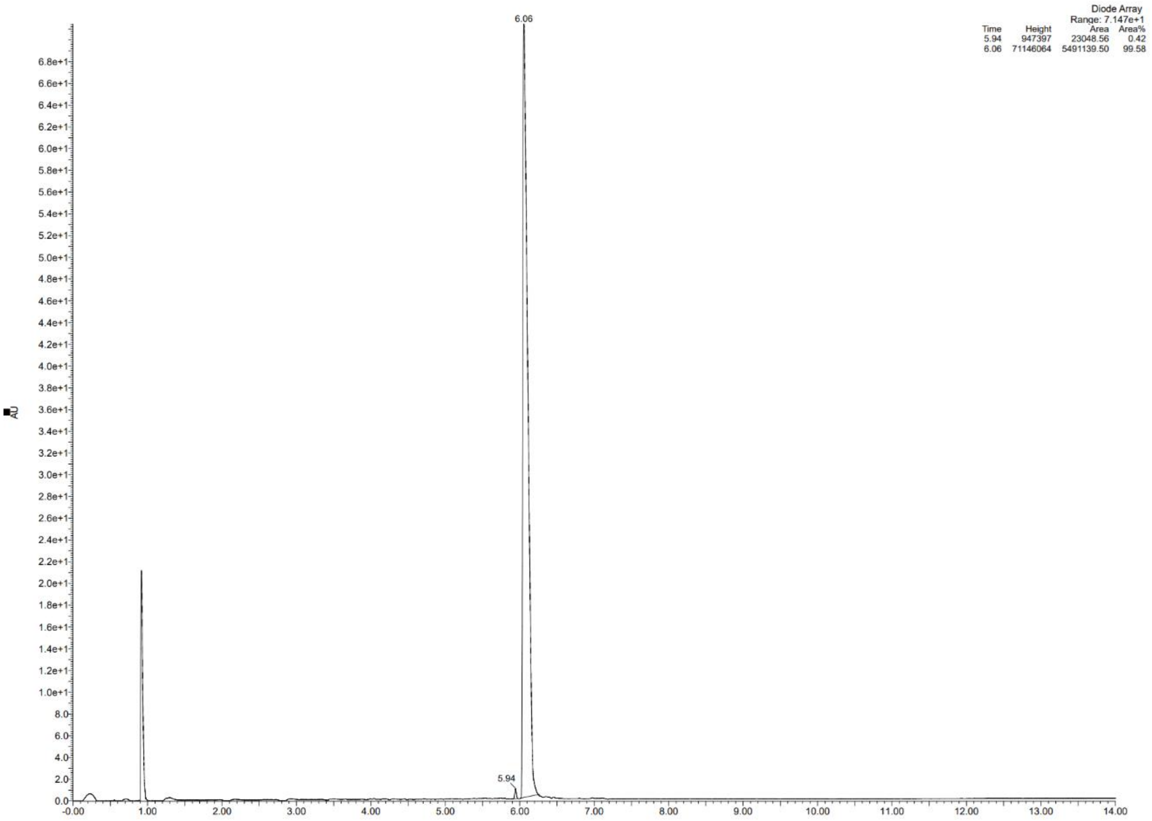

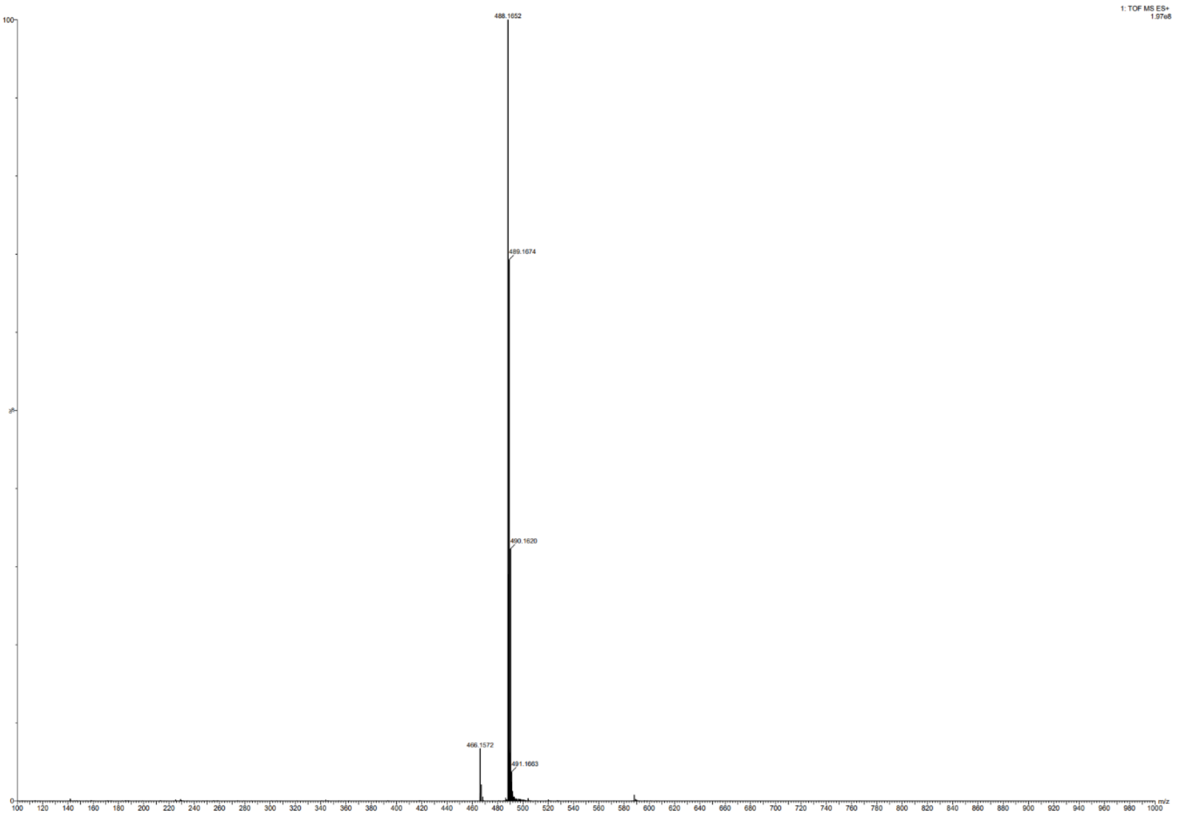

3-(Benzo[b]thiophen-2-yl)-1-(3-(4-(3-(trifluoromethyl)phenyl)piperazin-1-yl)propyl)pyrrolidine-2,5-dione hydrochloride (26)

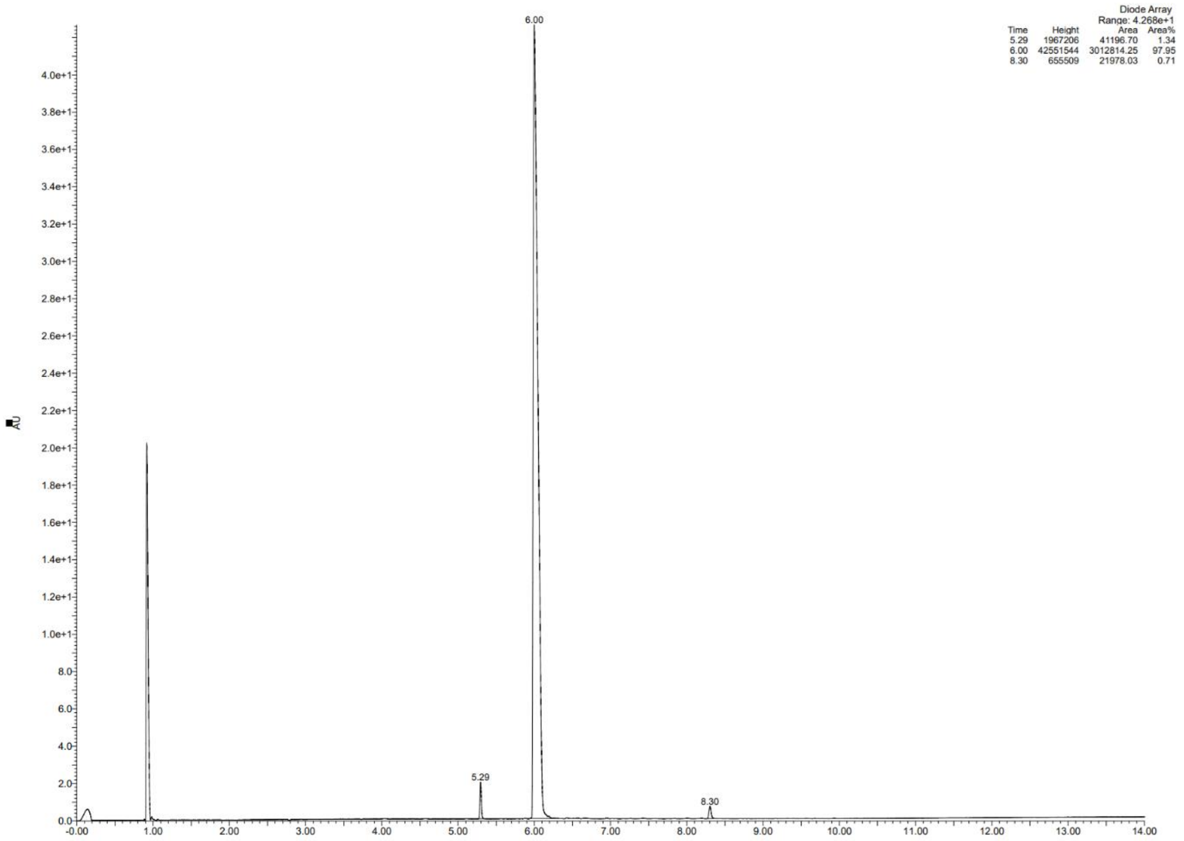

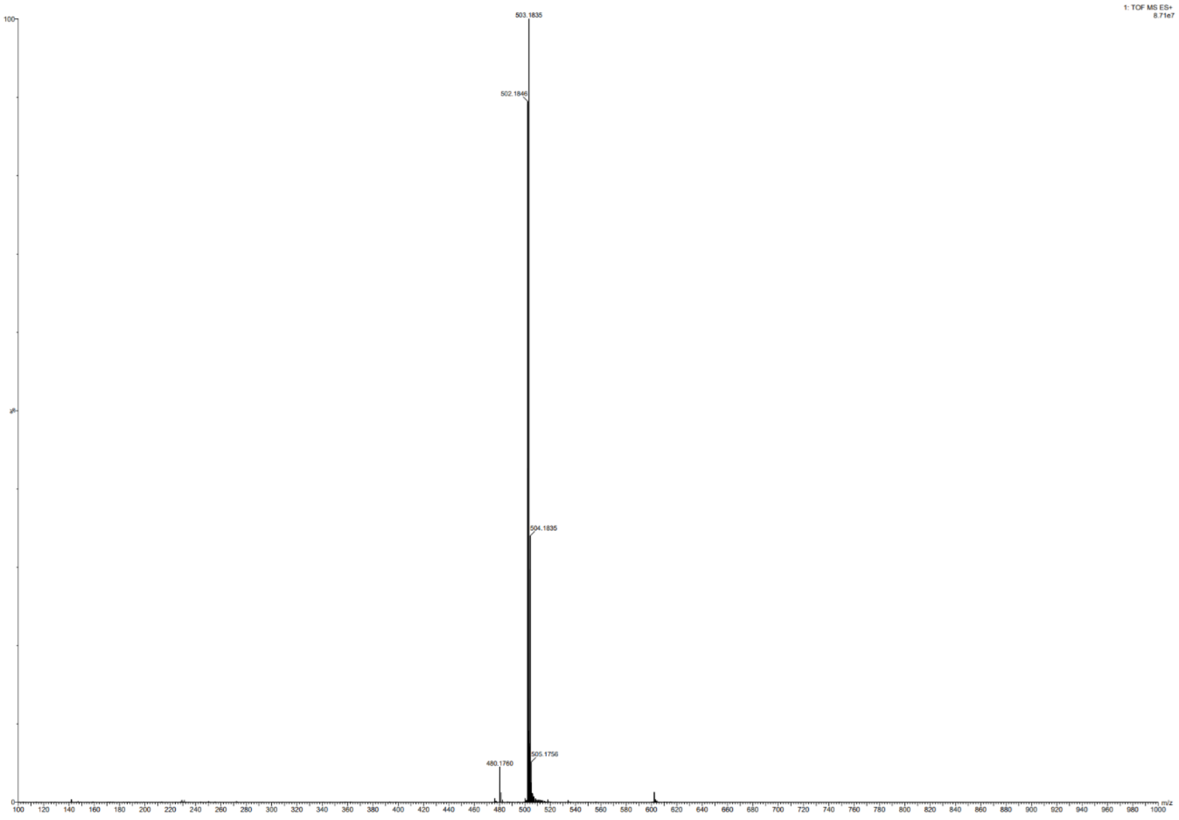

3-(Benzo[b]thiophen-2-yl)-1-(2-(4-(3-(trifluoromethoxy)phenyl)piperazin-1-yl)ethyl)pyrrolidine-2,5-dione hydrochloride (27)

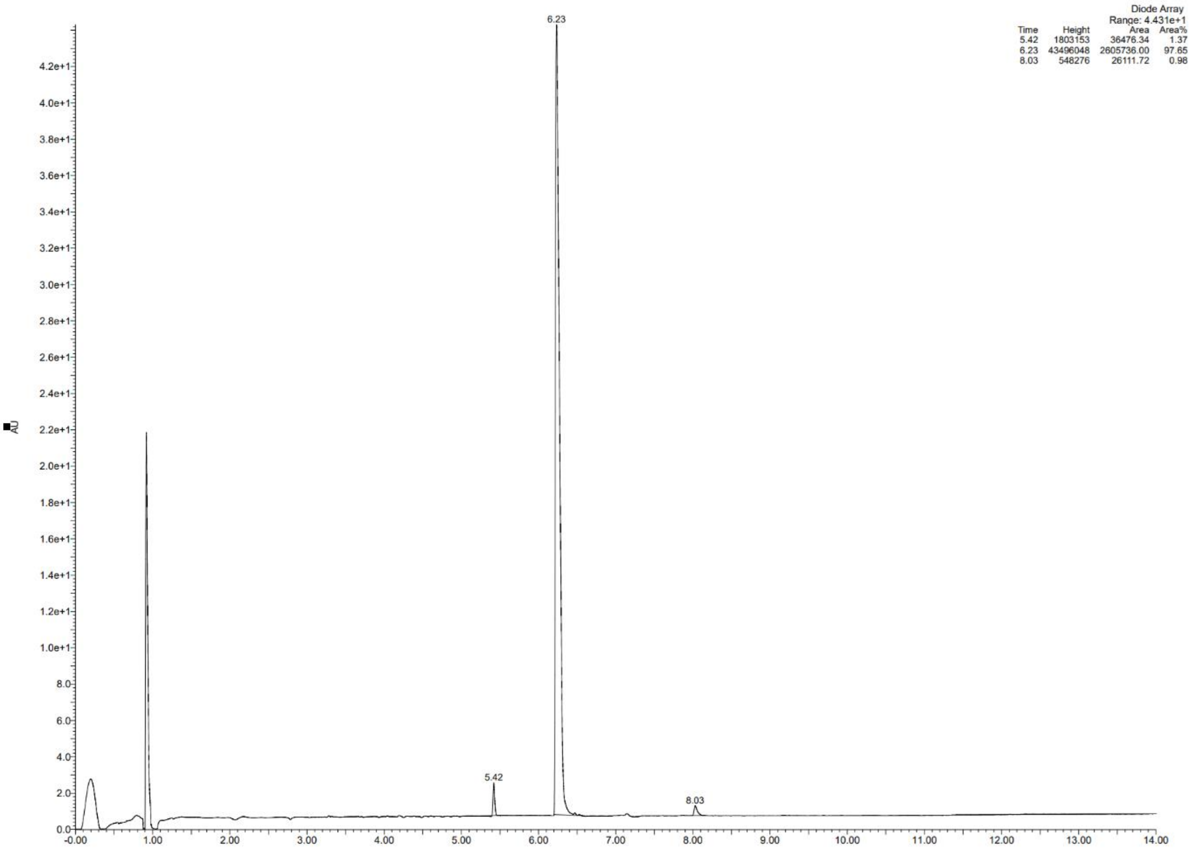

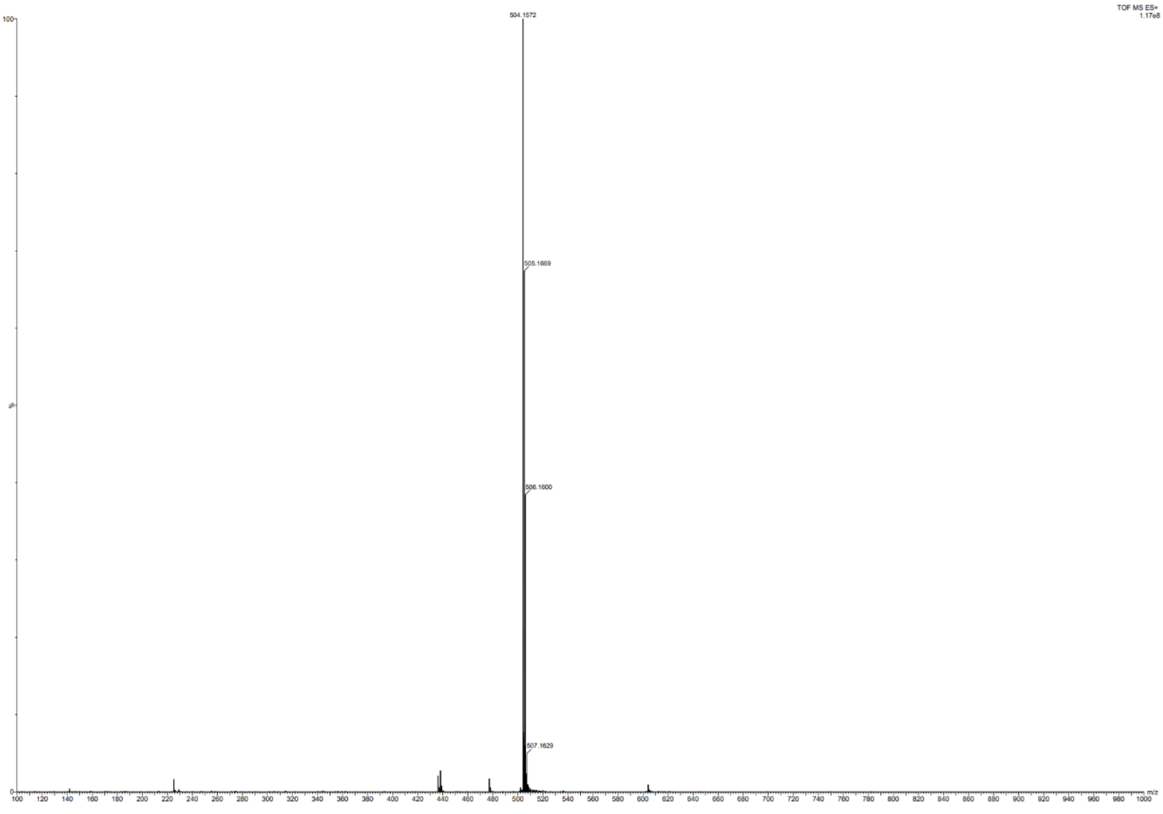

3-(Benzo[b]thiophen-2-yl)-1-(3-(4-(3-(trifluoromethoxy)phenyl)piperazin-1-yl)propyl)pyrrolidine-2,5-dione hydrochloride (28)

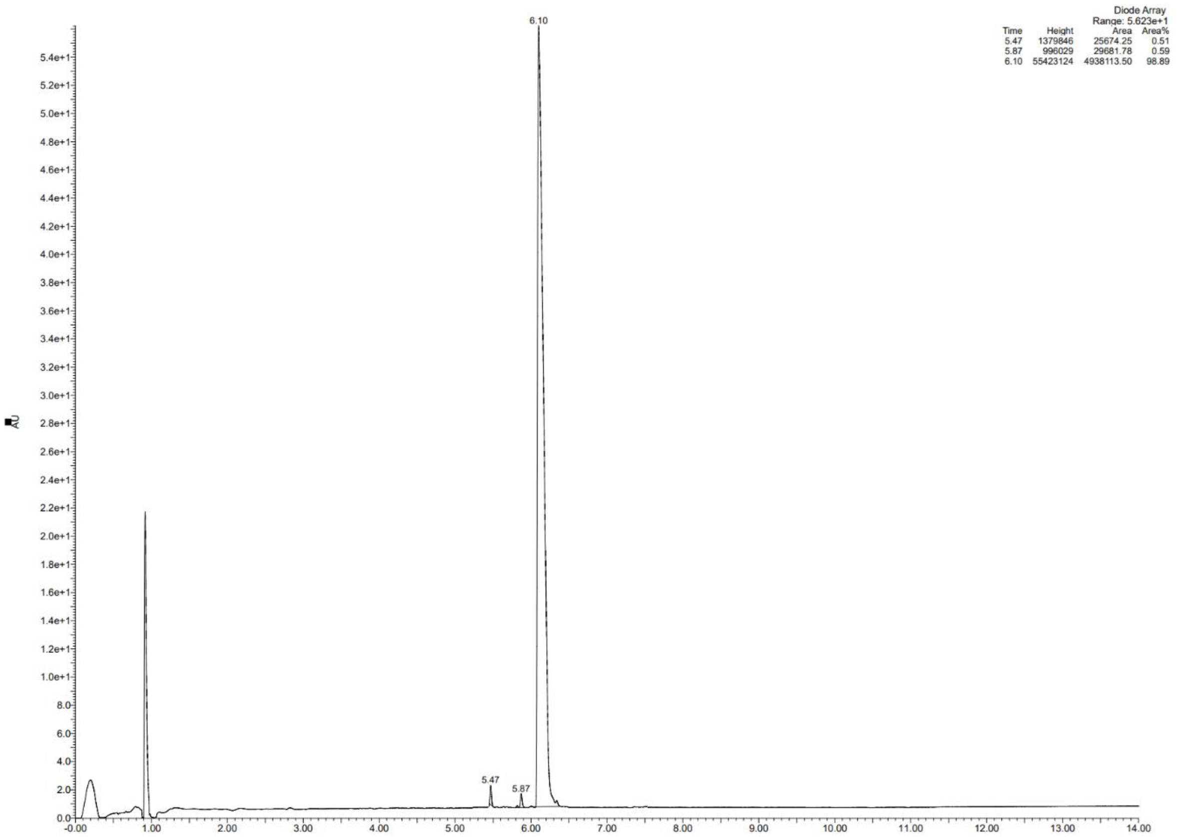

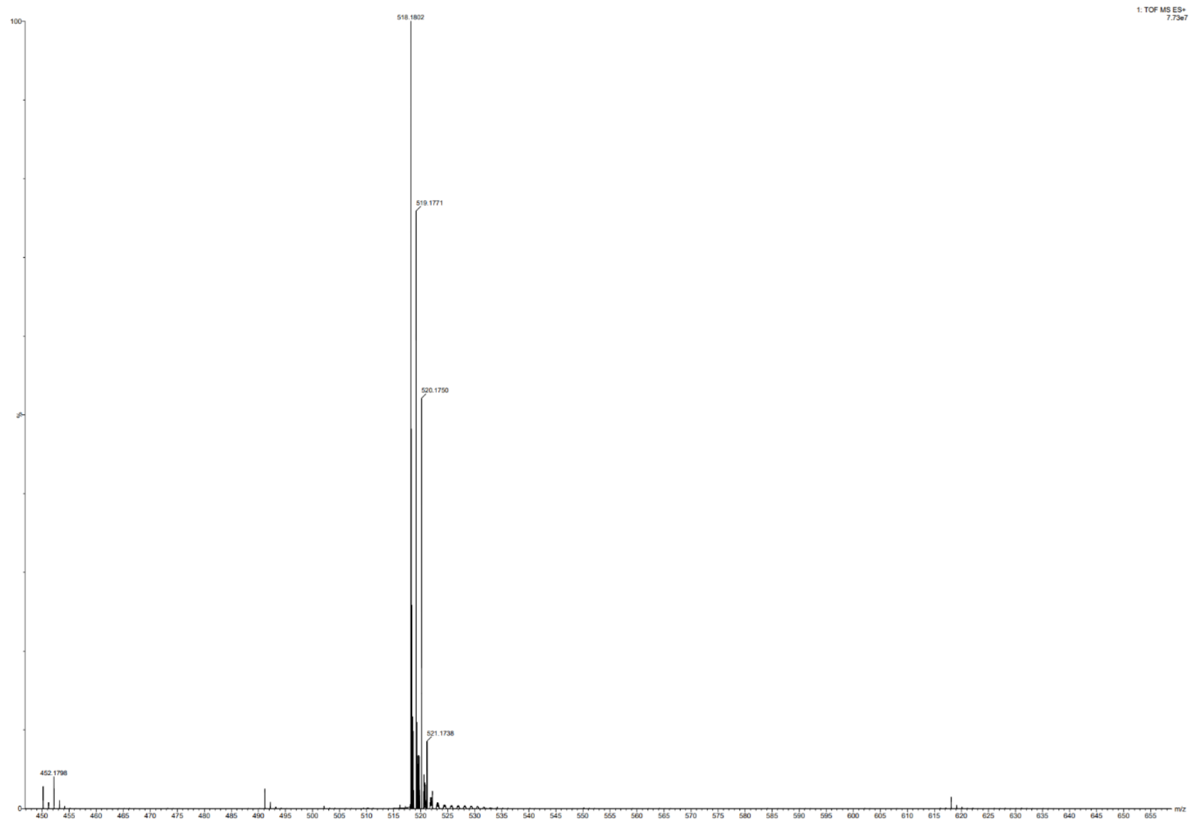

3-(Benzo[b]thiophen-2-yl)-1-(2-(4-(3-((trifluoromethyl)thio)phenyl)piperazin-1-yl)ethyl)pyrrolidine-2,5-dione hydrochloride (29)

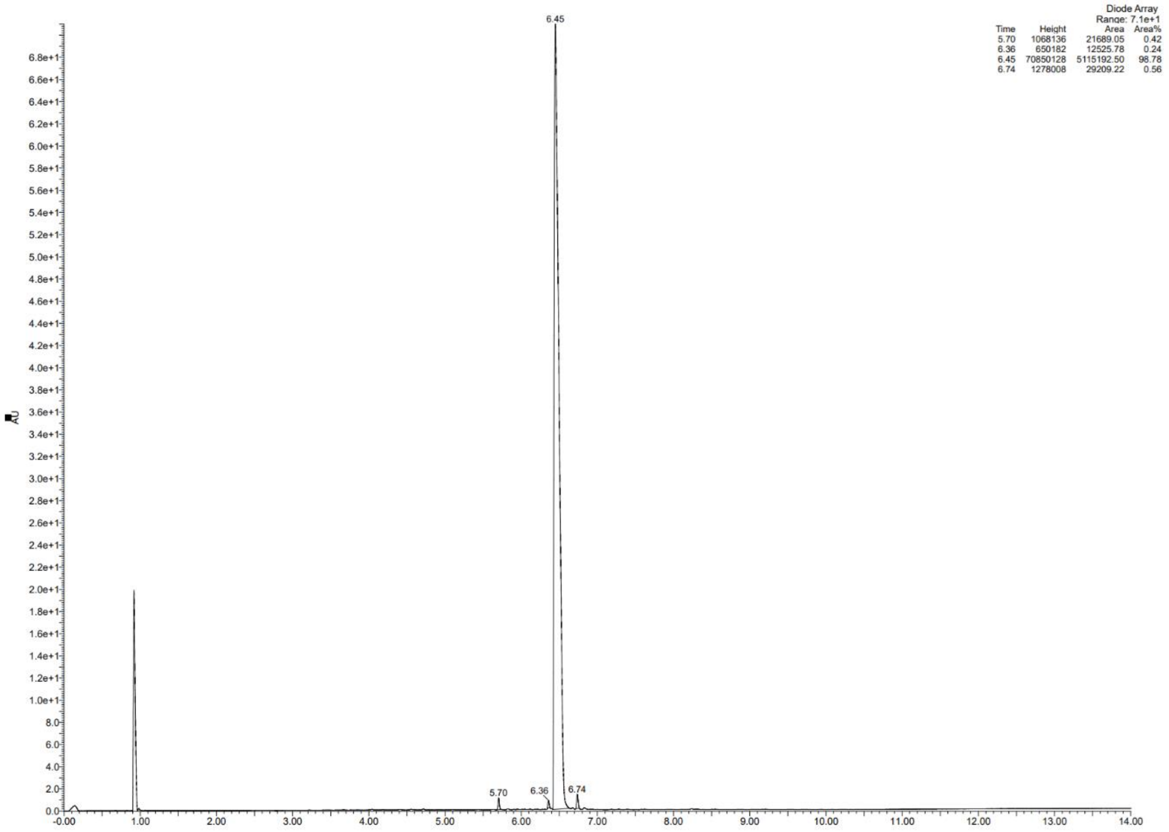

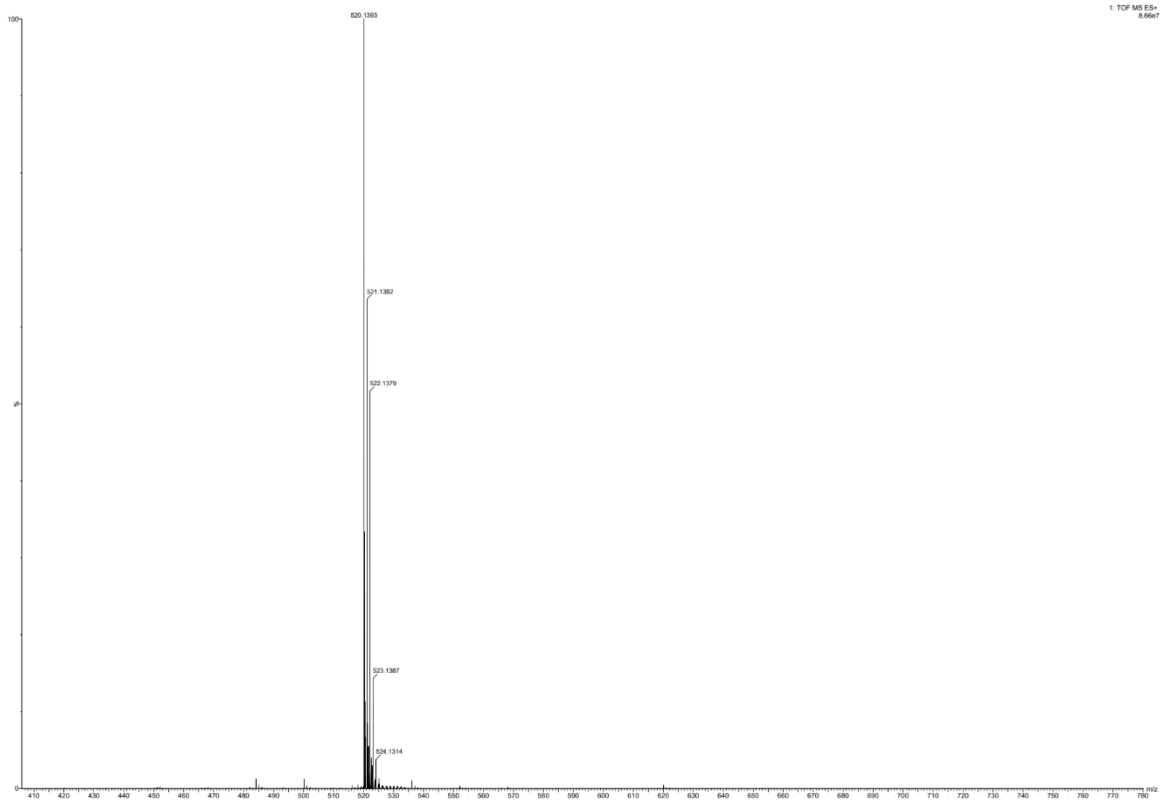

3-(Benzo[b]thiophen-2-yl)-1-(3-(4-(3-((trifluoromethyl)thio)phenyl)piperazin-1-yl)propyl)pyrrolidine-2,5-dione hydrochloride (30)

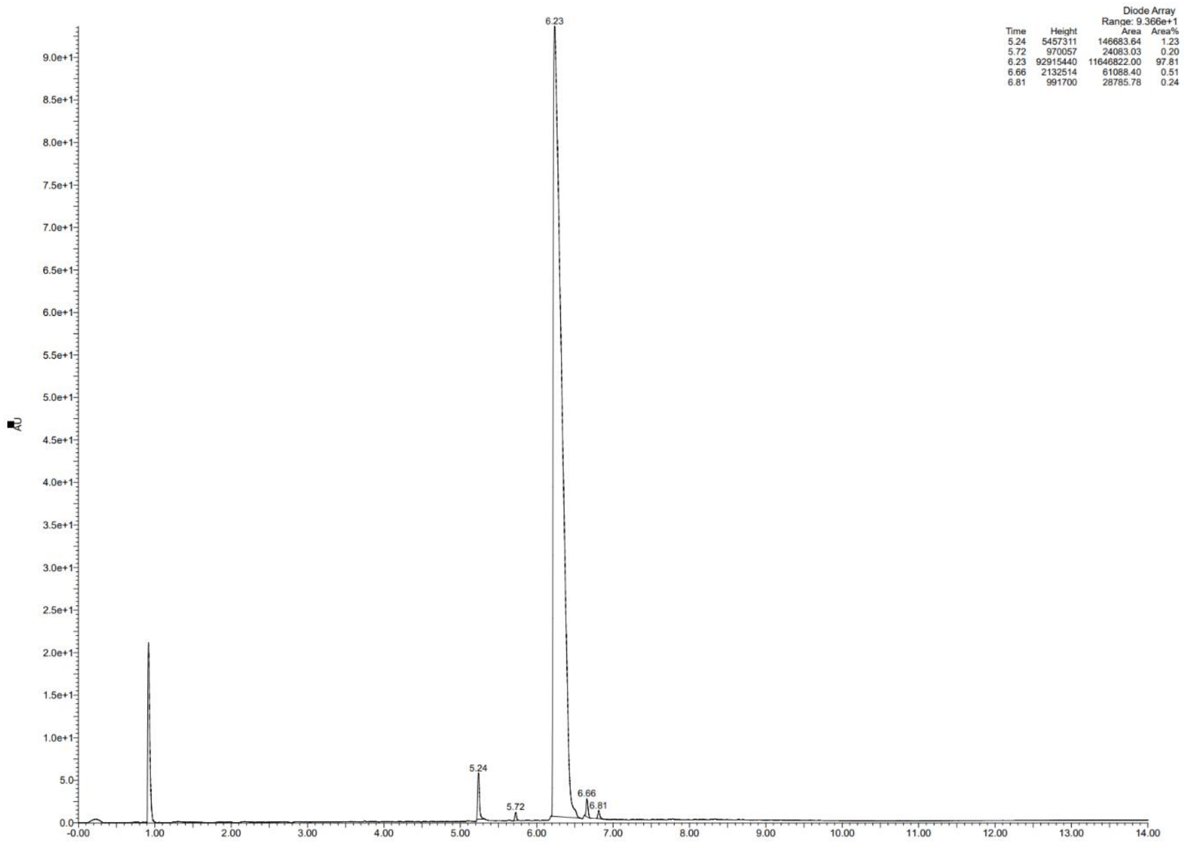

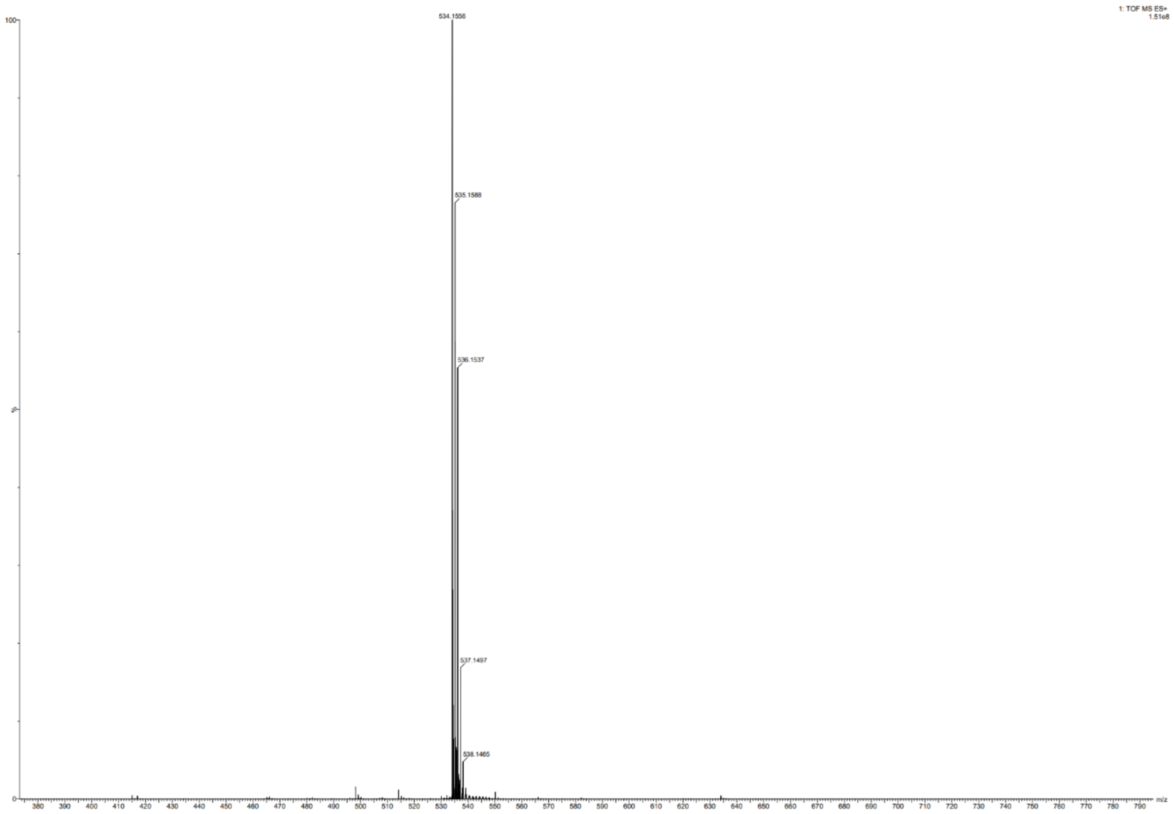

3-(Benzofuran-2-yl)-1-(3-(4-(3-(trifluoromethoxy)phenyl)piperazin-1-yl)propyl)pyrrolidine-2,5-dione hydrochloride (31)

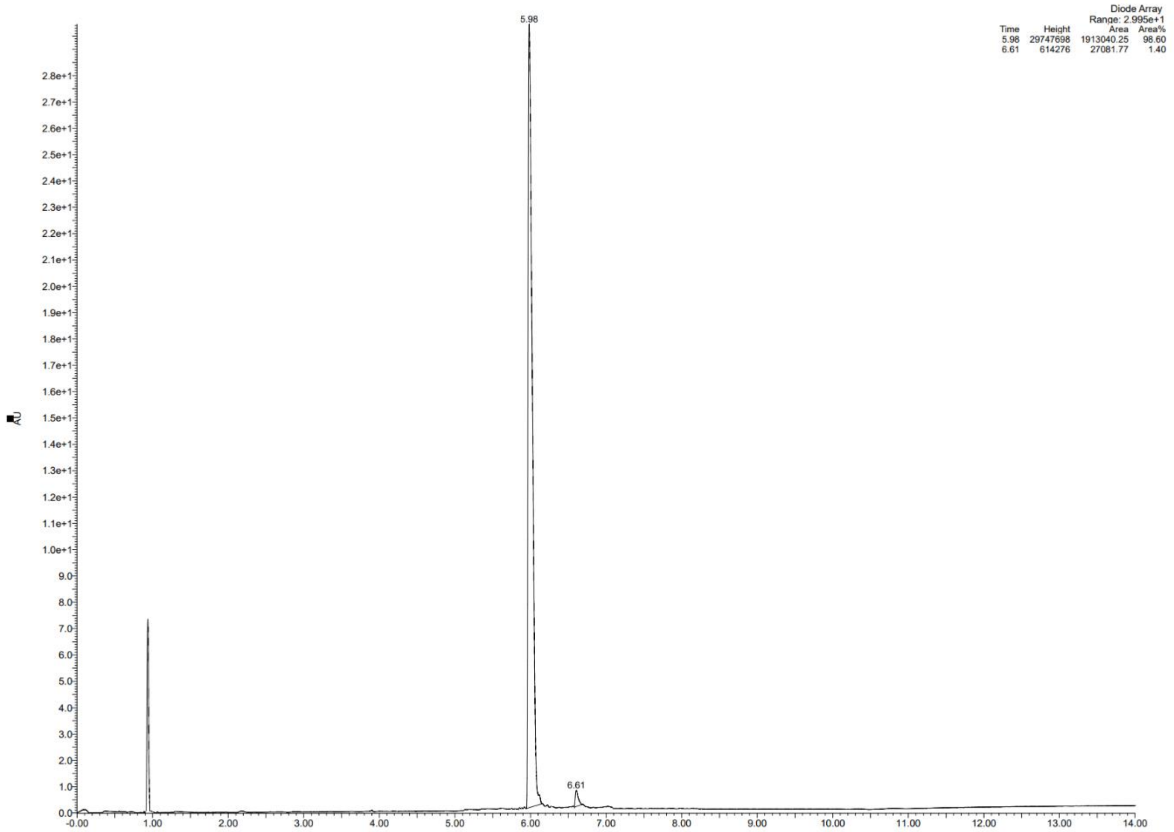

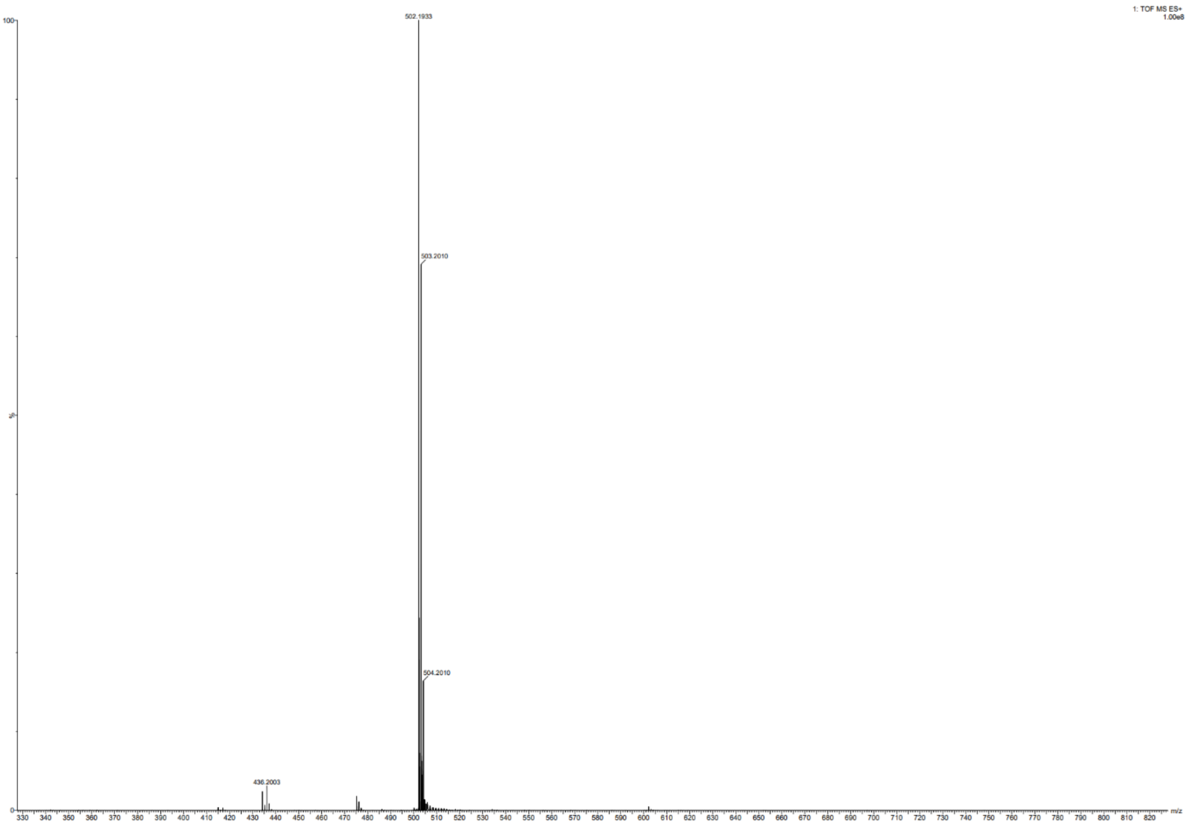

3-(Benzo[b]thiophen-2-yl)-1-(2-morpholinoethyl)pyrrolidine-2,5-dione hydrochloride (32)

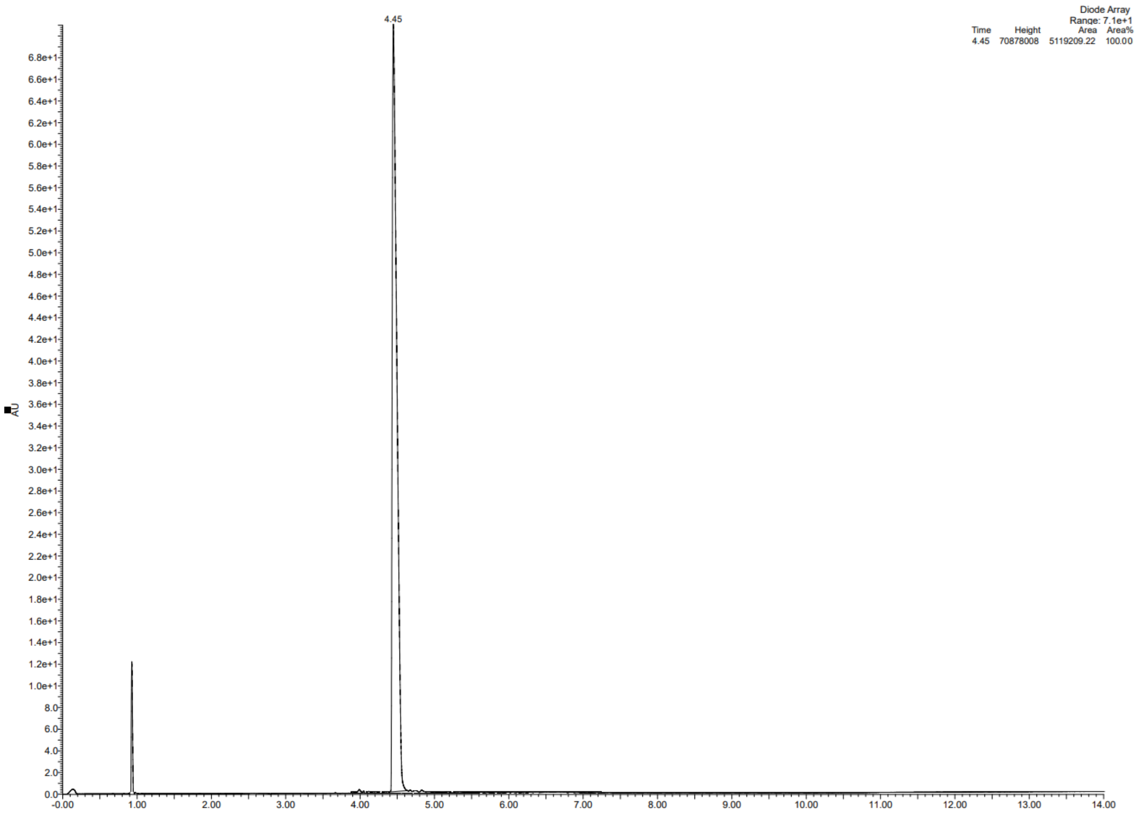

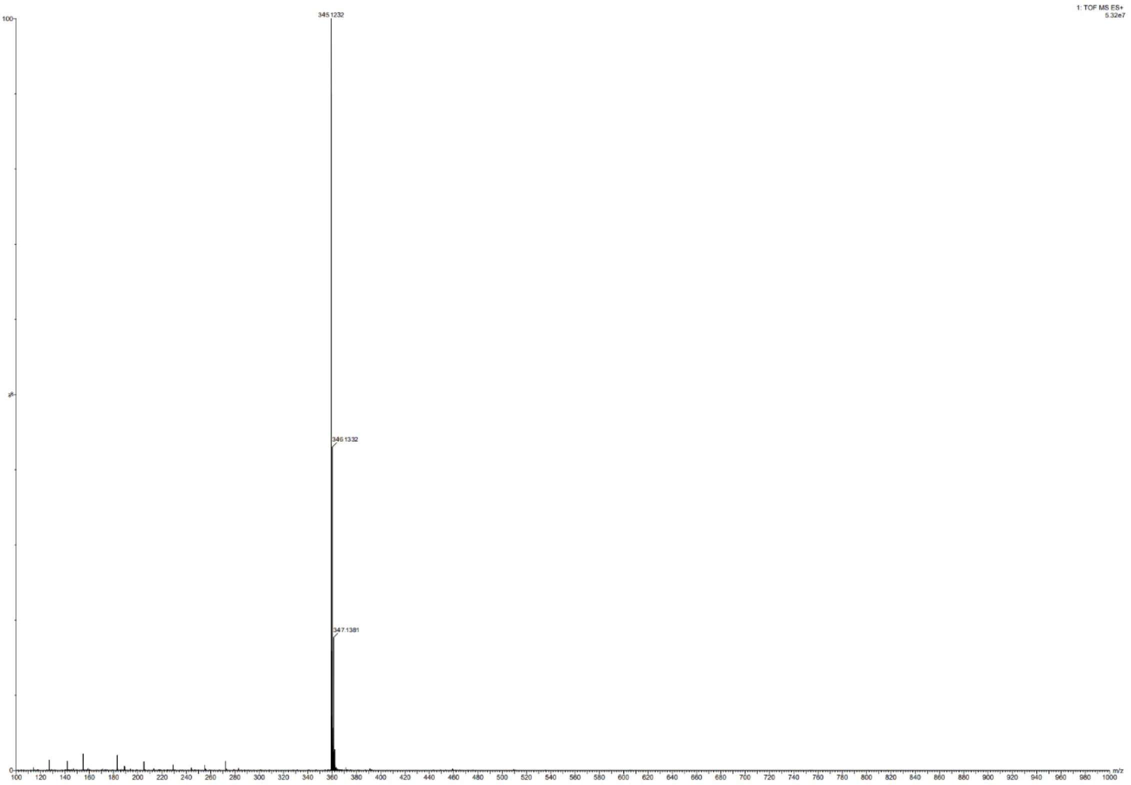

3-(Benzo[b]thiophen-2-yl)-1-(3-morpholinopropyl)pyrrolidine-2,5-dione hydrochloride (33)

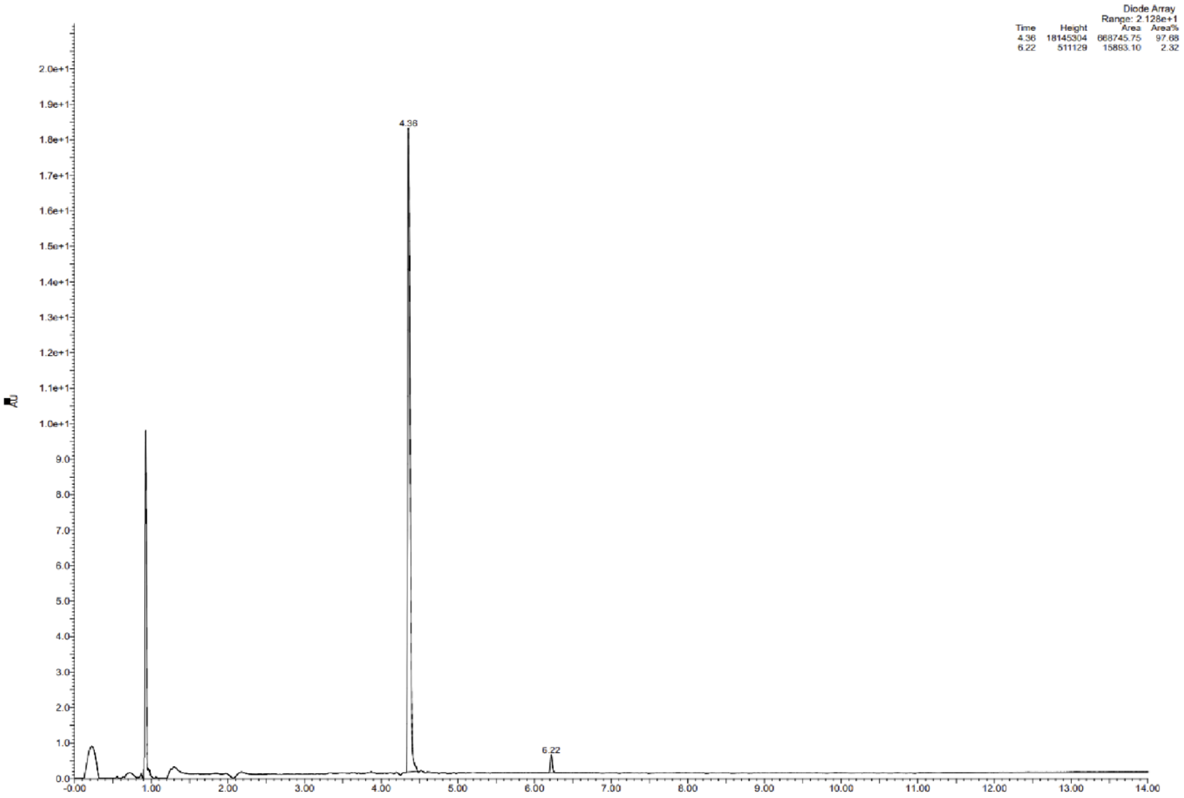

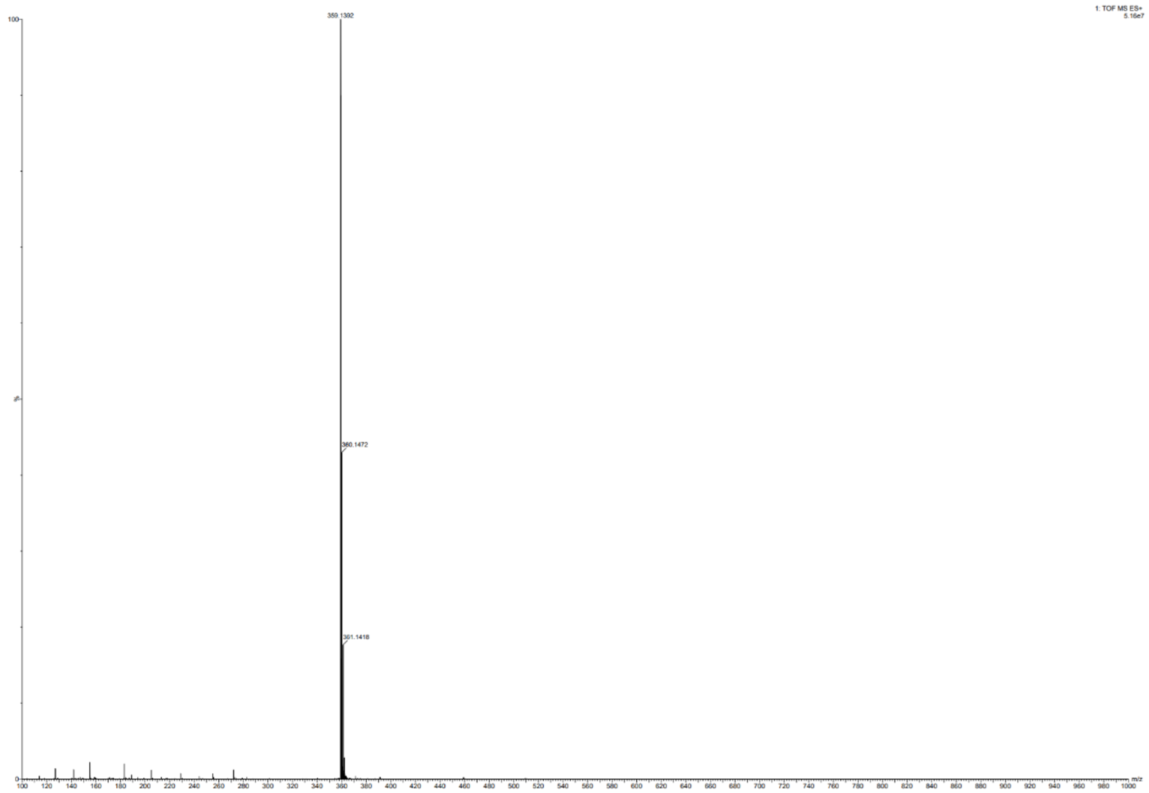

^{1}H NMR, ^{13}C NMR and ^{19}F NMR spectra for all final compounds

3-(Benzo[b]thiophen-2-yl)-1-(2-(4-(3-(trifluoromethyl)phenyl)piperazin-1-yl)ethyl)pyrrolidine-2,5-dione (25) – ^{1}H NMR

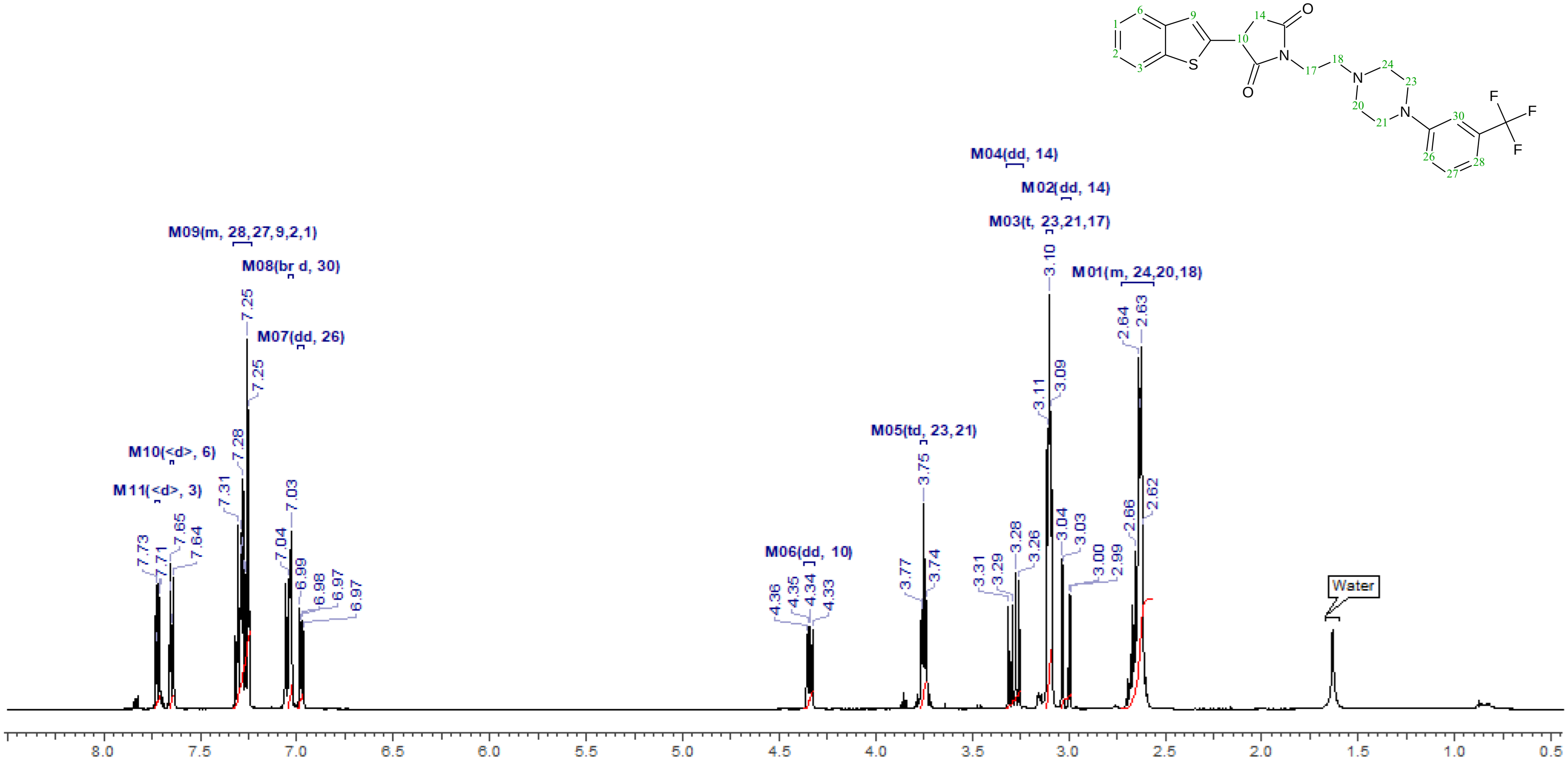

3-(Benzo[b]thiophen-2-yl)-1-(2-(4-(3-(trifluoromethyl)phenyl)piperazin-1-yl)ethyl)pyrrolidine-2,5-dione (25)– ^{13}C NMR

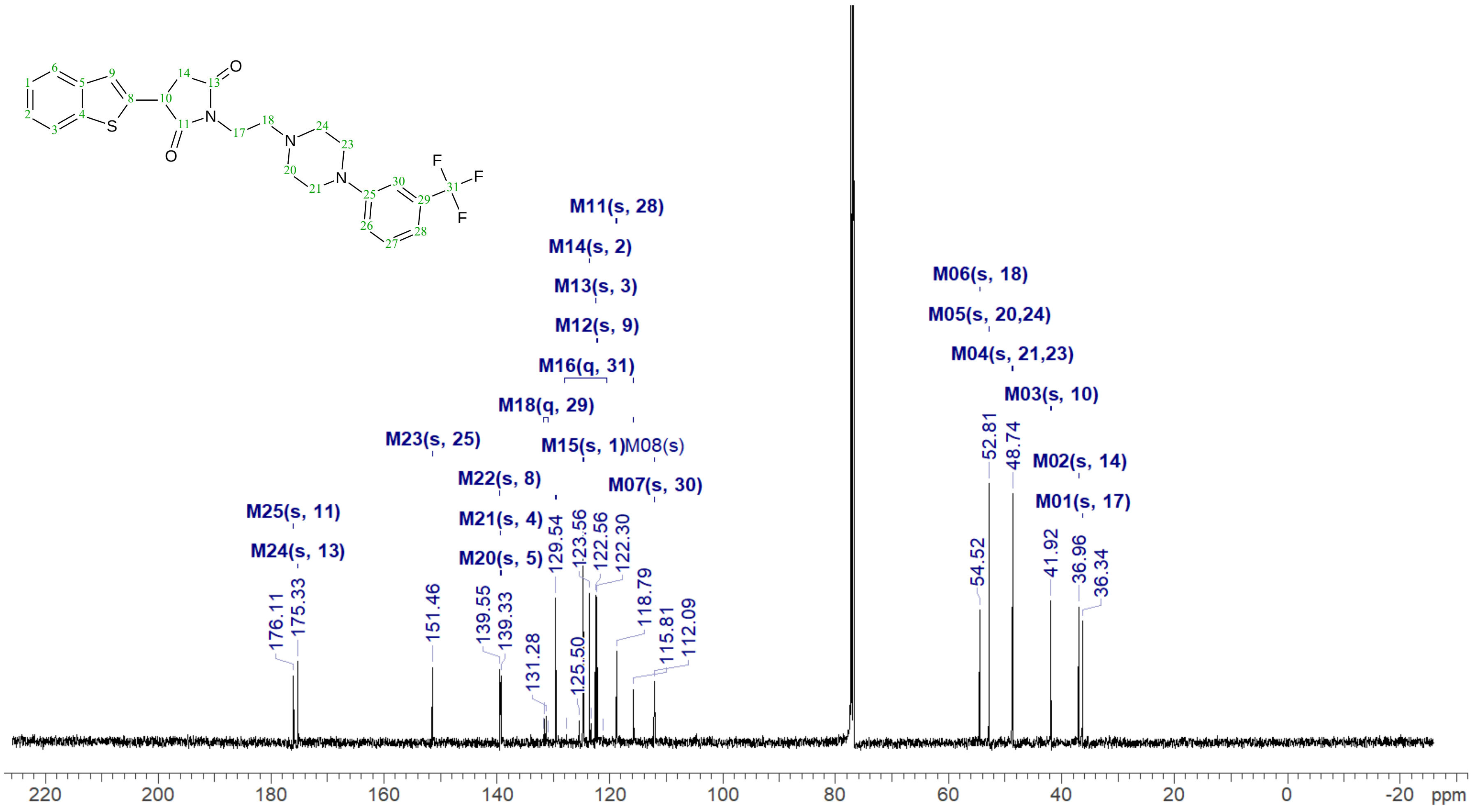

3-(Benzo[b]thiophen-2-yl)-1-(2-(4-(3-(trifluoromethyl)phenyl)piperazin-1-yl)ethyl)pyrrolidine-2,5-dione (25)– ^{19}F NMR

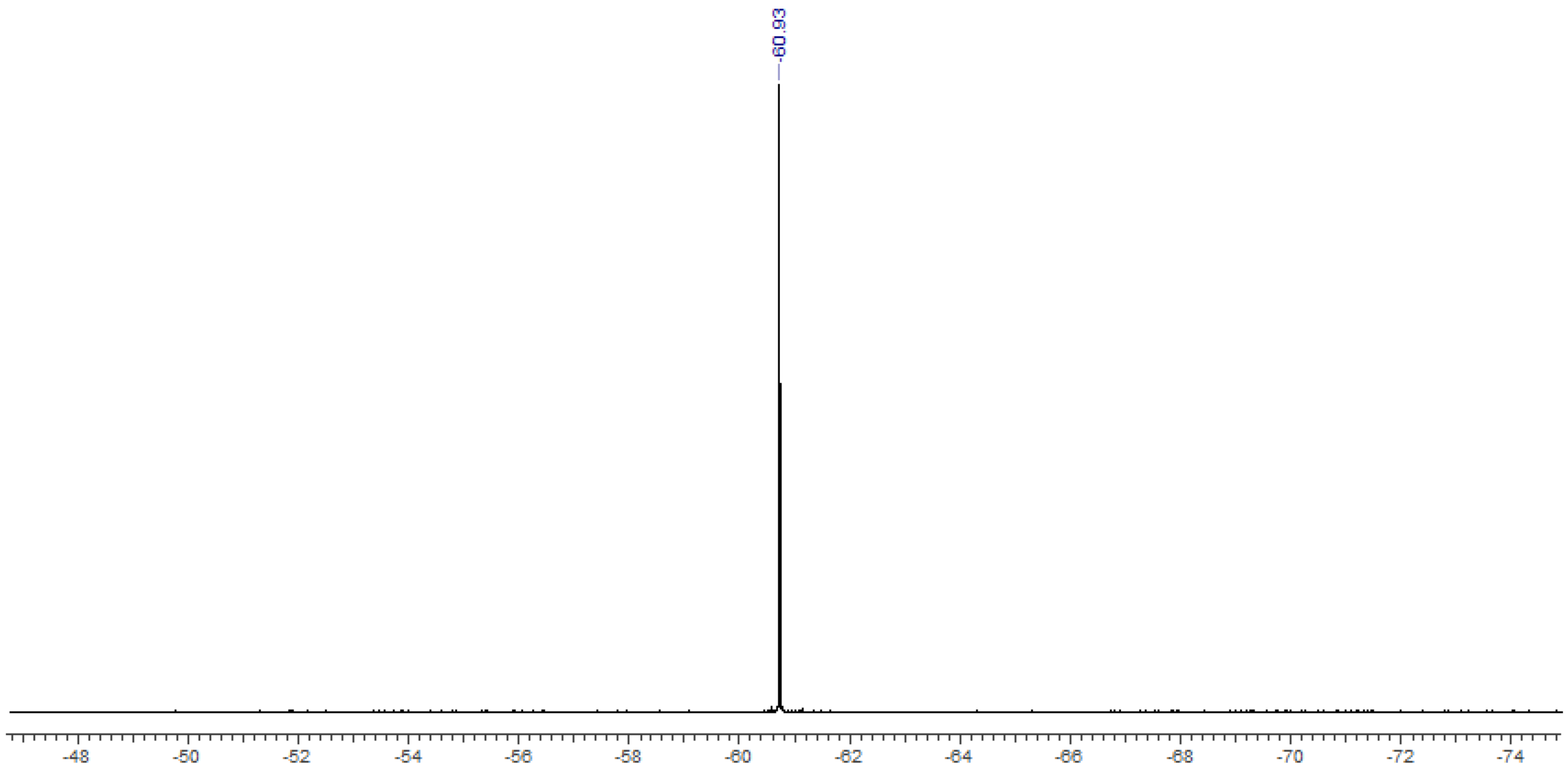

3-(Benzo[b]thiophen-2-yl)-1-(3-(4-(3-(trifluoromethyl)phenyl)piperazin-1-yl)propyl)pyrrolidine-2,5-dione (26) – ^{1}H NMR

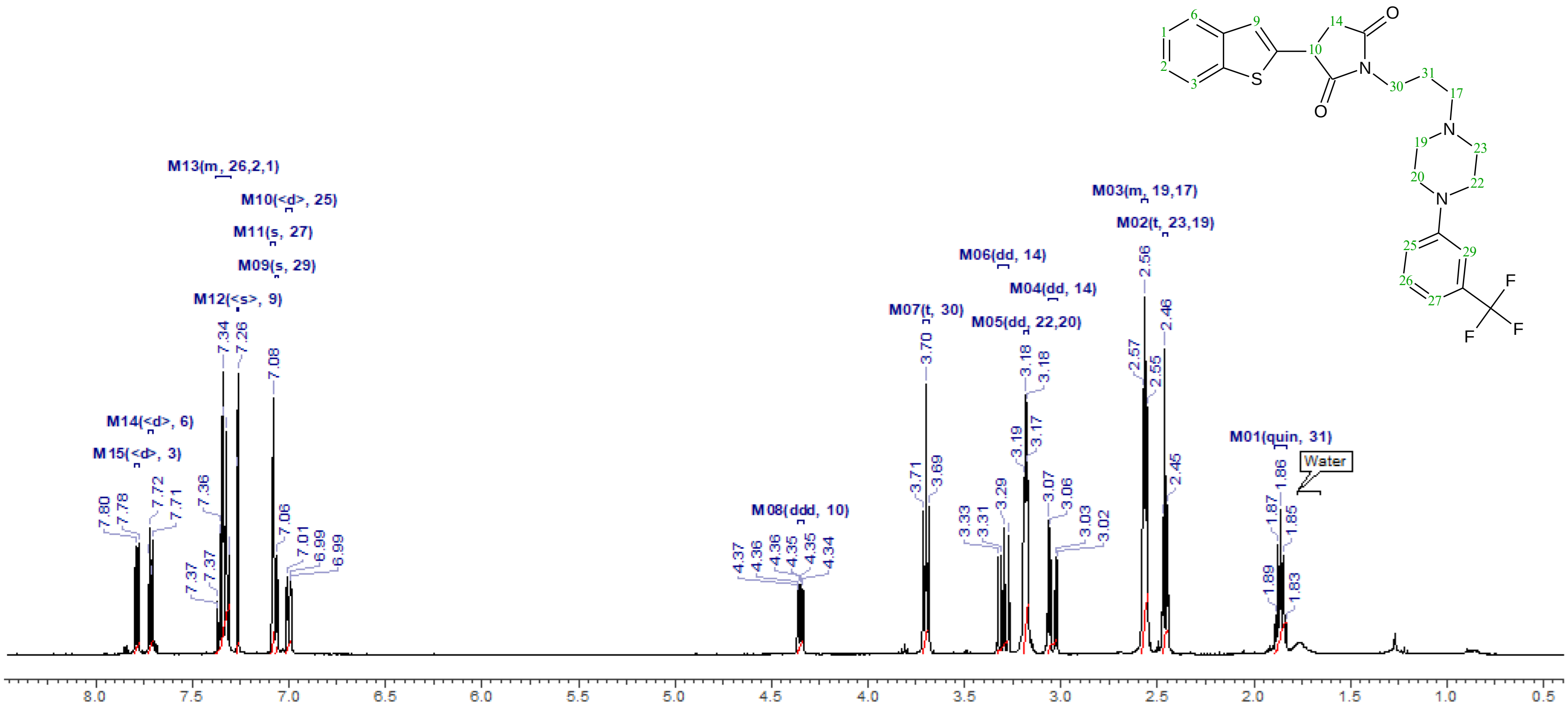

3-(Benzo[b]thiophen-2-yl)-1-(3-(4-(3-(trifluoromethyl)phenyl)piperazin-1-yl)propyl)pyrrolidine-2,5-dione (26) – ^{13}C NMR

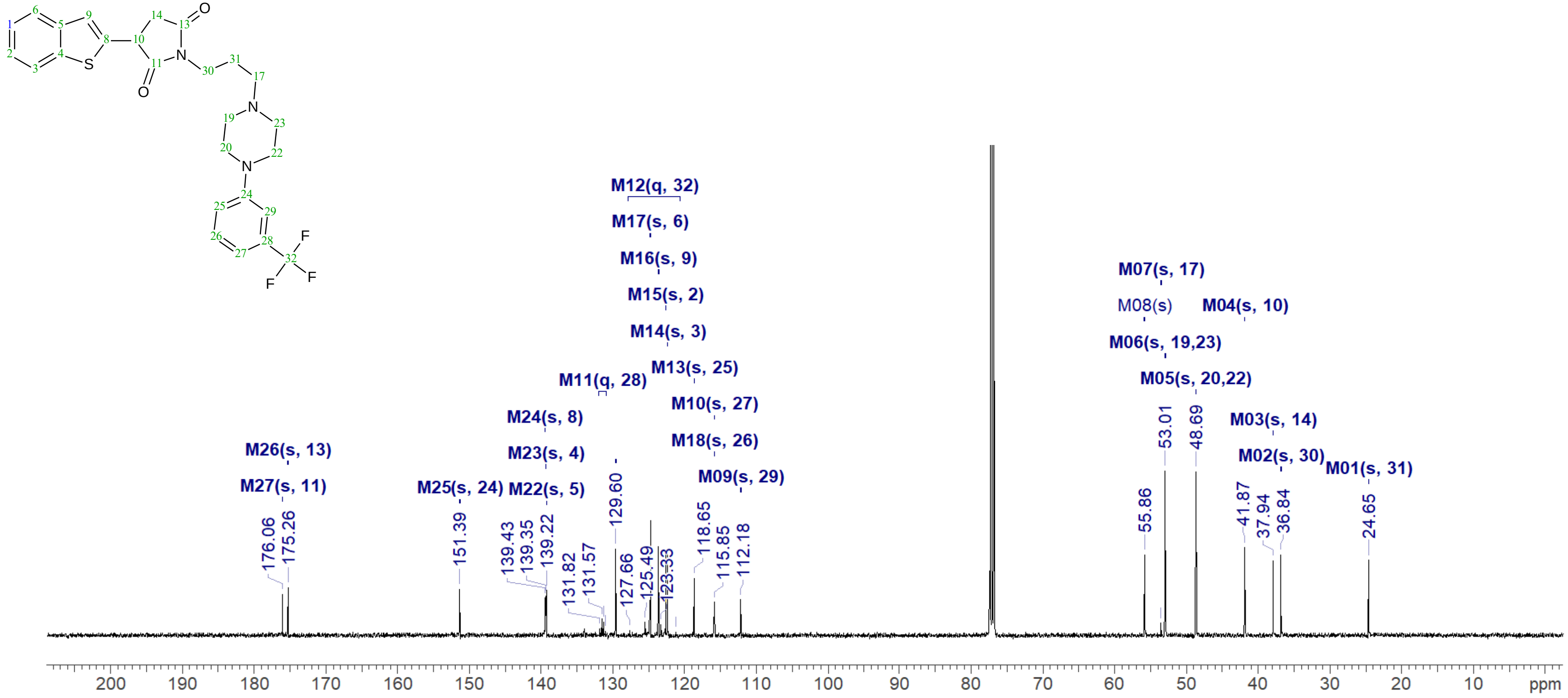

3-(Benzo[b]thiophen-2-yl)-1-(3-(4-(3-(trifluoromethyl)phenyl)piperazin-1-yl)propyl)pyrrolidine-2,5-dione (26) – ^{19}F NMR

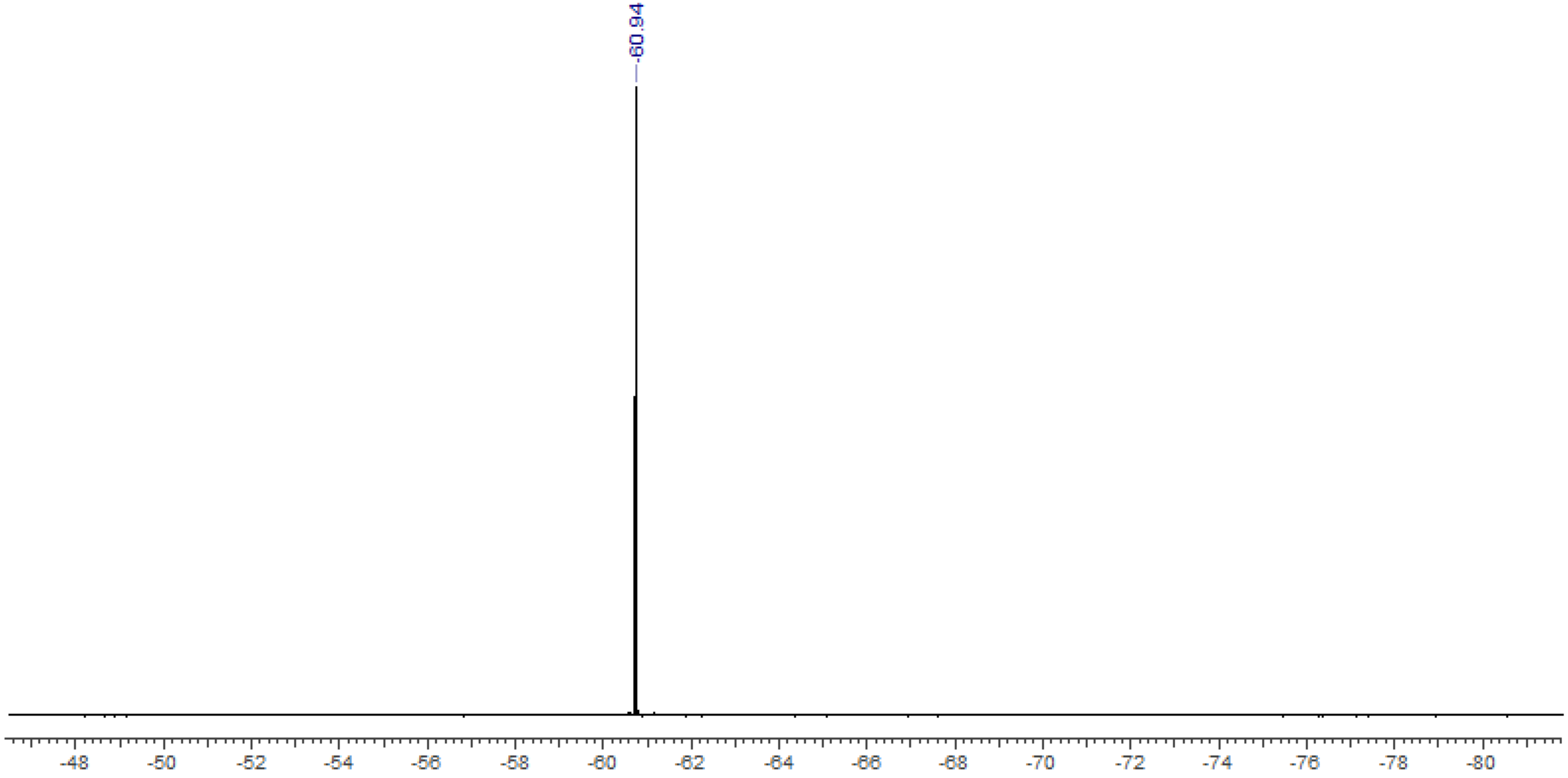

3-(Benzo[b]thiophen-2-yl)-1-(2-(4-(3-(trifluoromethoxy)phenyl)piperazin-1-yl)ethyl)pyrrolidine-2,5-dione (27) – ^{1}H NMR

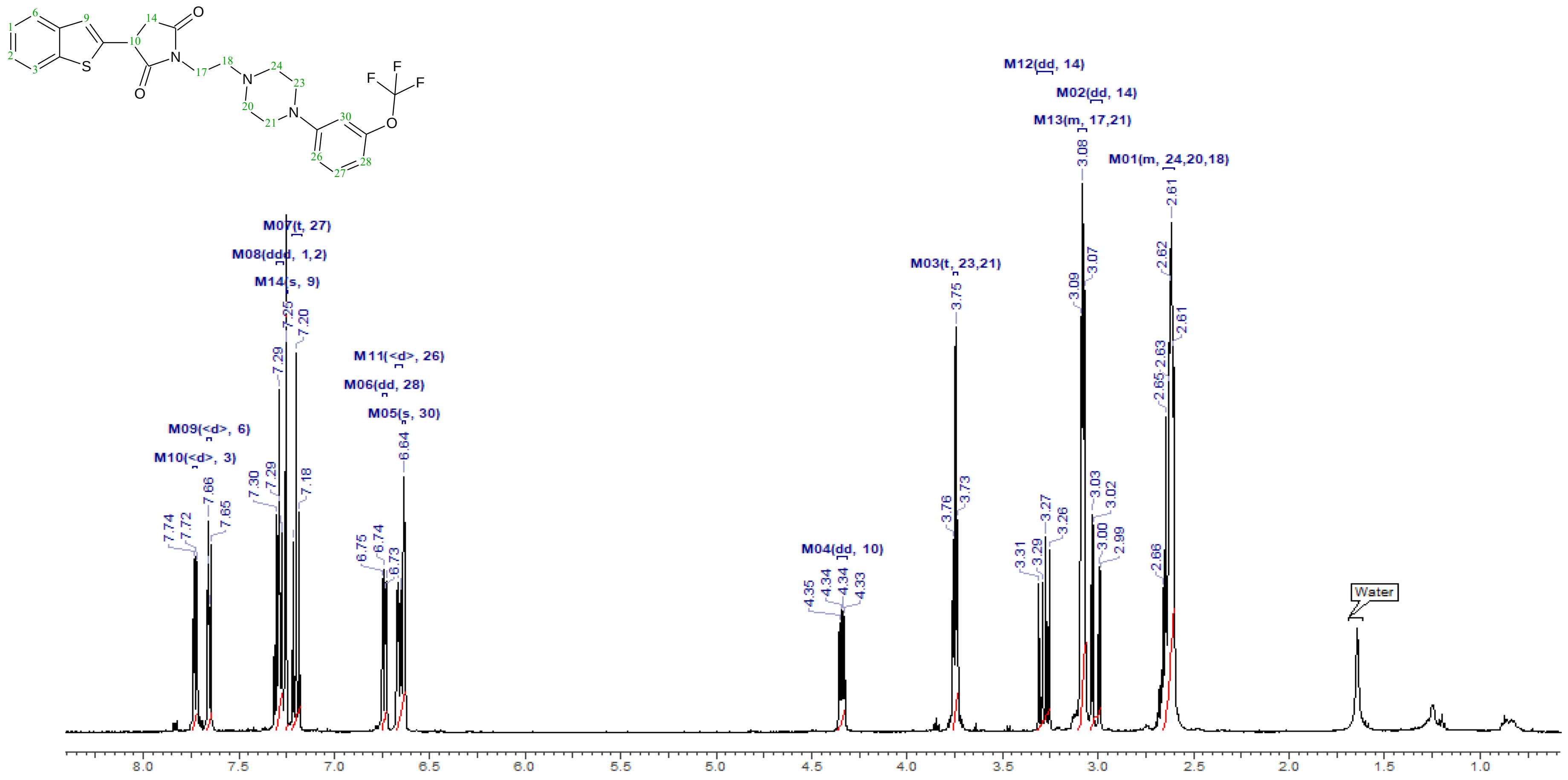

3-(Benzo[b]thiophen-2-yl)-1-(2-(4-(3-(trifluoromethoxy)phenyl)piperazin-1-yl)ethyl)pyrrolidine-2,5-dione (27) – ^{13}C NMR

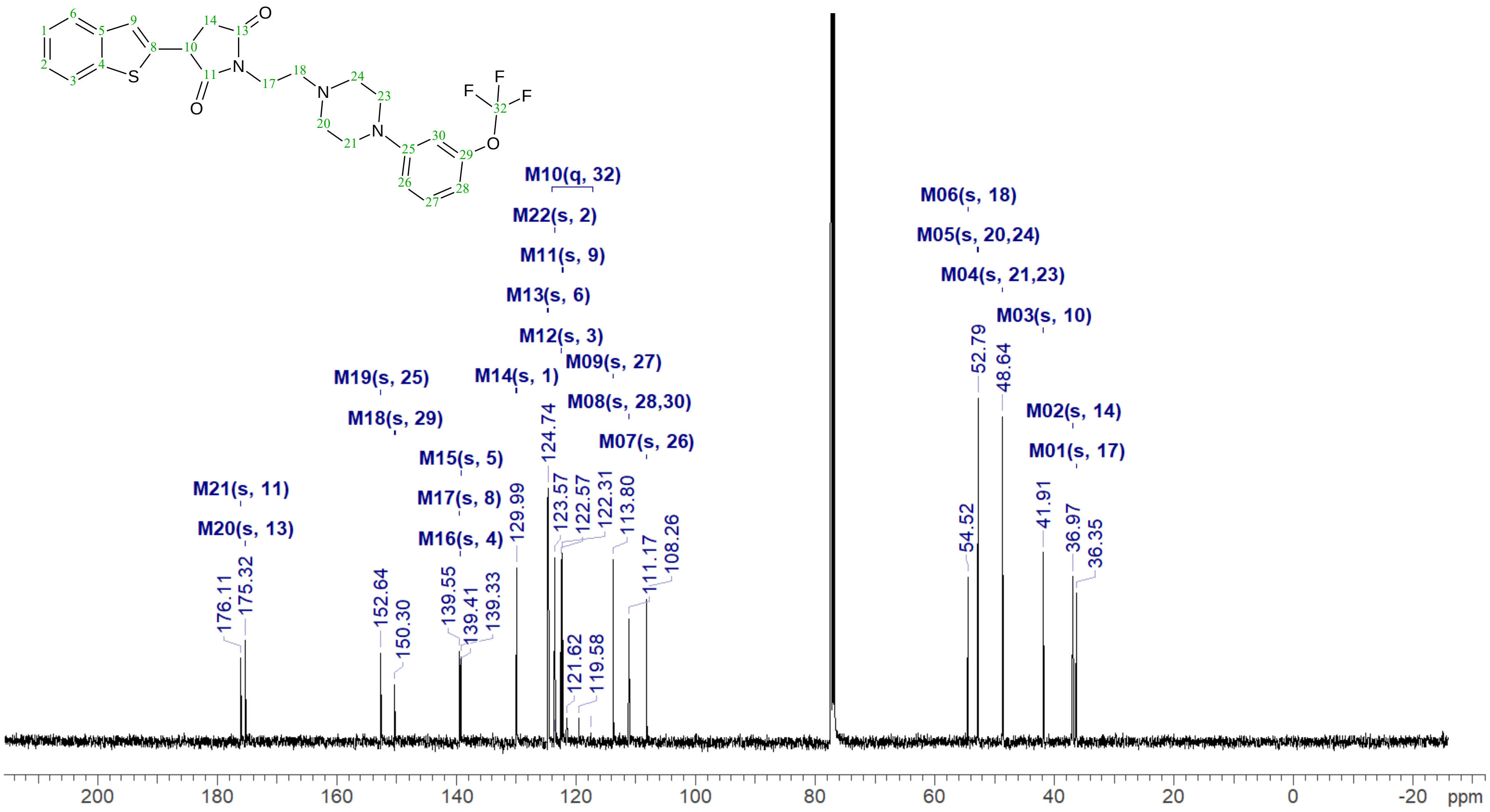

3-(Benzo[b]thiophen-2-yl)-1-(2-(4-(3-(trifluoromethoxy)phenyl)piperazin-1-yl)ethyl)pyrrolidine-2,5-dione (27) – ^{19}F NMR

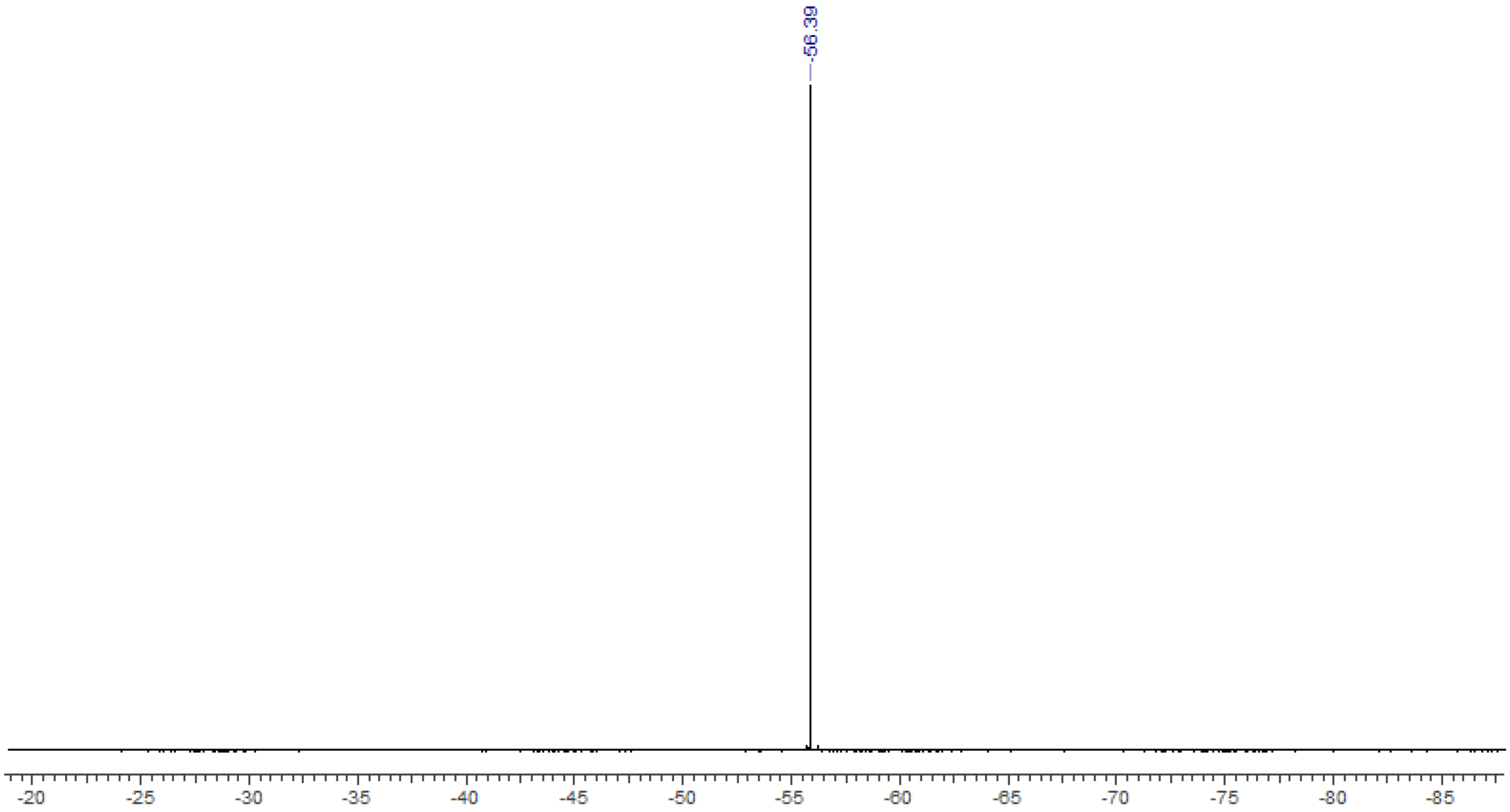

3-(Benzo[b]thiophen-2-yl)-1-(3-(4-(3-(trifluoromethoxy)phenyl)piperazin-1-yl)propyl)pyrrolidine-2,5-dione (28) – ^{1}H NMR

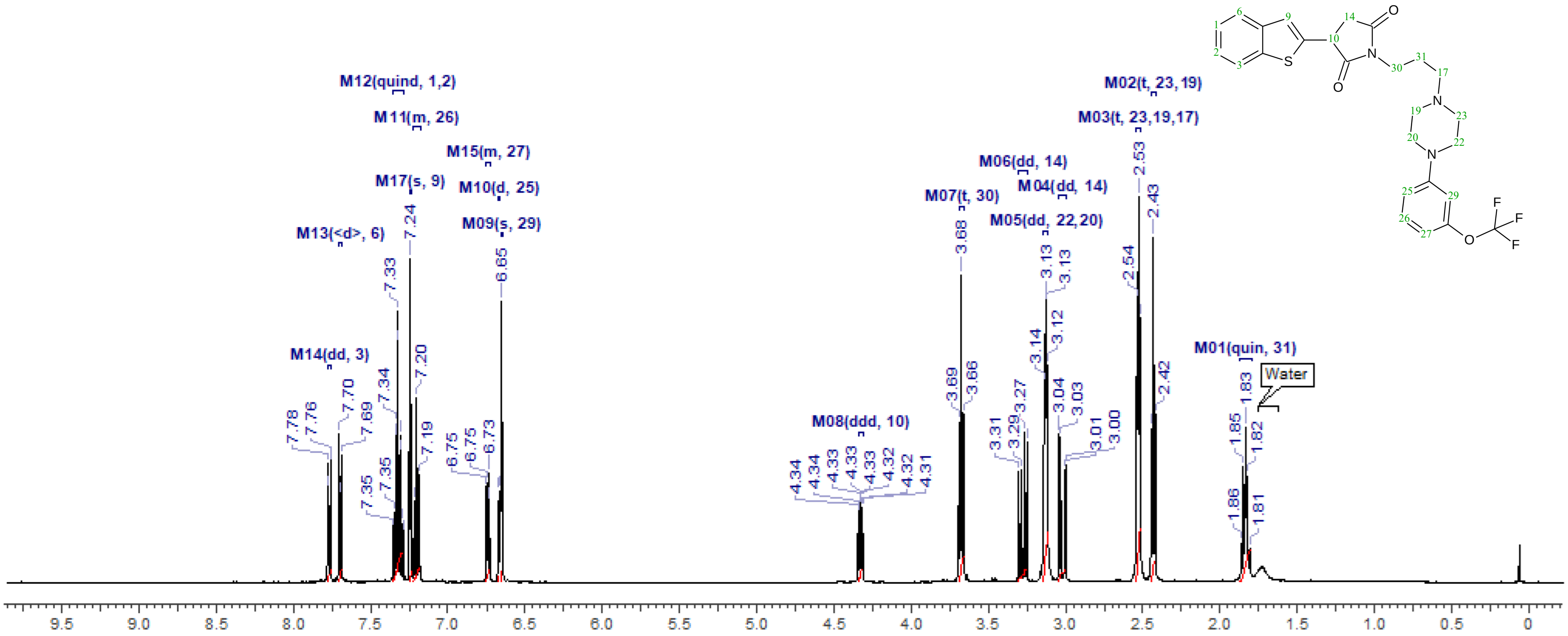

3-(Benzo[b]thiophen-2-yl)-1-(3-(4-(3-(trifluoromethoxy)phenyl)piperazin-1-yl)propyl)pyrrolidine-2,5-dione (28) – ^{13}C NMR

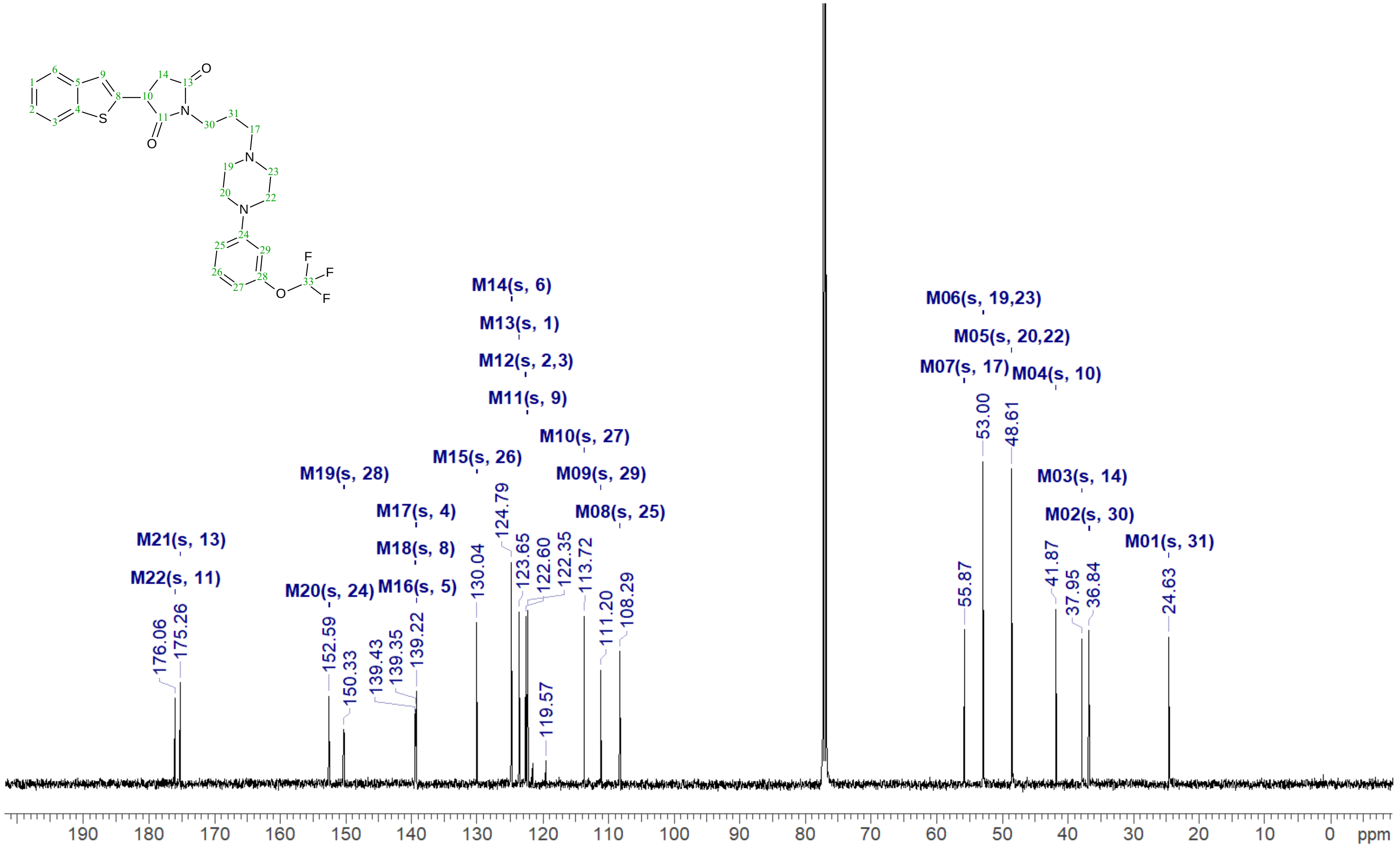

3-(Benzo[b]thiophen-2-yl)-1-(3-(4-(3-(trifluoromethoxy)phenyl)piperazin-1-yl)propyl)pyrrolidine-2,5-dione (28) – ^{19}F NMR

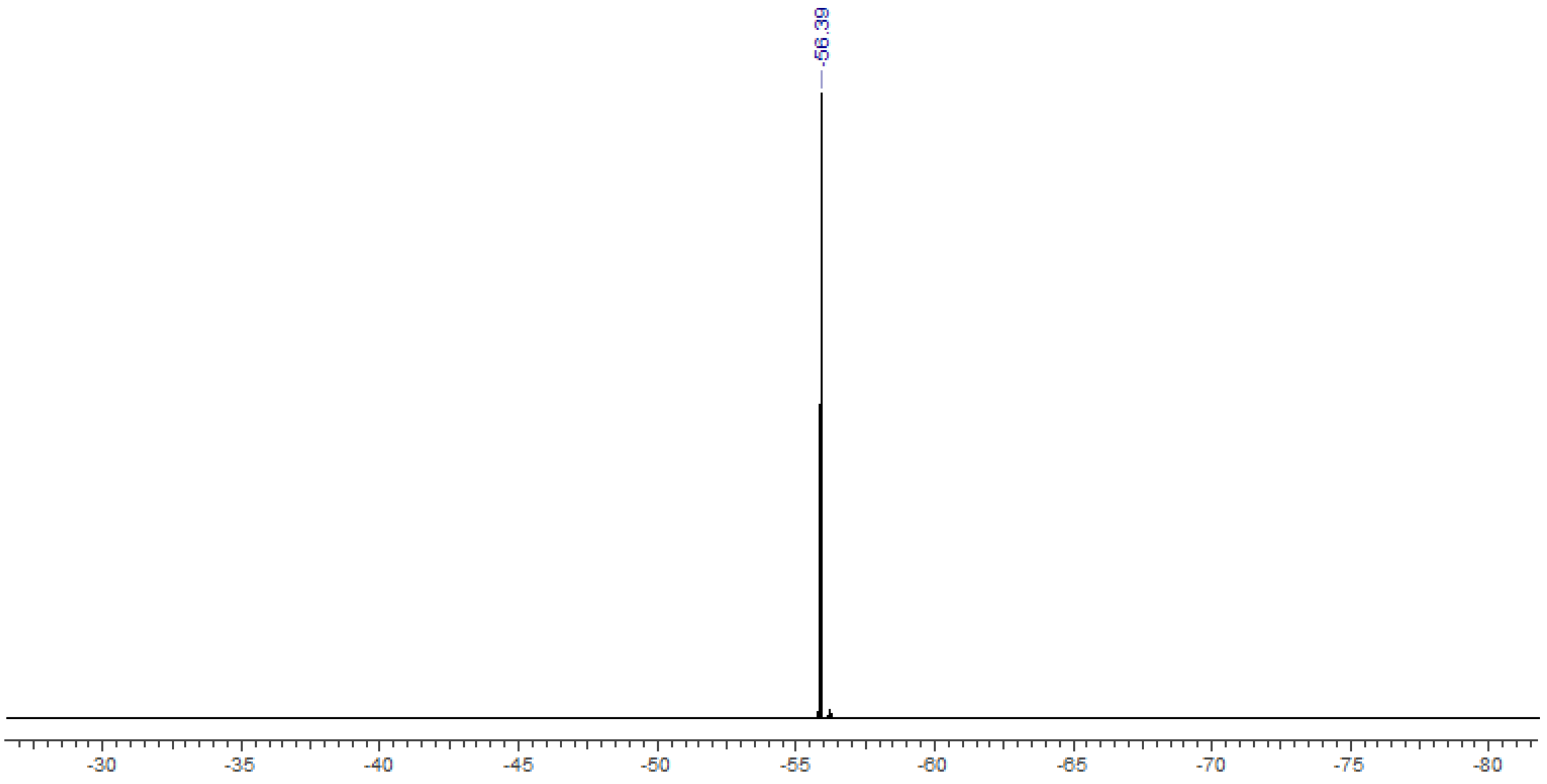

3-(Benzo[b]thiophen-2-yl)-1-(2-(4-(3-((trifluoromethyl)thio)phenyl)piperazin-1-yl)ethyl)pyrrolidine-2,5-dione (**29**) – ^{1}H NMR

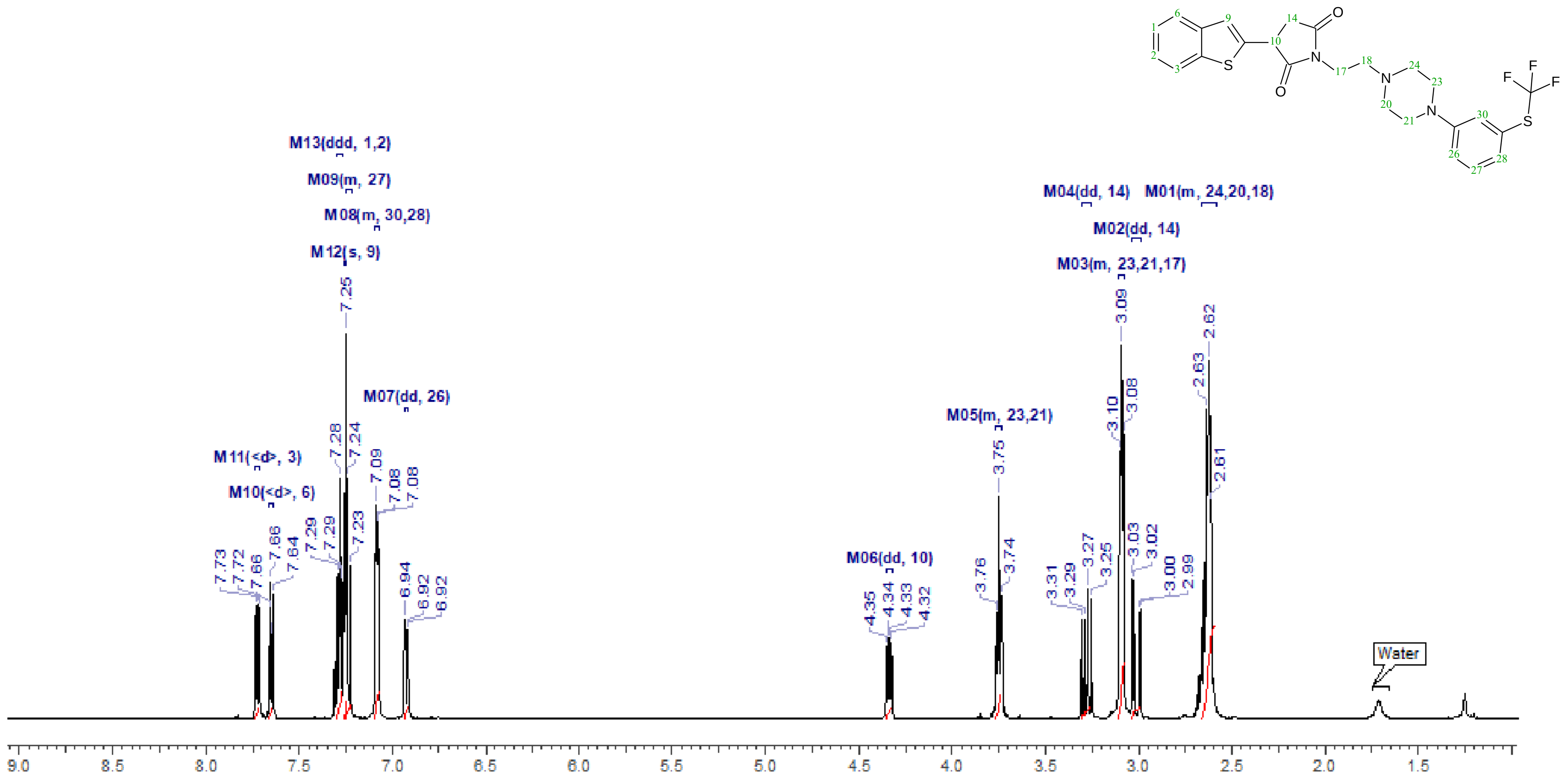

3-(Benzo[b]thiophen-2-yl)-1-(2-(4-(3-((trifluoromethyl)thio)phenyl)piperazin-1-yl)ethyl)pyrrolidine-2,5-dione (**29**) – ^{13}C NMR

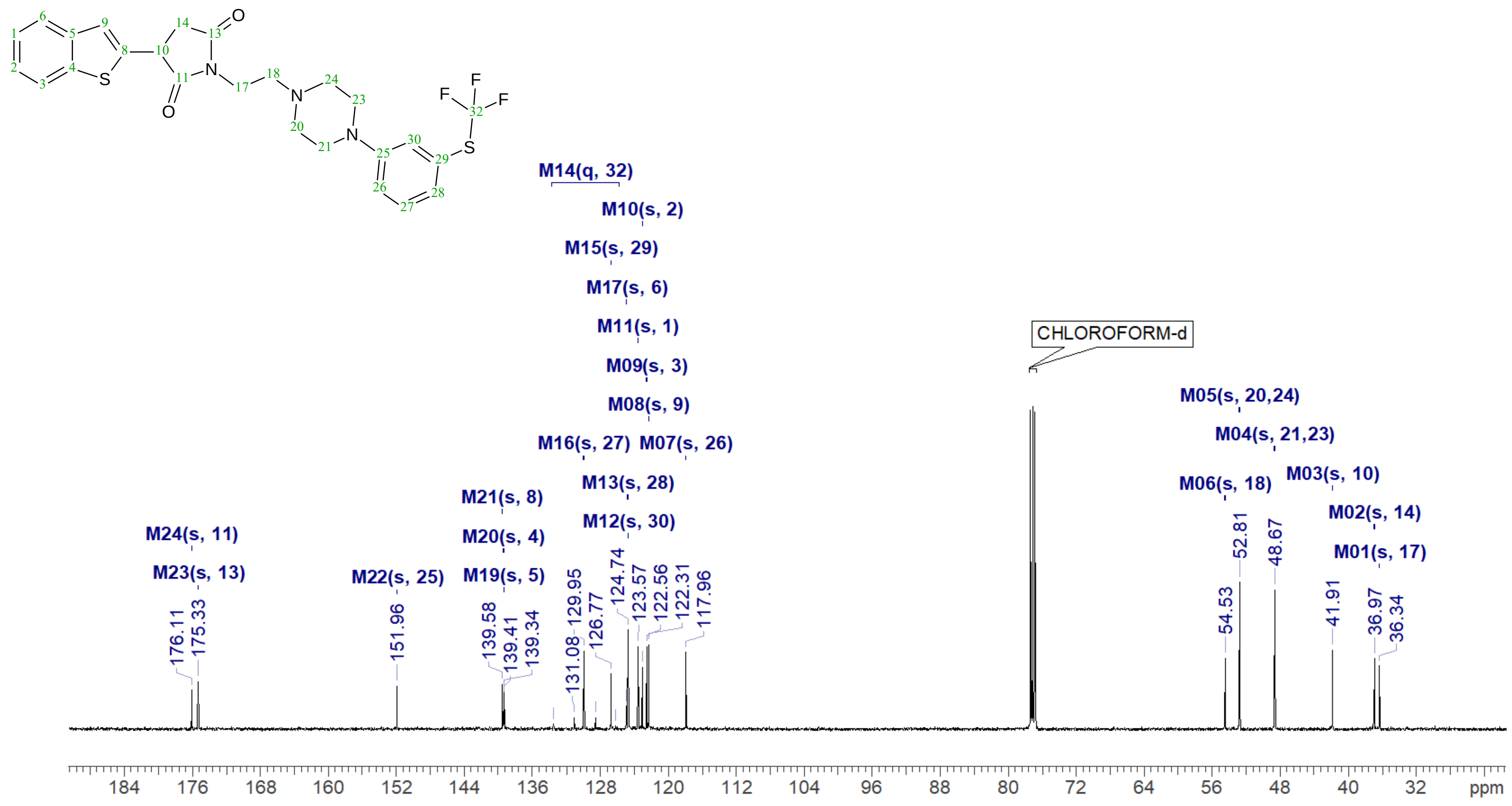

3-(Benzo[b]thiophen-2-yl)-1-(2-(4-(3-((trifluoromethyl)thio)phenyl)piperazin-1-yl)ethyl)pyrrolidine-2,5-dione (**29**) – ^{19}F NMR

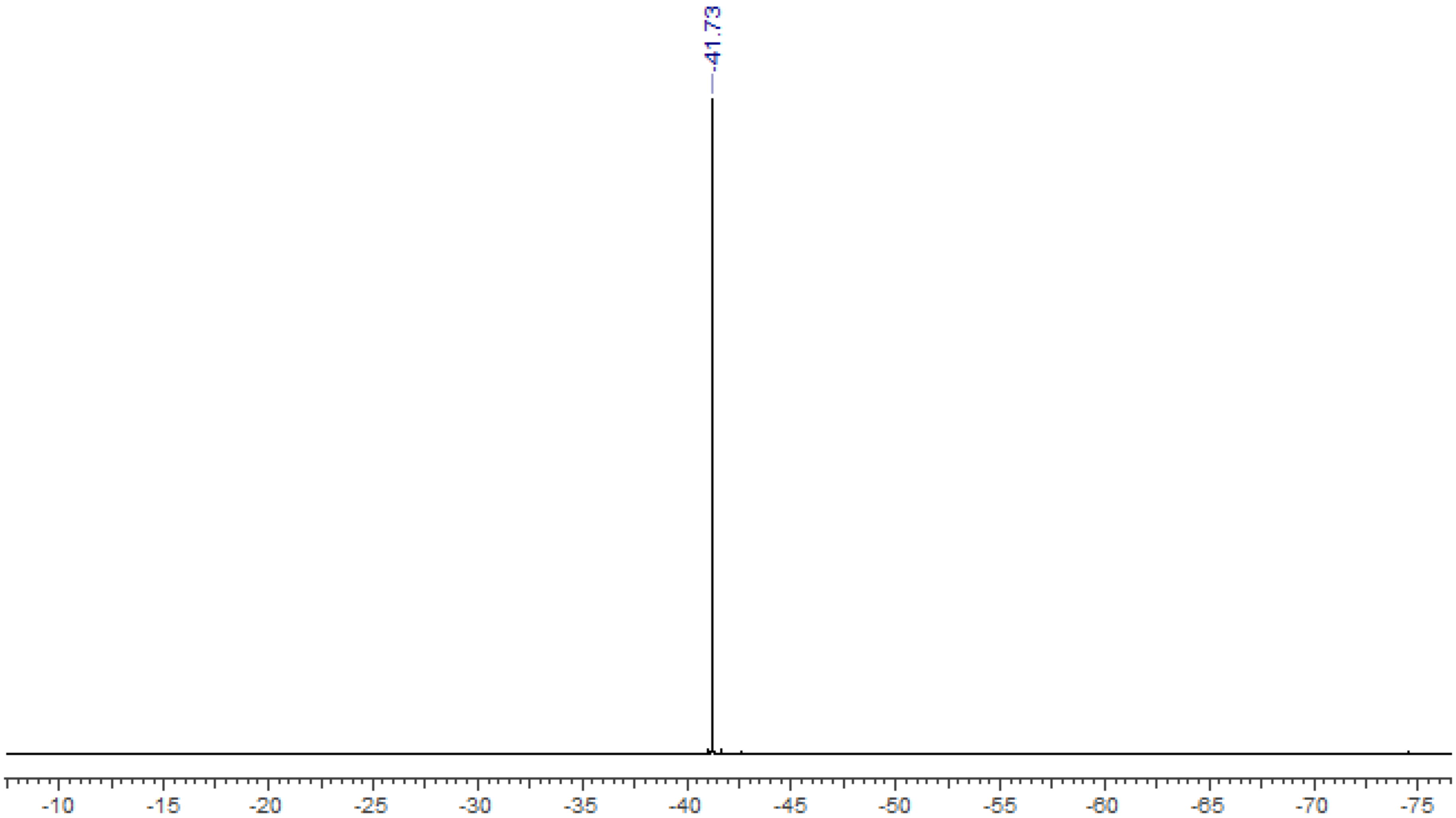

3-(Benzo[b]thiophen-2-yl)-1-(3-(4-(3-((trifluoromethyl)thio)phenyl)piperazin-1-yl)propyl)pyrrolidine-2,5-dione (30) – ^{1}H NMR

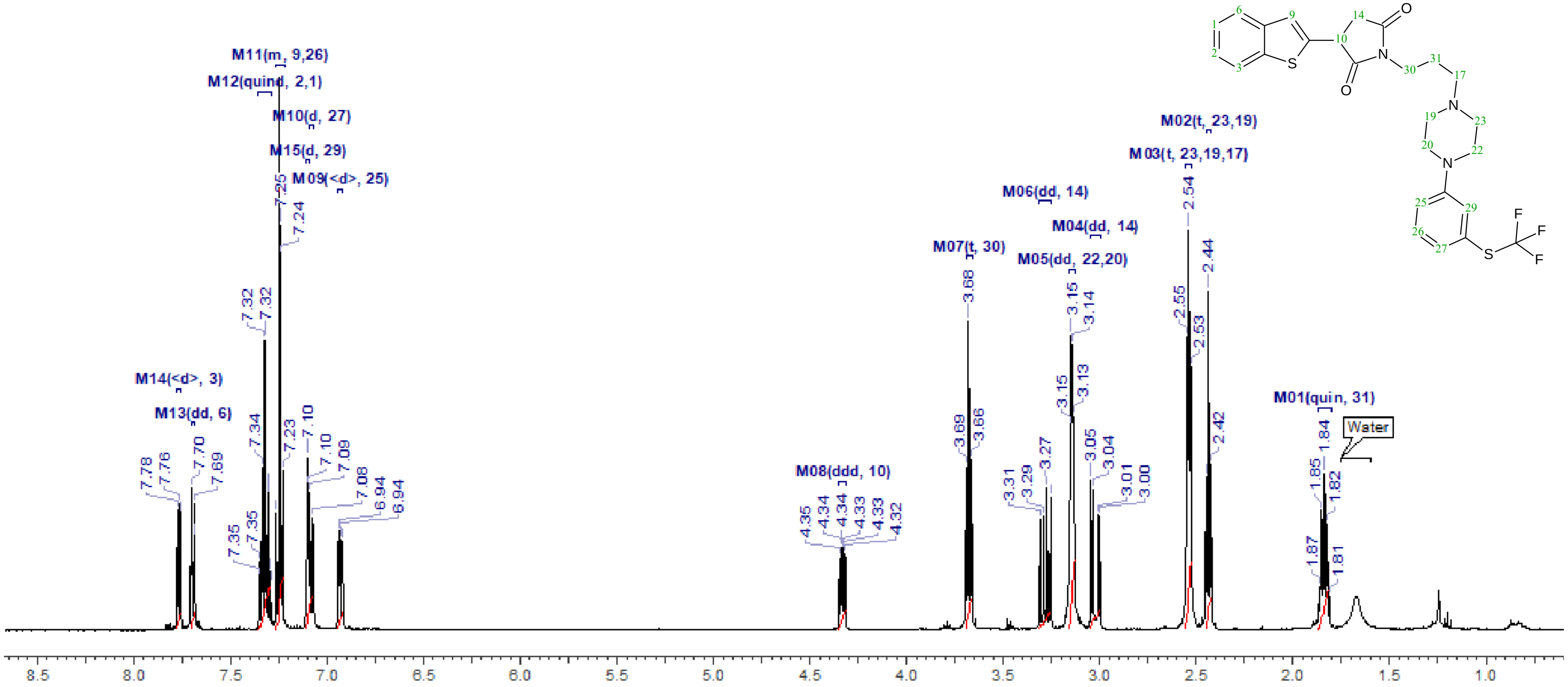

3-(Benzo[b]thiophen-2-yl)-1-(3-(4-(3-((trifluoromethyl)thio)phenyl)piperazin-1-yl)propyl)pyrrolidine-2,5-dione (30) – ^{13}C NMR

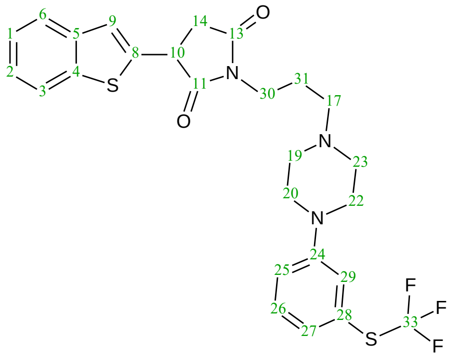

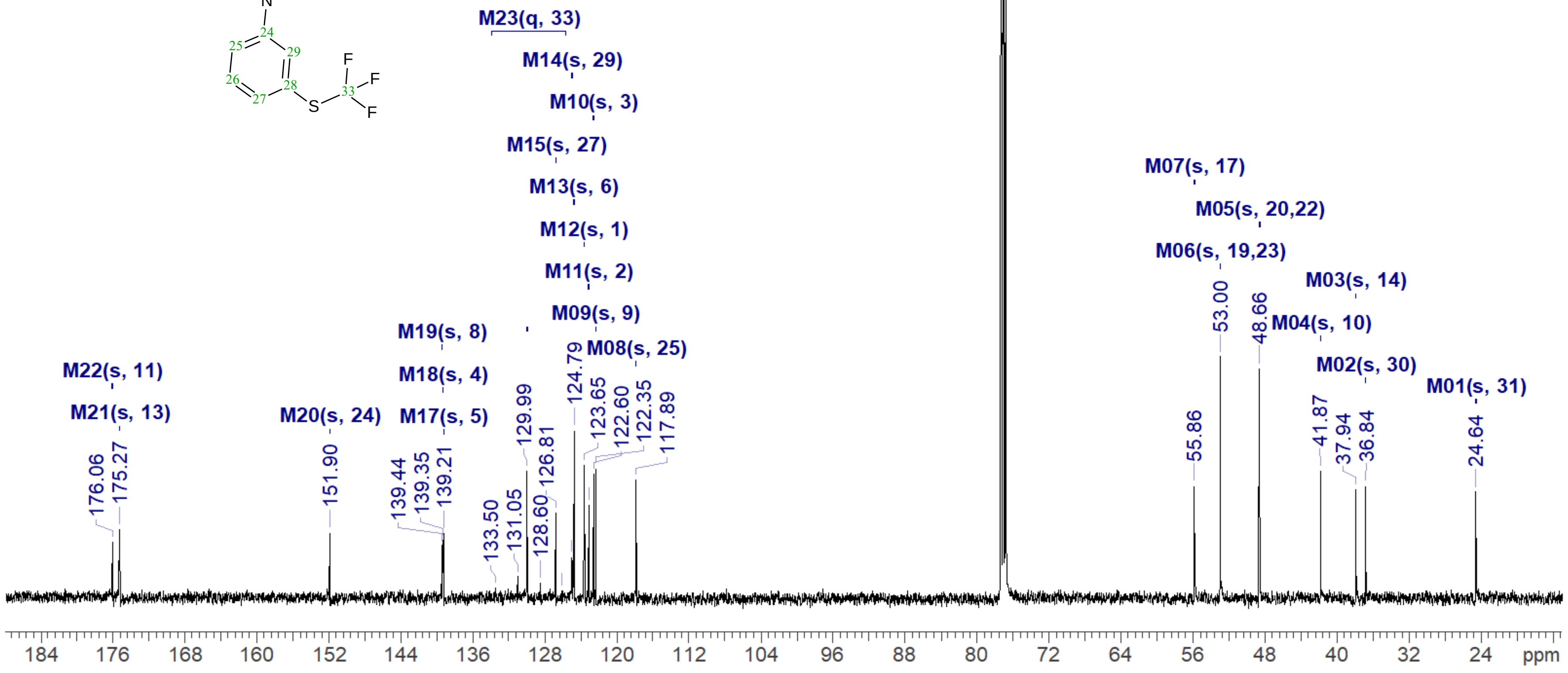

3-(Benzo[b]thiophen-2-yl)-1-(3-(4-(3-((trifluoromethyl)thio)phenyl)piperazin-1-yl)propyl)pyrrolidine-2,5-dione (30) – ^{19}F NMR

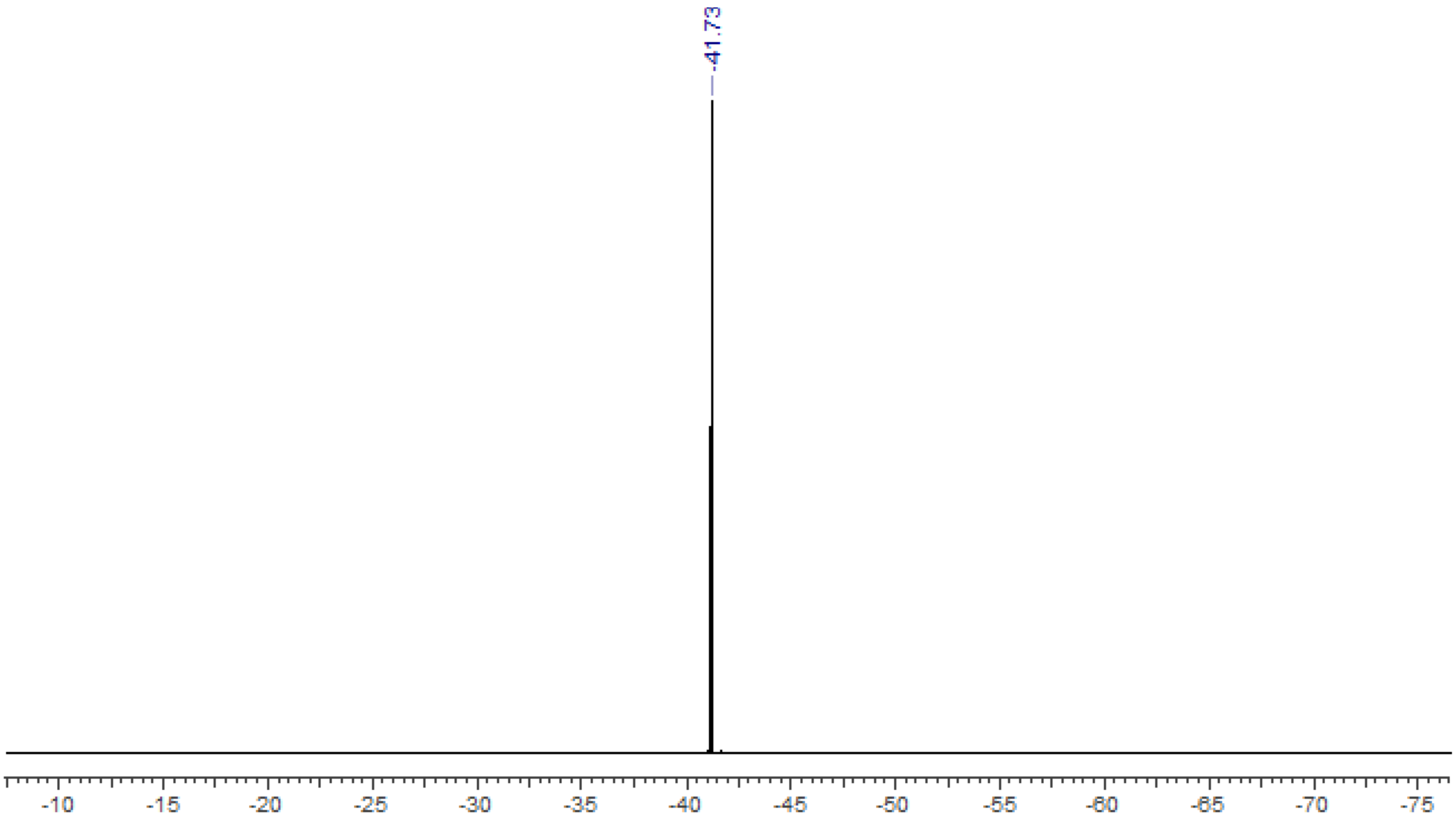

3-(Benzofuran-2-yl)-1-(3-(4-(3-(trifluoromethoxy)phenyl)piperazin-1-yl)propyl)pyrrolidine-2,5-dione (31) – ^{1}H NMR

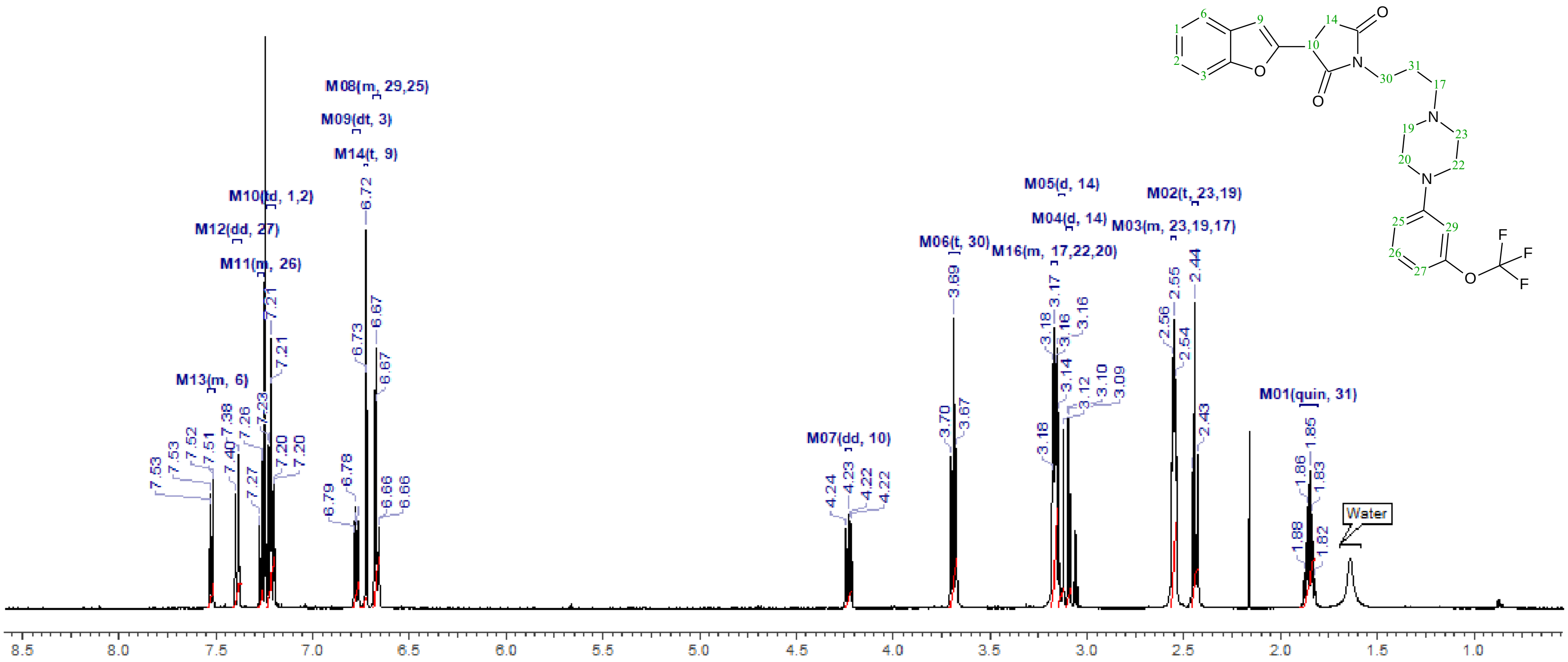

3-(Benzofuran-2-yl)-1-(3-(4-(3-(trifluoromethoxy)phenyl)piperazin-1-yl)propyl)pyrrolidine-2,5-dione (31) – ^{13}C NMR

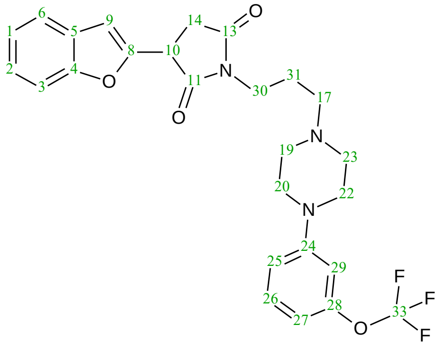

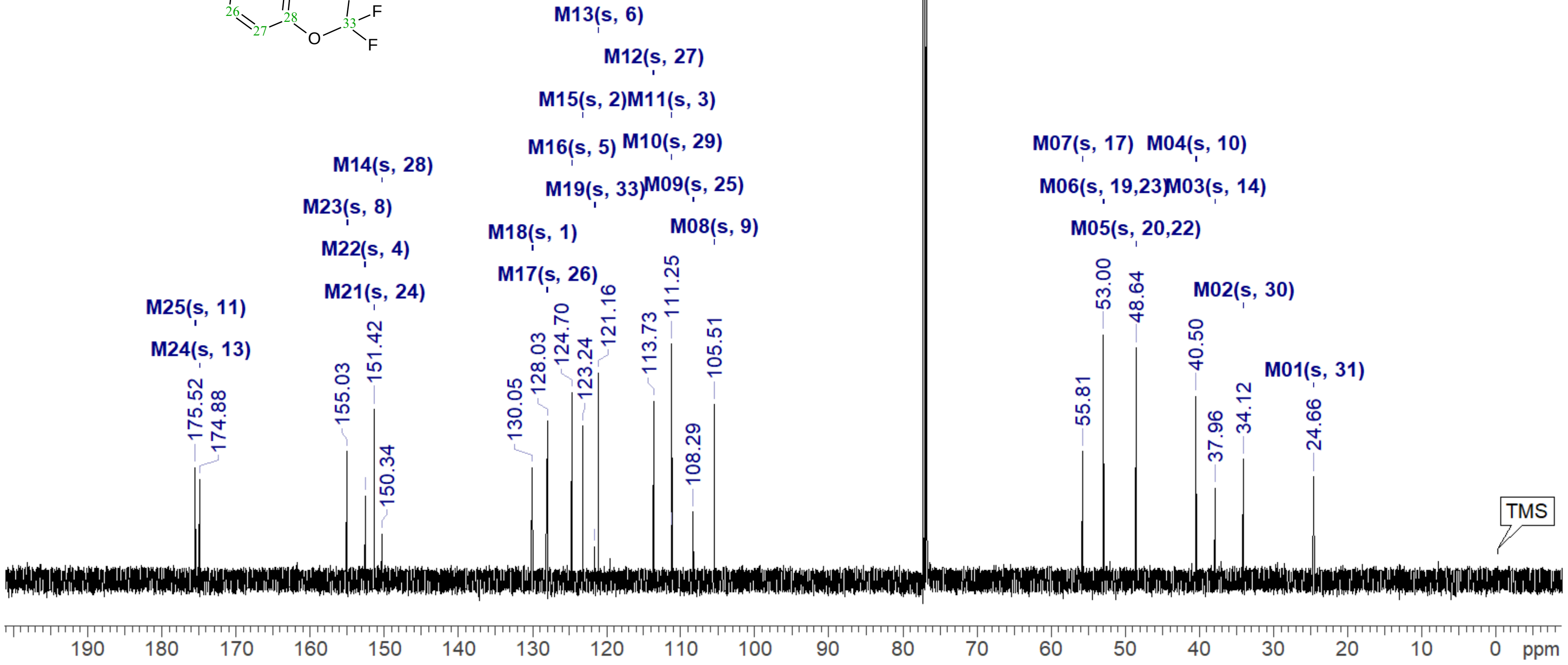

3-(Benzofuran-2-yl)-1-(3-(4-(3-(trifluoromethoxy)phenyl)piperazin-1-yl)propyl)pyrrolidine-2,5-dione (31) – ^{19}F NMR

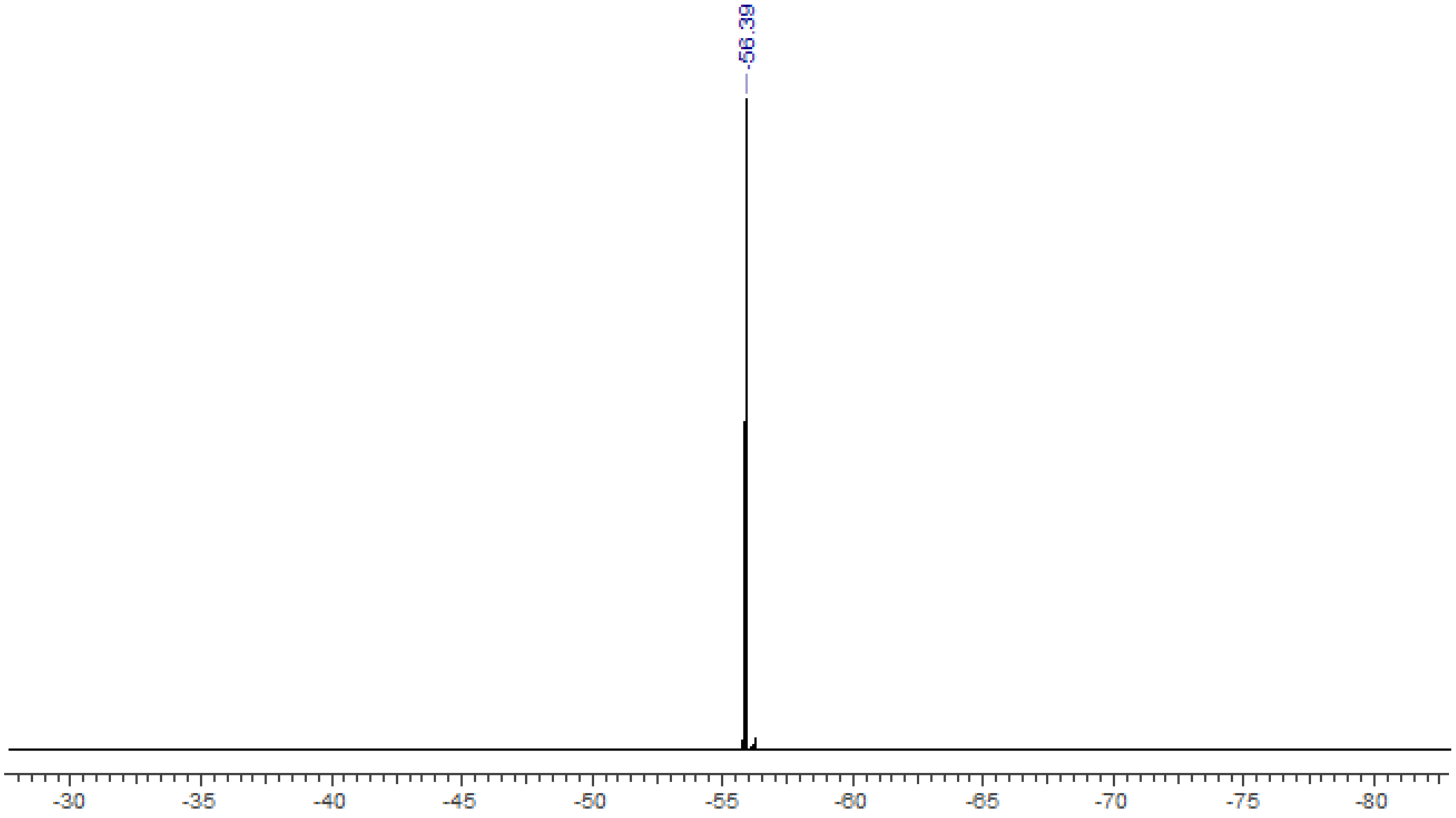

3-(Benzo[b]thiophen-2-yl)-1-(2-morpholinoethyl)pyrrolidine-2,5-dione (32) – ^{1}H NMR

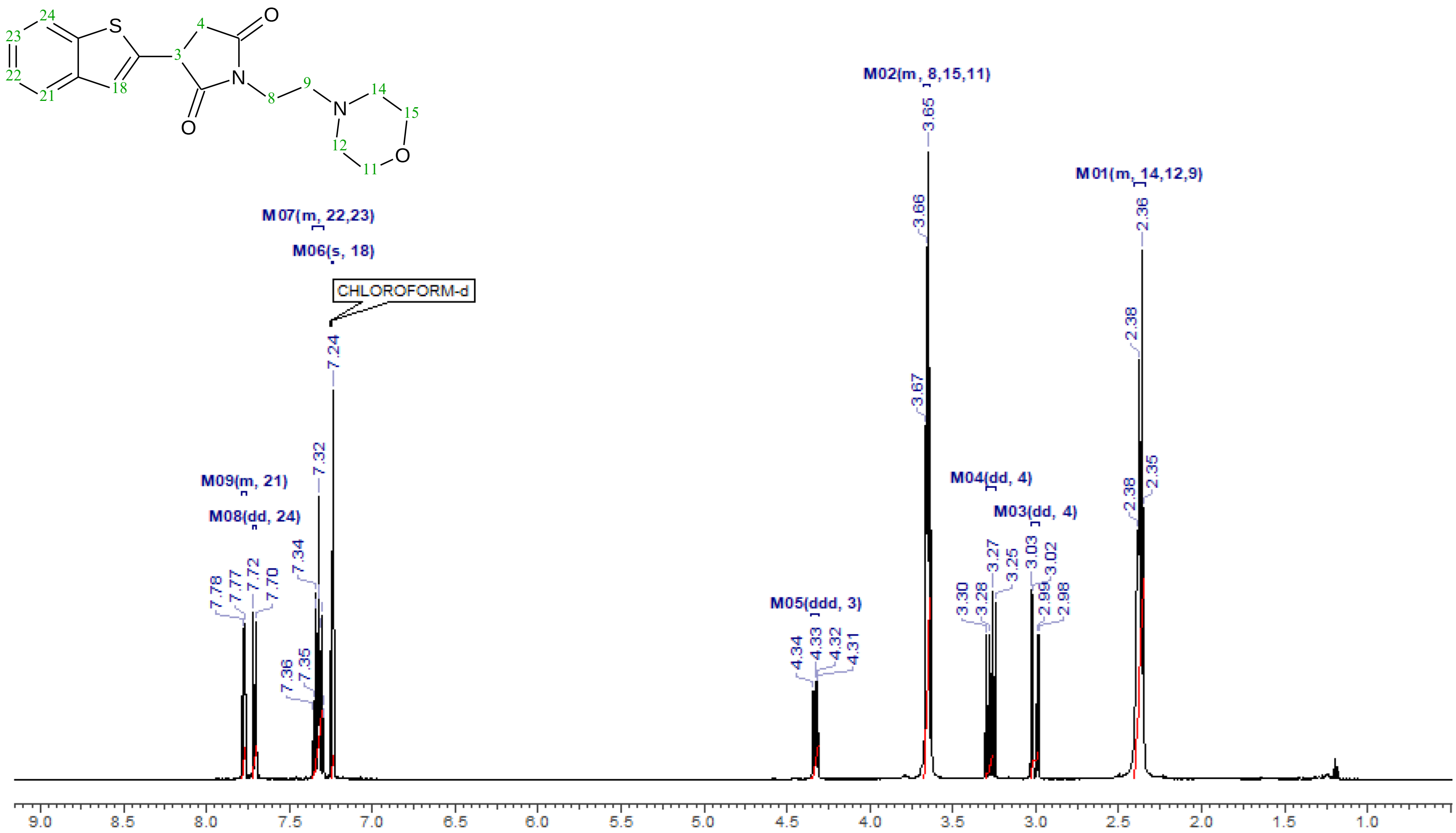

3-(Benzo[b]thiophen-2-yl)-1-(2-morpholinoethyl)pyrrolidine-2,5-dione (32) – ^{13}C NMR

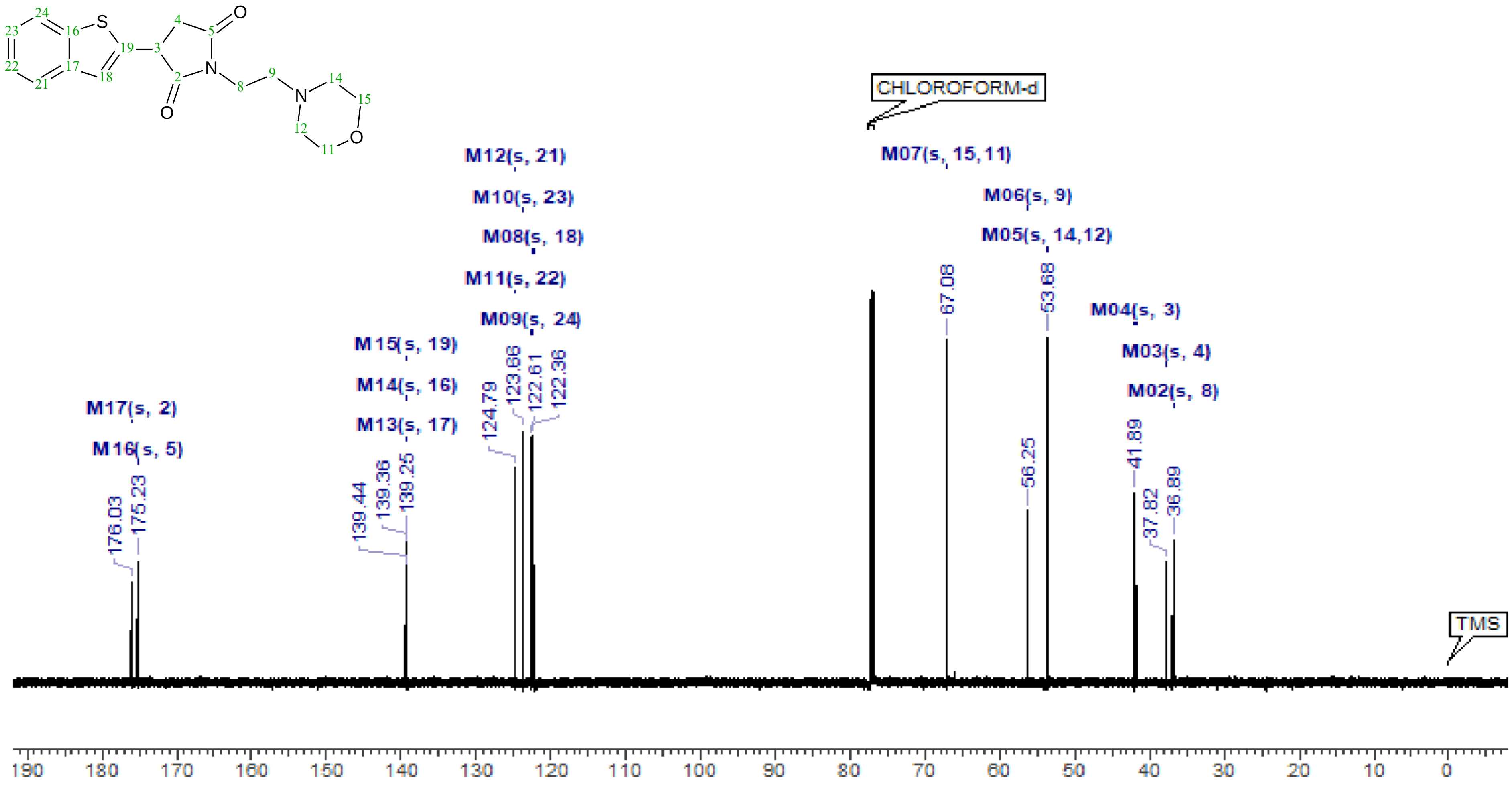

3-(Benzo[b]thiophen-2-yl)-1-(3-morpholinopropyl)pyrrolidine-2,5-dione (33) – ^{1}H NMR

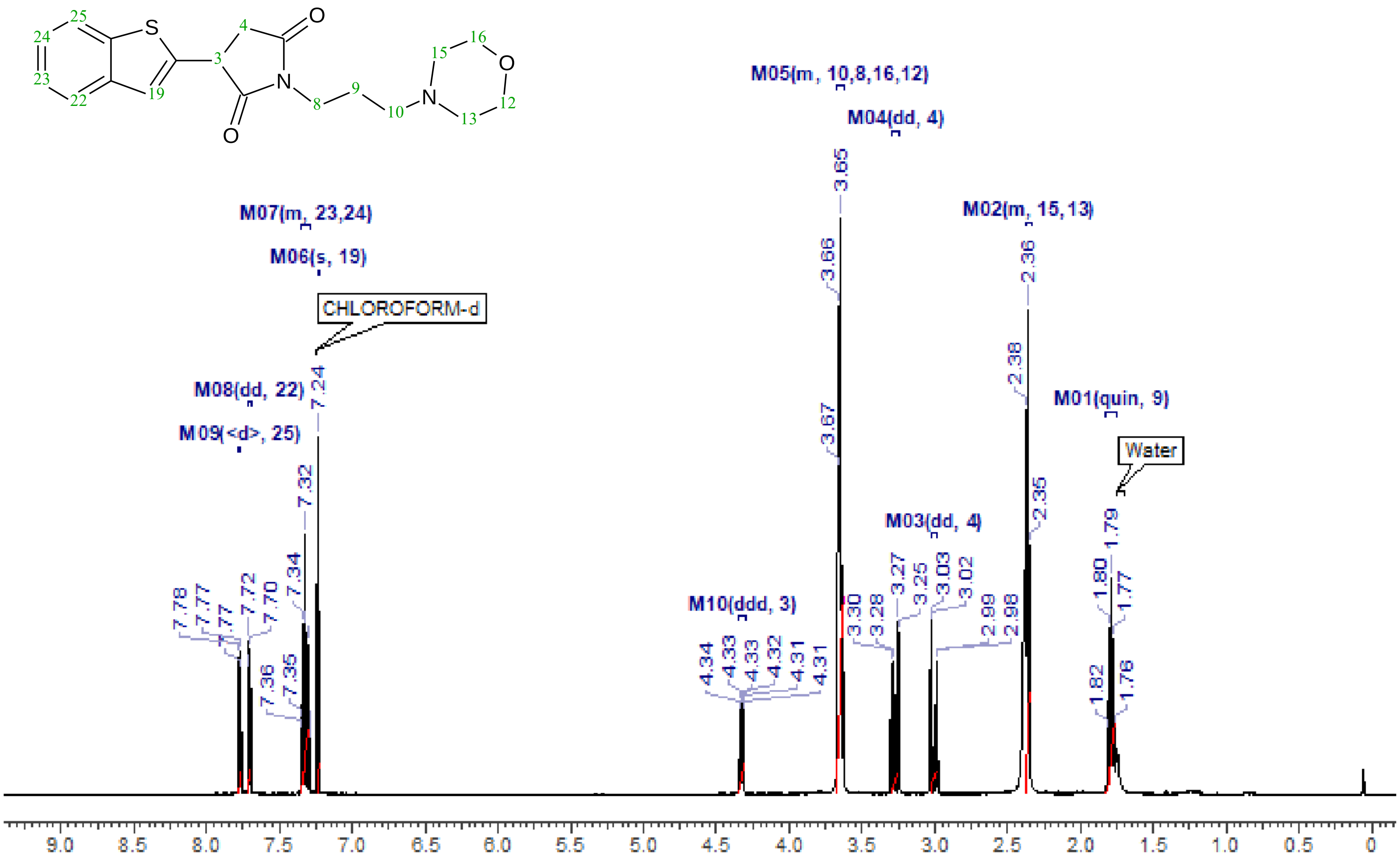

3-(Benzo[b]thiophen-2-yl)-1-(3-morpholinopropyl)pyrrolidine-2,5-dione (33) – ^{13}C NMR

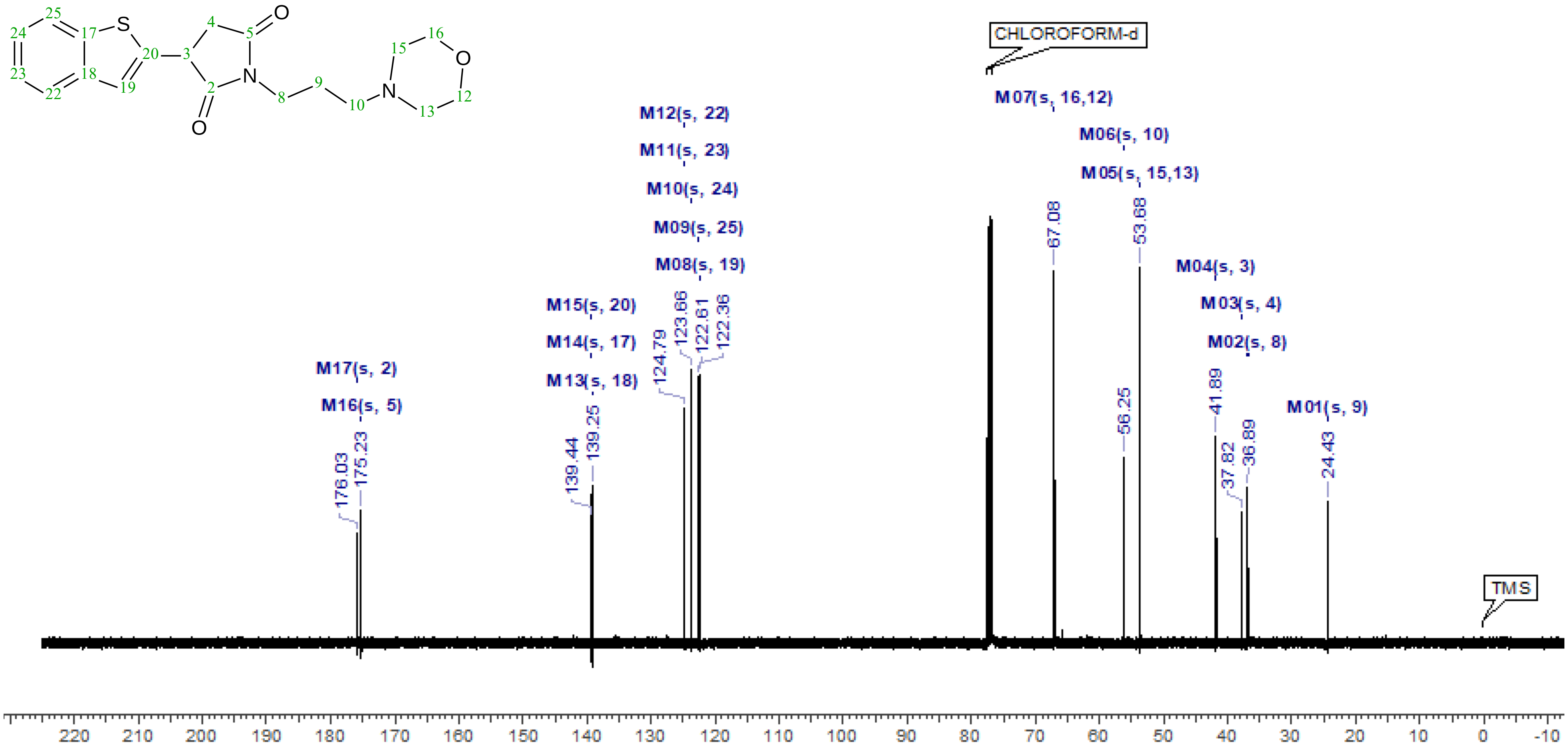

Supplement: Supplementary file 1 [file pharmaceuticals-17-01532-s001.zip › pharmaceuticals-3278633-supplementary.pdf]
